# MicroRNAs Profiling in Murine Models of Acute and Chronic Asthma: A Relationship with mRNAs Targets

**DOI:** 10.1371/journal.pone.0016509

**Published:** 2011-01-28

**Authors:** Nancy Garbacki, Emmanuel Di Valentin, Vân Anh Huynh-Thu, Pierre Geurts, Alexandre Irrthum, Céline Crahay, Thierry Arnould, Christophe Deroanne, Jacques Piette, Didier Cataldo, Alain Colige

**Affiliations:** 1 GIGA-Research, Laboratory of Connective Tissues Biology, University of Liège, Liège, Belgium; 2 GIGA-Research, Laboratory of Virology and Immunology, University of Liège, Liège, Belgium; 3 GIGA-Research, Systems and modeling, University of Liège, Liège, Belgium; 4 GIGA-Research, Laboratory of Biology of Tumours and Development, University of Liège, Liège, Belgium; 5 Laboratory of Biochemistry and Cell Biology, University of Namur, Namur, Belgium; Baylor College of Medicine, United States of America

## Abstract

**Background:**

miRNAs are now recognized as key regulator elements in gene expression. Although they have been associated with a number of human diseases, their implication in acute and chronic asthma and their association with lung remodelling have never been thoroughly investigated.

**Methodology/Principal Findings:**

In order to establish a miRNAs expression profile in lung tissue, mice were sensitized and challenged with ovalbumin mimicking acute, intermediate and chronic human asthma. Levels of lung miRNAs were profiled by microarray and *in silico* analyses were performed to identify potential mRNA targets and to point out signalling pathways and biological processes regulated by miRNA-dependent mechanisms. Fifty-eight, 66 and 75 miRNAs were found to be significantly modulated at short-, intermediate- and long-term challenge, respectively. Inverse correlation with the expression of potential mRNA targets identified mmu-miR-146b, -223, -29b, -29c, -483, -574-5p, -672 and -690 as the best candidates for an active implication in asthma pathogenesis. A functional validation assay was performed by cotransfecting in human lung fibroblasts (WI26) synthetic miRNAs and engineered expression constructs containing the coding sequence of luciferase upstream of the 3′UTR of various potential mRNA targets. The bioinformatics analysis identified miRNA-linked regulation of several signalling pathways, as matrix metalloproteinases, inflammatory response and TGF-β signalling, and biological processes, including apoptosis and inflammation.

**Conclusions/Significance:**

This study highlights that specific miRNAs are likely to be involved in asthma disease and could represent a valuable resource both for biological makers identification and for unveiling mechanisms underlying the pathogenesis of asthma.

## Introduction

Asthma is a complex chronic inflammatory disease characterized by eosinophilic airway inflammation, reversible airway obstruction and hyper-responsiveness. Moreover, asthma patients display a faster rate of lung dysfunction than normal individuals related to a progressive remodelling of airway walls [1], [2]. Major features of this remodelling process consist of epithelial damages, smooth muscle cell hyperplasia, glandular hyperplasia and airway wall fibrosis including a thin collagen layer deposition in the lamina reticularis of airway epithelium [2], [3]. To date, marketed asthma treatments are not sufficient to allow a satisfactory level of control in every patient and are not effective towards airways remodelling processes. For these reasons, identification of new therapeutic targets and new biomarkers relevant for diagnosis and prognosis evaluation are eagerly needed.

The evolution of the disease from acute inflammation to the fibrotic process is driven by soluble factors, such as cytokines and chemokines, and by cell-cell and cell-matrix interactions. An abundant literature [4]–[10] shows that these regulatory signals affect the level of transcription of several genes in leukocytes and in specific lung cells. These transcriptomic studies led to the identification of several potential new therapeutic targets but largely failed to provide information about regulations operated at a post-transcriptional level. MicroRNA-driven RNA interference is a newly recognized and evolutionary conserved regulatory mechanism. MiRNAs lead to the degradation and/or repression of translation of specific mRNAs depending on the type of base-pairing between the miRNA and its mRNA target [11]. In mammals miRNA-dependent regulation mostly affects the translation process although moderate and variable levels of degradation of the target are also often associated. Most interestingly, this opens the possibility of inferring the existence of potent miRNA-driven translational regulation from powerful and validated whole genome transcriptomic studies. Taking into account that each mammal genome contains several hundreds of miRNA [12], that the complementarities between a miRNA and its target sequence are usually not perfect, that a single miRNA may regulate the translation/degradation of many mRNAs and that a single mRNA can be affected by several different miRNAs, one can consider that this type of investigation requires the use of powerful bioinformatics tools able to analyse such complex pattern of interactions. Although miRNA expression modulations have already been associated with several human diseases [13]–[30], only few studies have explored the role of miRNAs in non-tumoral lung diseases [31]–[38] and very little is known about miRNAs expression profiles in the lung during the course of asthma and asthma-induced airway remodelling.

In the present study, microarray analysis has been used to determine miRNAs expression profiles in a mouse model of asthma designed to investigate the development of both acute and chronic responses to allergen. Moreover, *in silico* analysis and comparison with the expression profile of mRNAs in the same pathological state identified several genes and pathways that are both involved in asthma and post-transcriptionally regulated by miRNA-dependent processes. The best candidates were validated by functional assays in cell culture.

## Results and Discussion

### Assessment of airway inflammation, sensitization and hyperresponsiveness in mouse model of asthma

Mouse models of asthma are commonly considered as reliable tools to study pathological mechanisms of the disease since these animals, if adequately sensitized and exposed to allergens, develop measurable airway hyperresponsiveness, airway inflammation, and finally airway remodeling if the exposure to allergens persists [39]–[41].

**Figure 1:**
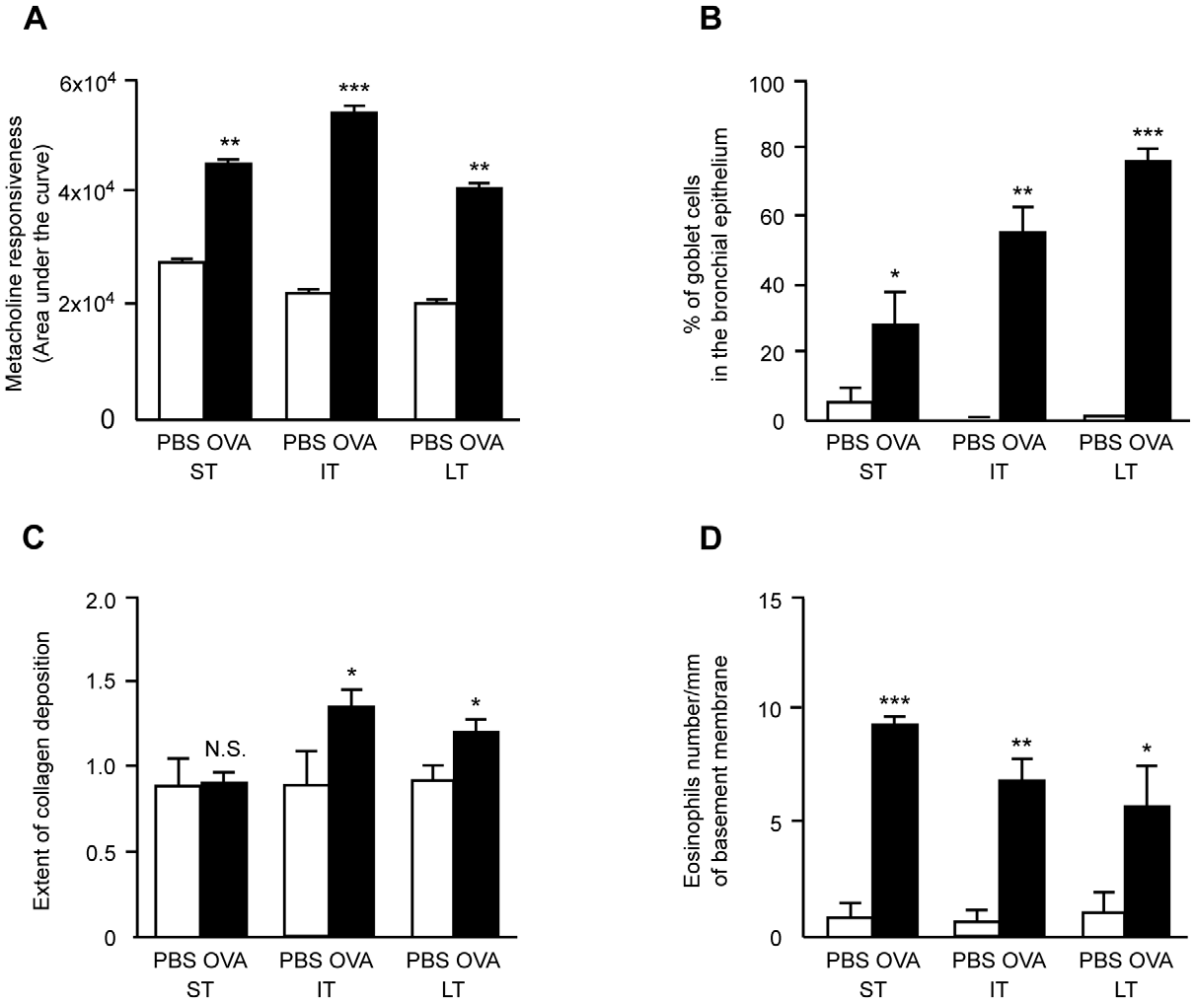
Assessment of airway inflammation, sensitization and hyperresponsiveness. Assessment of airway responsiveness to metacholine (Panel A), of glandular hyperplasia as percentage of goblet cells per total epithelial cells (Panel B), of peribronchial collagen deposition (Panel C) and of eosinophils accumulation (Panel D) in randomly selected bronchi in PBS and OVA-treated groups of mice at short-term (ST), intermediate-term (IT) and long-term (LT) sensitization and exposure protocols. Mean scores were measured as described in [4]. Results are expressed as means ± SE and the comparison between groups was performed using Mann-Whitney *U* test (* *p-value* <0.05; ** *p-value* <0.005; *** *p-value* <0.001; N.S.: not significant).

The mouse model of asthma used in this study has previously been characterized [4]. In comparison to control mice, animals exposed to allergen showed an enhanced airway resistance after increasing doses of metacholine as compared with the corresponding PBS-exposed groups (Figure 1A). Percentages of goblet cells in the bronchial epithelium (Figure 1B) and subepithelial collagen deposition (Figure 1C) did increase over time in allergen-exposed mice. Eosinophilic infiltration measured in bronchial walls was significantly increased in each allergen-exposed group as compared to controls while the eosinophils density in the bronchial walls decreased over time (Figure 1D). Exposure to aerosolized OVA induced a slight increase in differential cell numbers present in the BAL fluids when compared to their PBS-exposed counterparts (Table 1) which was expected from previous studies [40], [42] and largely resulted from eosinophilic representation.

**Table 1:** Differential cell counts in BAL fluids.

	ST		IT		LT	
	PBS	OVA	PBS	OVA	PBS	OVA
Epithelial cells (×10^4^/ml)	4.9±0.8	4.7±1.9	1.8±0.8	3.5±0.7	1.5±0.2	3.8±0.5
Eosinophils (×10^4^/ml)	5.2±1.7	75.0±30.7	1.6±0.4	8.1±1.5	2.3±0.4	11.3±1.8
Lymphocytes (×10^4^/ml)	0.2±0.1	0.5±0.1	0.1±0.0	0.2±0.1	0.2±0.1	0.5±0.1
Macrophages (×10^4^/ml)	36.9±6.0	22.1±6.1	17.6±1.1	22.9±3.6	26.9±3.1	21.5±1.9
Neutrophils (×10^4^/ml)	0.1±0.0	2.1±0.1	0.1±0.0	0.6±0.2	0.2±0.1	1.1±0.2

Cellular composition of BAL fluid and absolute cell counts performed 24 hours after the last allergen or PBS exposure. ST, IT, LT: short, intermediate and long-term treatments, respectively.

Reproducibility of our mouse model of asthma induction was further assessed by measuring in each individual mouse of the current study. The relative abundance of 8 transcripts known from a previous study [4] to be regulated at the 3 time-points of allergen exposure (ST, IT and LT). The new data set is fully in agreement with previous quantifications, except for Cdc2a at ST and LT (Figure S1). All together these functional, cellular and transcriptomic evaluations demonstrate the reproducibility of our models of asthma from one experiment to another. These data allow us to use the transcriptomic data from our previous study [4] to relate mRNA targets with the miRNAs identified in this study. However, the animal model of asthma used here can also bear per se some limitations. For instance, allergen used in animals (ovalbumin) is not a causative agent of human asthma in physiological conditions. The animals are also anatomically different from human regarding bronchial architecture since number of bronchial divisions is lower in mice. Nevertheless, this model with its intrinsic limitations allowed the investigators to reproduce all key features of asthmatic airways as found in humans with an allergen-induced airway inflammation and hyperresponsiveness as well as an allergen-induced airway remodeling (figure 1A–D).

### MiRNA expression changed in lungs during allergen exposure

Lung total RNA was obtained from groups of mice exposed to allergen (OVA) or control (PBS) for 1 (ST), 5 (IT) and 10 (LT) weeks. Microarray analysis indicated that 58, 66 and 75 mature miRNAs out of 566 were significantly (*p-value* <0.01) modulated at ST, IT and LT, respectively (Table S1). For many of them, the fold induction or repression in the OVA-treated mice was higher than 1.5 (20 at ST, 26 at IT and 67 at LT) (Table 2). Some miRNAs underwent a significant modulation (≥1.5-fold, *p-value* <0.01) at two time-points but only one, mmu-miR-146b, was consistently upregulated at the three investigated time-points (Table 3).

**Table 2:** List of significantly modulated mature miRNAs (≥1.5-fold, *p-value* <0.01) and their respective fold induction at each time-point.

	Upregulation		Downregulation	
	miRNA	FI	miRNA	FI
**ST**				
	mmu-miR-712*	5.24	mmu-miR-187	0.66
	mmu-miR-122	5.08	mmu-miR-497	0.49
	mmu-miR-181d	2.51	mmu-miR-690	0.49
	mmu-miR-106a	2.28	mmu-miR-1	0.46
	mmu-miR-223	1.99	mmu-miR-483	0.39
	mmu-miR-146b	1.91	mmu-miR-574-5p	0.37
	mmu-miR-181b	1.91	mmu-miR-203	0.35
	mmu-miR-689	1.90	mmu-miR-672	0.35
	mmu-miR-20b	1.88	mmu-miR-805	0.28
	mmu-miR-451	1.60		
	mmu-miR-100	1.52		
**IT**				
	mmu-miR-155	4.48	mmu-miR-322	0.66
	mmu-miR-467b*	2.97	mmu-miR-429	0.66
	mmu-miR-467a*	2.79	mmu-miR-199a-3p	0.63
	mmu-miR-466g	2.64	mmu-miR-152	0.63
	mmu-miR-466f-3p	2.23	mmu-miR-29c	0.62
	mmu-miR-455	2.03	mmu-miR-218	0.62
	mmu-miR-150	1.80	mmu-miR-200a	0.59
	mmu-miR-423-5p	1.69	mmu-miR-10a	0.59
	mmu-miR-146b	1.55	mmu-miR-10b	0.53
	mmu-miR-375	1.52	mmu-miR-29b	0.52
			mmu-miR-101a	0.36
			mmu-miR-223	0.32
			mmu-miR-19b	0.31
			mmu-miR-690	0.24
			mmu-miR-450a-5p	0.19
			mmu-miR-126-5p	0.14
**LT**				
	mmu-miR-705	119.30	mmu-miR-200b	0.61
	mmu-miR-188-5p	117.39	mmu-miR-92a	0.60
	mmu-miR-483	115.80	mmu-miR-30c	0.59
	mmu-miR-669c	115.25	mmu-miR-27a	0.53
	mmu-miR-568	96.03	mmu-let-7e	0.50
	mmu-miR-467b*	48.35	mmu-miR-21	0.47
	mmu-miR-691	42.50	mmu-miR-25	0.46
	mmu-miR-671-5p	39.54	mmu-miR-30b	0.44
	mmu-miR-467a*	39.02	mmu-miR-23b	0.41
	mmu-miR-485*	33.41	mmu-miR-23a	0.38
	mmu-miR-744	29.56	mmu-miR-26b	0.31
	mmu-miR-466f-3p	22.69	mmu-miR-98	0.20
	mmu-miR-685	19.58	mmu-miR-15a	0.19
	mmu-miR-709	18.98	mmu-miR-29c	0.11
	mmu-miR-467e*	18.51		
	mmu-miR-466c-5p	17.07		
	mmu-miR-466g	16.19		
	mmu-miR-574-3p	15.99		
	mmu-miR-574-5p	13.18		
	mmu-miR-667	13.13		
	mmu-miR-713	11.83		
	mmu-let-7d*	11.72		
	mmu-miR-762	11.22		
	mmu-miR-466d-3p	9.95		
	mmu-miR-466b-3-3p	9.89		
	mmu-miR-466f-5p	9.50		
	mmu-miR-297a*	9.21		
	mmu-miR-468	8.99		
	mmu-miR-466a-3p	8.48		
	mmu-miR-197	8.06		
	mmu-miR-455	7.51		
	mmu-miR-877*	6.41		
	mmu-miR-297a	6.38		
	mmu-miR-15a*	6.14		
	mmu-miR-207	6.03		
	mmu-miR-346	5.84		
	mmu-miR-466h	5.54		
	mmu-miR-206	5.48		
	mmu-miR-328	5.38		
	mmu-miR-672	5.30		
	mmu-miR-214	5.29		
	mmu-miR-320	4.52		
	mmu-miR-34c*	4.26		
	mmu-miR-423-5p	4.04		
	mmu-miR-674	3.84		
	mmu-miR-151-3p	3.18		
	mmu-miR-143	2.86		
	mmu-miR-146b	2.69		
	mmu-miR-720	2.69		
	mmu-miR-146a	2.09		
	mmu-miR-99b	2.03		
	mmu-miR-125b-5p	1.88		
	mmu-miR-145	1.75		
	mmu-miR-30d	1.64		
	mmu-miR-191	1.59		

ST, IT, LT: short, intermediate and long-term treatments, respectively; FI: fold induction.

**Table 3:** miRNAs undergoing a significant modulation (≥ 1.5-fold, *p-value* <0.01) determined by microarray analysis at, at least, 2 time-points.

	Fold Induction		
	ST	IT	LT
mmu-miR-690	0.49	0.24	N.C.
mmu-miR-223	1.99	0.32	N.C.
mmu-miR-672	0.35	N.C.	5.31
mmu-miR-574-5p	0.37	N.C.	13.18
mmu-miR-483	0.39	N.C.	116.16
mmu-miR-29c	N.C.	0.62	0.11
mmu-miR-423-5p	N.C.	1.69	4.03
mmu-miR-455	N.C.	2.03	7.52
mmu-miR-466f-3p	N.C.	2.23	22.63
mmu-miR-466g	N.C.	2.64	16.22
mmu-miR-467a*	N.C.	2.79	39.12
mmu-miR-467b*	N.C.	2.97	48.50
mmu-miR-146b	1.91	1.55	2.69

ST, IT, LT: short, intermediate and long-term treatments, respectively; N.C.: no change between PBS and OVA.

#### Modulated miRNAs after short term (ST) and intermediate term (IT) exposure to allergen

Mmu-miR-690 was downregulated at ST and IT but was no longer modulated at LT. In the literature, this miRNA was only reported to be significantly downregulated in pancreatic β cells after treatment with high glucose [43]. The second miRNA significantly regulated, mmu-miR-223, was previously shown to play a significant role in pulmonary function alteration by exposition to cigarette smoke [31] or to LPS [33]. It is also known to be involved in hematopoietic development [44]–[47], to be expressed by PBMC [48] and neutrophils [33] and to be implicated in malignancies such as ovarian cancer [49], hepatocellular carcinoma [50] and bladder cancer [51].

#### Modulated miRNAs after short term (ST) and long term (LT) exposure to allergen

Three miRNAs, mmu-miR-672, -574-5p and -483 were downregulated at ST and upregulated at LT. To our knowledge, only miR-483 has been previously associated to a specific disease or a physiological state and was found to be significantly correlated with cardiac hypertrophy [52].

#### Modulated miRNAs after intermediate term (IT) and long term (LT) exposure to allergen

Mmu-miR-423-5p, -455, -466f-3p, -466g, -467a* and -467b* were consistently upregulated at IT and LT. Among these miRNAs, miR-455 has been reported to be implicated in brown adipocyte differentiation [53], while the functions or targets of the others are not determined yet. These miRNAs could become interesting markers of late phases of asthma development as they are significantly upregulated. By contrast to these six miRNAs, mmu-miR-29c is downregulated at these two time-points. MiR-29 has been reported to be involved in various human cancers [54]-[57] and in tumour suppression by targeting the T-cell leukemia/lymphoma 1 (Tcl1) oncogene mRNA [54], by reverting DNA methylation by targeting DNA methyltransferases 3A (Dnmt3a) and 3B (Dnmt3b) mRNA [58] and by regulating p53 pathway through Cdc42 and p85α [59]. Moreover, its targets mRNAs encoding extracellular matrix proteins have been associated with cell migration and metastasis such as Col3a1, Col4a1, Col15a1, Lamc1 [60]. MiR-29 regulates also muscle cell differentiation probably, in part, under a feed-back control of NF-κB-YY1 pathway [61].

#### Modulated miRNAs after short term (ST), intermediate term (IT) and long term (LT) exposure to allergen

Mmu-miR-146b was the only miRNA consistently upregulated during the entire time-course of the experiment. MiR-146b is expressed by leukocytes and its function is clearly associated with inflammation and innate immunity [33], [62]. Taganov et al. [63] showed that miR-146 regulatory circuit fine-tunes TLR and cytokine signalling, rather than totally abrogating the signal, in response to microbial components and proinflammatory cytokines. Moreover, this miRNA acts as a negative regulator of NF-κB activity in a human breast cancer cell line [64].

### Microarray validation

**Figure 2:**
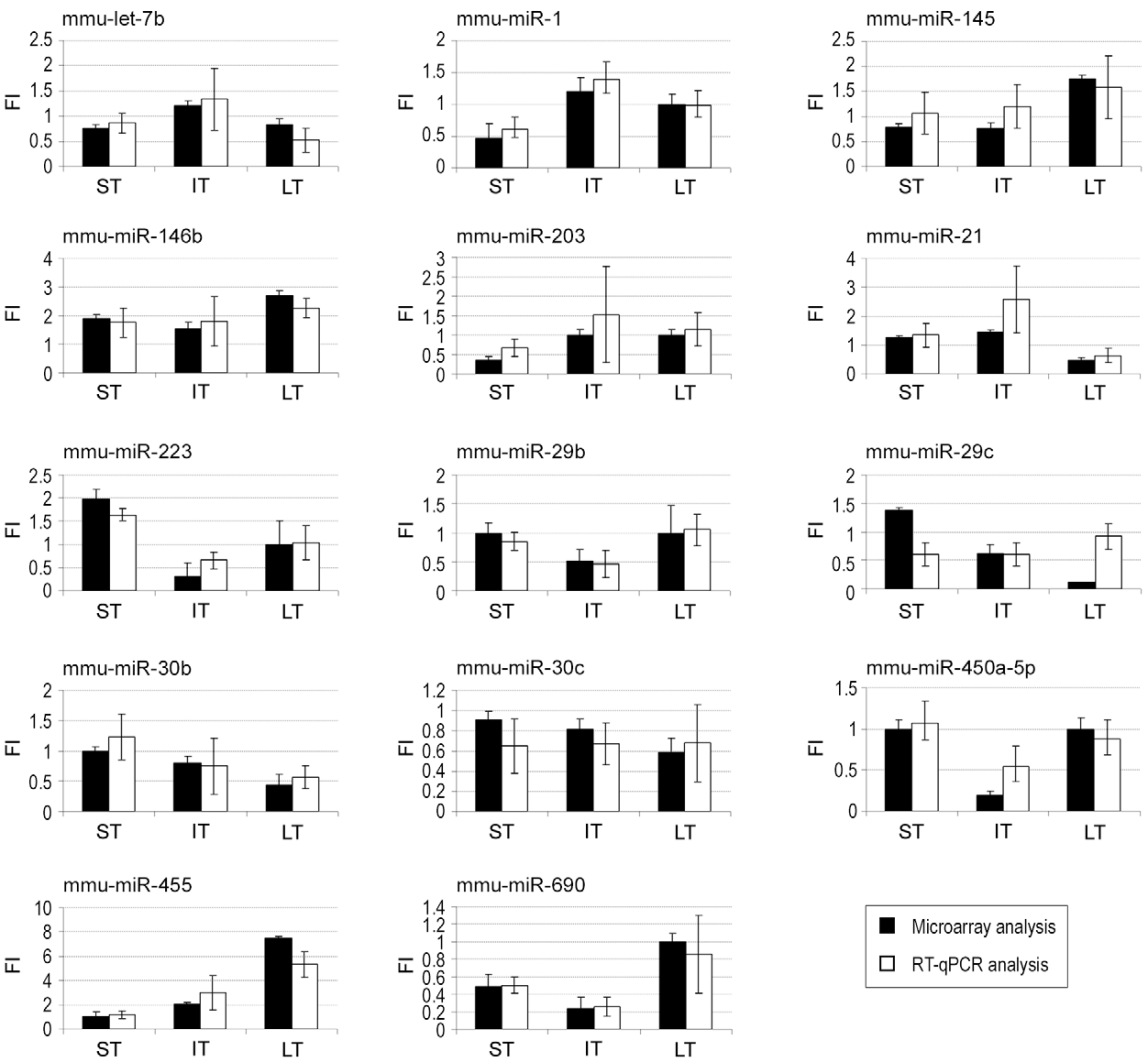
Validation of microarray data by real-time RT-qPCR. Comparison of miRNA level regulation as determined by microarray hybridization (several probes per target) performed on pooled total RNA and by RT-qPCR performed on total RNA of each individual mice. Results are expressed as means ± SD. The value “1” is arbitrarily given when no change is observed. ST, IT, LT: short, intermediate and long-term treatments, respectively. FI: fold induction.

Before considering further the potential implication of the regulation affecting the expression of several miRNAs, microarrays data, that were obtained by hybridization of RNA pools, were validated by RT-qPCR quantification for each individual mouse in the six experimental groups. Primers were designed for several miRNAs undergoing regulation at, at least, two time-points (such as mmu-miR-146b, -29c…) and for miRNAs selected on the basis of their abundancy (such as mmu-let-7b, mmu-miR-21, -145…), the magnitude of the observed regulations (such as mmu-miR-574-5p, -672…) and the potential significance of their mRNA targets (see below). In some cases, PCR amplifications were perturbed by several unspecific products preventing an accurate quantification. This pitfall was found to be related in part to the low level of some miRNAs and strongly related to the short size of mature miRNA preventing the design of highly specific primers. In order to overcome this technical problem, LNA-containing primers were designed to quantify these hard to amplify miRNAs (mmu-miR-146b, -29b and -29c). Quantitative RT-PCR was performed at the three time-points for 14 miRNAs, representing a total of 42 calculations of the OVA/PBS ratios based on individual mice measurements. Among these data, 40 out of 42 (95%) gave results and trends similar to those obtained by microarray profiling, except for mmu-miR-29c at ST and LT (Figure 2), although the magnitude of the observed regulation was not always identical (mmu-miR-455 at LT and mmu-miR-450a at IT). Besides problems related to PCR amplification as experienced with mmu-miR-29c, another potential explanation for the differences observed between the two quantification procedures could be related to the relative abundance of precursor miRNA (pre-miRNA) that are characterized by the persistence of a stem-loop structure. Depending upon their sequence and stability, these stem-loops are indeed expected to affect differently the efficiencies of the RT-PCR amplifications and the hybridization with probes immobilized on microarrays. As a result, differences in microarrays and RT-PCR data for a specific miRNA may be related to modifications of the level of its pre-miRNA, thus to the regulation of its transcription.

All together, these data demonstrate that microarray analysis is reliable and allows to detect changes in the level of expression of a large panel of miRNAs, providing a unique opportunity to investigate the role of miRNA-based RNA interference during the course of asthma.

### Several miRNAs were expected to participate in the regulation of biological processes during the course of asthma development

**Figure 3:**
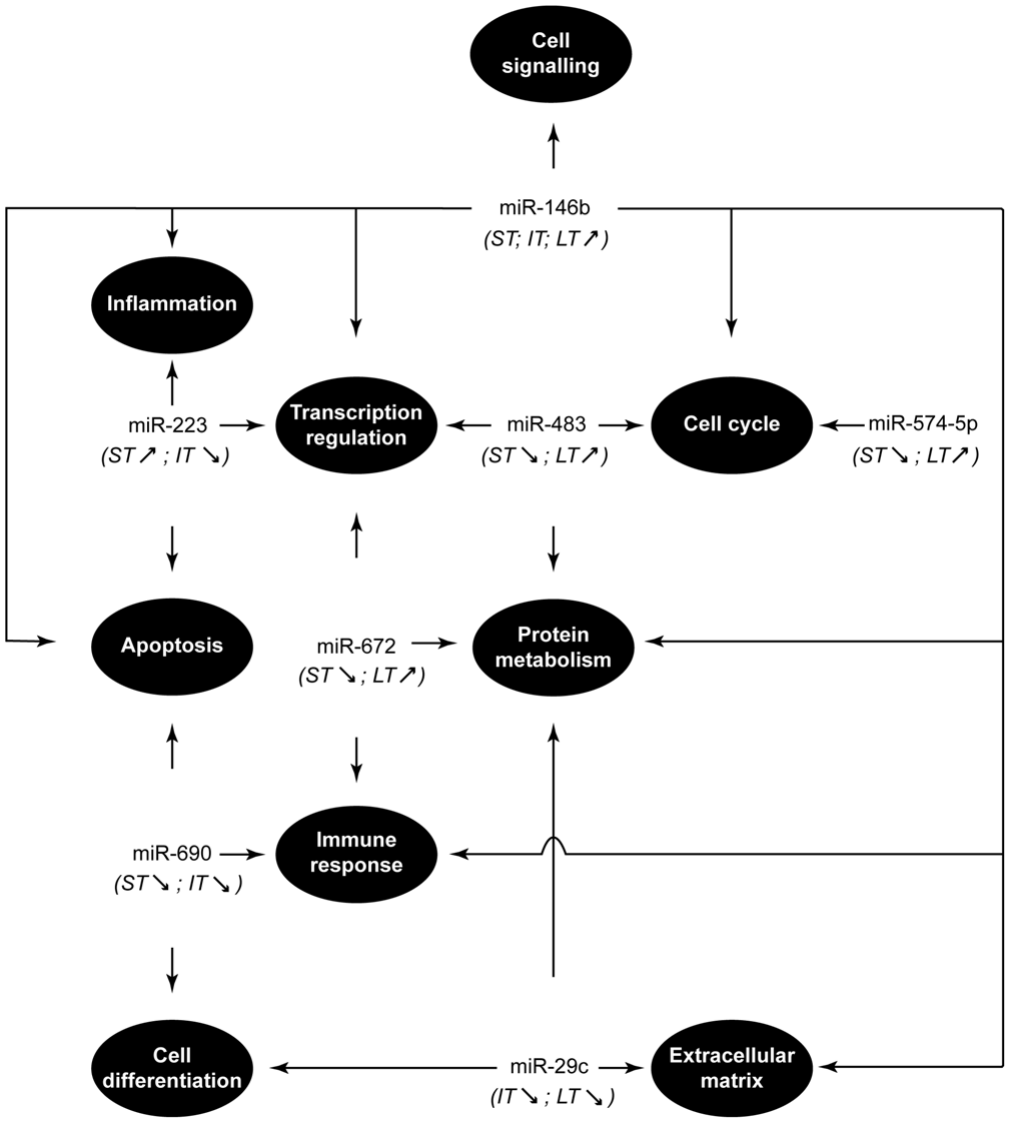
Potential regulation performed by miRNAs during the development of allergen-induced asthma. This *in silico* prediction is based on significant inverse correlation between mRNA and miRNA modulation of expression as detailed in tables 4 and 5. ST, IT, LT: short, intermediate and long-term treatments, respectively. The observed up- (<$>\raster="rg1"<$>) and down- (<$>\raster="rg2"<$>) regulations of the expression of the selected miRNAs are reported.

Knowing that miRNA can induce a significant degradation of its target and assuming also that evolution progressively selected inverse regulation of expression of mRNAs and their specific miRNAs, we determined (MicroCosm Target algorithm) for each miRNA its potential target(s) and the regulatory pathways that are expected to be regulated (Tables S2, S3 and S4). Among these results, we focused on predicted pairs of miRNAs and mRNAs that are inversely regulated at 2 time-points with a *p-value* <0.05 (Table 4). The potential targets of mmu-miR-146b, the only miRNA being significantly upregulated throughout the experiment, were also identified (Table 5). Collectively, these 7 selected miRNAs are expected to participate in the regulation of several biological processes involved in asthma as illustrated in Figure 3.

**Table 4:** Prediction of the mRNA targets for miRNA regulated at 2 time-points in the mouse model of asthma.

mmu-miRNA		Targeted mRNA		MicroCosm Targets	*A**ssociated** Biologicalprocess*
	FI		FI	*p-value*	
miR-223	1.99 ^ST^	ARID4B	0.64 ^ST^	0.0009	*Transcription regulation*
	0.32 ^IT^	ARID4B	19.70 ^IT^		
		CFLAR	0.66 ^ST^	0.0068	*Apoptosis*
		CFLAR	4.44 ^IT^		
		IL6	0.47 ^ST^	0.0110	*Inflammation*
		IL6	2.00 ^IT^		
		LPIN2	0.57 ^ST^	0.0050	*Transcription regulation*
		LPIN2	2.14 ^IT^		
miR-690	0.49 ^ST^	2010001M09RIK	6.06 ^ST^	0.0283	*Apoptosis*
	0.24 ^IT^	2010001M09RIK	2.00 ^IT^		
		CDCA8	3.03 ^ST^	0.0472	*Cell cycle*
		CDCA8	1.87 ^IT^		
		CTSE	1.57 ^ST^	0.0092	*Immune response*
		CTSE	2.73 ^IT^		
		FST	1.87 ^ST^	0.0008	*Cell differentiation*
		FST	2.64 ^IT^		
miR-29c	0.62 ^IT^	COL6A2	1.80 ^IT^	0.0000	*Extracellular matrix*
	0.11 ^LT^	COL6A2	1.87 ^LT^		
		CTSK	1.87 ^IT^	0.0007	*Protein metabolism*
		CTSK	1.80 ^LT^		
		METRNL	2.30 ^IT^	0.0041	*Cell differentiation*
		METRNL	1.62 ^LT^		
miR-483	0.39 ^ST^	GMNN	1.62 ^ST^	0.0455	*Transcription regulation*
	116.16 ^LT^	GMNN	0.55 ^LT^		
		MKI67	2.64 ^ST^	0.00147	*Cell division*
		MKI67	0.45 ^LT^		
		NOLA2	1.74 ^ST^	0.0300	*Ribosome biogenesis*
		NOLA2	0.55 ^LT^		
		UBE2C	2.46 ^ST^	0.0012	*Protein metabolism*
		UBE2C	0.39 ^LT^		
miR-574-5p	0.37 ^ST^	CCNB1	4.76 ^ST^	0.0339	*Cell cycle*
	13.18 ^LT^	CCNB1	0.10 ^LT^		
		CDCA8	3.03 ^ST^	0.0006	*Cell cycle*
		CDCA8	0.35 ^LT^		
		DERA	1.57 ^ST^	0.0058	*Nucleotide synthesis*
		DERA	0.66 ^LT^		
		NUSAP1	2.00 ^ST^	0.0477	*Cell cycle*
		NUSAP1	0.28 ^LT^		
miR-672	0.35 ^ST^	CD3G	1.74 ^ST^	0.0054	*Immune response*
	5.31 ^LT^	CD3G	0.32 ^LT^		
		PHB2	1.68 ^ST^	0.0042	*Transcription regulation*
		PHB2	0.66 ^LT^		
		PPP1R14B	1.74 ^ST^	0.0069	*Cell signalling*
		PPP1R14B	0.62 ^LT^		
		TOP2A	2.46 ^ST^	0.0037	*Transcription regulation*
		TOP2A	0.29 ^LT^		

The rationale for calculation (MicroCosm Target algorithm) is based on sequence complementarity between miRNA and the 3′UTR of its potential target, and on the inverse correlation of their regulation. The associated biological processes are also indicated.

ST, IT, LT : short, intermediate and long-term treatments, respectively. FI: fold induction.

**Table 5:** Predicted mRNA targets of mmu-miR-146b at the 3 time-points.

Target mRNA	FI	*ASSOCIATED BIOLOGICAL PROCESS*	MicroCosm Targets *p-value*
**ST (mmu-miR-146b FI = 1.91)**			
ALS2CL	0.59	*GTPase activator activity*	0.0035
AP4S1	0.57	*Vesicle mediated transport*	0.0028
APLP2	0.48	*ECM organization*	0.0097
CARD10	0.55	*Apoptosis/NF-κB activation*	0.0003
CFLAR	0.66	*Apoptosis/NF-κB activation*	0.0371
CLEC4D	0.39	*Immune response*	0.0016
GPR116	0.57	*Cell signalling*	0.0038
KLF13	0.66	*Transcription regulation*	0.0436
NCOA4	0.64	*Nuclear receptor activator*	0.0191
NUMB	0.62	*Cell signalling*	0.0089
RASIP1	0.66	*Angiogenesis*	0.0000
SCUBE2	0.52	*Inflammation*	0.0002
TCFCP2L1	0.57	*Transcription regulation*	0.0391
TNFSF9	0.59	*Immune response*	0.0021
UBR1	0.66	*Protein metabolism*	0.0001
UTRN	0.64	*Cell signalling*	0.0095
ZFP451	0.57	*Transcription regulation*	0.0003
**IT (mmu-miR-146b FI = 1.56)**			
BAIAP2L1	0.66	*Cell signalling*	0.0004
FOXP4	0.52	*Transcription regulation*	0.0134
HIST2H3C2	0.52	*Nucleosome assembly*	0.0371
PKP2	0.62	*Cell interaction*	0.0010
ZNHIT1	0.64	*Unknown*	0.0084
**LT (mmu-miR-146b FI = 2.69)**			
BTF3	0.64	*Transcription regulation*	0.0367
CDCA3	0.44	*Cell cycle*	0.0300
DTYMK	0.62	*Nucleotide biosynthesis*	0.0052
HMGCS1	0.66	*Lipidic metabolism*	0.0069
HORMAD1	0.47	*Cell cycle*	0.0046
KIF22	0.29	*Microtubules*	0.0036
MGEA6	0.55	*Unknown*	0.0230
RPS9	0.62	*Protein metabolism*	0.0001
UBE2D2	0.62	*Protein metabolism*	0.0418
WDR12	0.59	*Cell signalling*	0.0261

Mmu-miR-146b is upregulated and predicted targeted mRNA are downregulated at each time-point. ST, IT, LT: short, intermediate and long-term treatments, respectively. FI: fold induction.

#### Functional correlation between the expression of miRNAs and some of their potential targets

**Figure 4:**
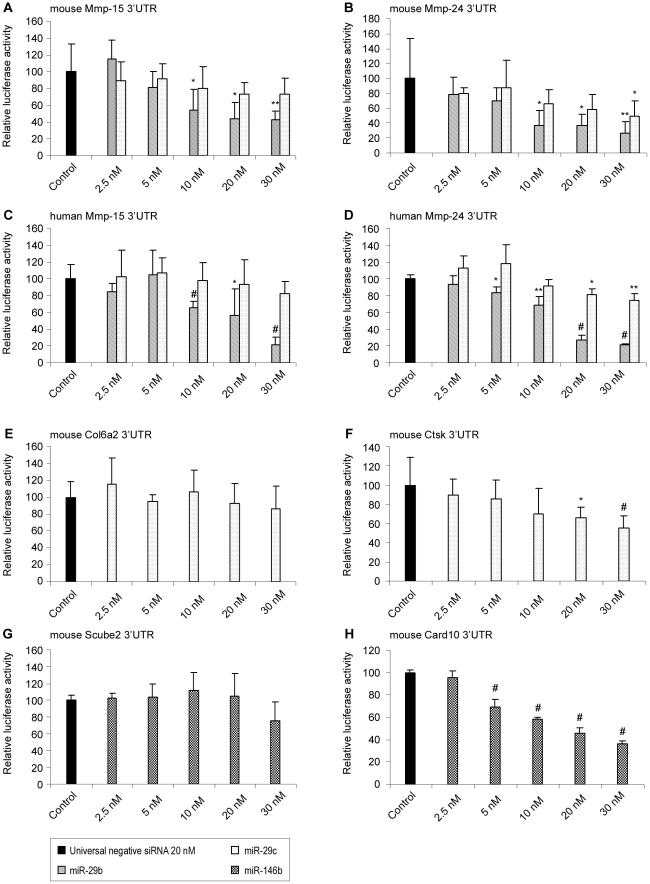
Dose-response analysis of the effect of miR-29b, -29c and -146b on their predicted target in lung cells. Transient transfection analysis for luciferase reporter expression with mouse Mmp-15 3′UTR in the presence of miR-29b and -29c (Panel A); mouse Mmp-24 3′UTR in the presence and absence of miR-29b and -29c (Panel B); human Mmp-15 3′UTR in the presence of miR-29b and -29c (Panel C); human Mmp-24 3′UTR in the presence of miR-29b and -29c (Panel D); mouse Col6a2 3′UTR in the presence of miR-29c (Panel E); mouse Ctsk 3′UTR in the presence of miR-29c (Panel F); mouse Scube2 3′UTR in the presence of miR-146b (Panel G); mouse Card10 3′UTR in the presence of miR-146b (Panel H). Universal negative siRNA was used at 20 nM as non-functional small RNA control. For each expression vector, the specific effect of the miRNA on luciferase activity was expressed as compared to the activity measured in the control condition, arbitrarily set at “100”. Results are expressed as mean ± SD. (* *p-value* <0.05; ** *p-value* <0.001; # *p-value* <0.005).

**Figure 5:**
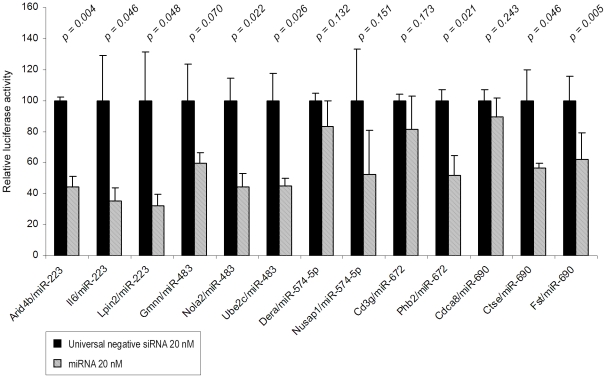
Analysis of 13 miRNAs-predicted target murine genes *in vitro*. Transient transfection analysis for luciferase reporter expression with Arid4b, Il-6 or Lpin2 3′UTR in the presence of miR-223; with Gmnn, Nola2 or Ube2c 3′UTR in the presence of miR-483; with Dera or Nusap1 3′UTR in the presence of miR-574-5p; with Cd3g or Phb2 3′UTR in the presence of miR-672; and with Fst, Ctse or Cdca8 3′UTR in the presence of miR-690. Universal negative siRNA were used at 20 nM as non-functional small RNA control. For each expression vector, the specific effect of the miRNA on luciferase activity was expressed as compared to the activity measured in the control condition, arbitrarily set at “100”. Results are expressed as mean ± SD. Each *p-value* is indicated in the graph.

As shown in Tables 4 and 5, modulation of some miRNAs could be involved in the pathogenesis of asthma by regulating genes at acute and/or chronic stages of the disease. We selected 8 miRNAs (those described in Tables 4 and 5 and mmu-miR-29b) and, at least, 2 of their potential targets for functional testing *in vitro* (Figures 4 and 5).

In this assay, cells from lung origin were cotransfected with synthetic miRNA mimic or non-functional small RNA control and expression vectors containing the coding sequence of luciferase cloned upstream of either an irrelevant control 3′UTR or the 3′UTR of the respective potential mRNA target. In this experimental set up, the efficiency of the miRNA-dependent regulation was directly proportional to the decrease in luciferase activity used as a reporter.

For miR-146b, which was up-regulated at the 3 time-points, and for miR-29b and -29c, which are thought to be regulators of extracellular matrix remodelling and are downregulates at IT and LT, increasing concentrations were tested to evaluate the sensitivity and the specificity of the assays (Figure 4).

Despite their close sequence similarity, miR-29 members showed different inhibitory patterns. MiR-29b mimic reduced efficiently and dose-dependently the luciferase activity from constructs containing the 3′UTRs of mouse Mmp-15 and Mmp-24 while miR-29c had only a limited effect at high concentration on Mmp-24 3′UTR (Figure 4A, B). Since the sequence of miR-29b and -29c are identical in human and mouse, their efficiencies were also evaluated using the 3′UTRs of human Mmp-15 and Mmp-24. Similar regulations were observed using human and mouse 3′UTRs, which confirm both the specificity of the inhibitions and the relevance of our findings in the context of human asthma.

Among the 17 others miRNA-mRNA pairs that were evaluated (Figure 4E-H and Figure 5), significant inhibition was observed in 10 experimental conditions (miR-29c and Ctsk; miR-146b and Scube2; miR-483 and Nola2 or Ube2c; miR-672 and Phb2; miR-223 and Il6 or Lpin2 or Arid4b; miR-690 Fst or Ctse). All together these validation analyses indicate that 64% (16/25) of the *in silico* predicted regulations are effective while inhibitions were also observed in the 9 remaining cases but were too low to reach statistical significance. Although optimization of the experimental design (timing for sample collection, transfection conditions, target concentration…) would have probably led to the confirmation of other miRNA-mRNA regulatory pairs, these experiments were not further continued since the aim of these functional assays was only to validate the *in silico* predictions before addressing the potential roles of miRNAs during asthma.

#### Identification of biological processes regulated by miRNAs

Genes involved in transcriptional regulation are modulated at each time-point in our experimental model [4]. Four of selected miRNAs (Tables 4 and 5) displayed a clear correlation with this biological process at 2 or 3 time-points. The expression of mmu-miR-483 and -672 was downregulated at ST as compared to the PBS-treated mice while the levels of mmu-miR-223 and -146b were increased. Their putative targets in this biological process embrace direct transcription initiator (TOP2A, topoisomerase II α), transcription factors (ZFP451, BTF3, KLF13 and TCFCP2L1), transcription coactivators (LPIN2) or corepressors (FOXP4, GMNN, PHB2) and proteins playing roles in functional organization of chromosome structure through chromatin remodeling (ARID4B).

We also observed that the mRNA potentially targeted by mmu-miR-223 (Arid4b, Lpin2, see Figure 5) and mmu-miR-146b (Zfp451, Klf13 and Tcfcp2l1) did undergo a downregulation at ST. This effect could be reinforced by the downregulation of mmu-miR-672, which potentially targets PHB2, a factor that restrains estrogen action and its activating pathway [65] and by repression of mmu-miR-483 that could be responsible for the observed upregulation of GMNN, an inhibitor of HOX-dependent transcriptional activity [66]. However, topoisomerase II α (Top2a) mRNA, which appears to be inversely correlated to mmu-miR-672 expression, was significantly upregulated at ST and downregulated at LT. An inversion in the pattern of transcription-related mRNA-miRNA modulations was shown at LT exposure to allergens. From these findings, it can be speculated that the potential role of miRNAs in the modulation of transcription mechanisms would mostly consist in a fine-tuning process rather than striking regulations.

#### Cell cycle

MiRNA targeting genes regulating cell cycle are clearly downregulated at ST, not affected at IT and upregulated at LT. GMNN, which negatively regulates cell cycle, and MKI67, which is an endogenous marker of proliferative cells, are putative targets of mmu-miR-483. The expression of CCNB1 (cyclin B1) that activates CDK1 driving G2/M-phase progression [67], of CDCA8 (borealin) that is required for the proper segregation of chromosomes during mitosis [68] and of NUSAP1 that is selectively expressed in proliferative cells and is a positive regulator of mitosis by acting on microtubules organization [69] were significantly inversely correlated to mmu-miR-574-5p expression. These correlations reinforce the hypothesis that cell proliferation predominates at the early stages of asthma development rather than in the late stage and might probably partly be under miRNA control, especially through mmu-miR-574-5p regulation.

#### Protein metabolism

Inverse correlations were also observed between miRNAs and genes involved in protein metabolism. As discussed further in the Inflammatory Response Pathway section, mmu-miR-29 appears to be a miRNA family displaying a protective role against fibrosis. Mmu-miR-29c decrease could lead to the upregulation of CTSK (cathepsin K, see Figure 4F) at the intermediate and the late stages of asthma progression. CTSK is involved in lung matrix homeostasis by degrading extracellular matrix proteins before their secretion, therefore preventing excessive matrix deposition [70].

Ubiquitination is a step leading to protein degradation by the proteasome. Transcriptional downregulation was observed for some factors contributing to this process (UBE2C, UBE2D2 and UBR1), especially at the LT time-point. A statistically significant inverse correlation has been observed with increased mmu-miR-146b and -483 expressions. According to our data, protein production is repressed during the first phases of asthma development and then increases concomitantly with tissue remodeling at the late stages of the disease. These modulations could result from the simultaneous regulation of the levels mmu-miR-483 (see Figure 5), -672 and -146b.

#### Apoptosis, immunity, inflammation and cell signalling

*In silico* analysis have also pointed significant correlations between miRNA (mmu-miR-146b, -223 and -690) and modulations of mRNAs related to apoptosis processes. Induction of apoptosis in inflammatory cells, including eosinophils, might have a beneficial effect on airway hyperresponsiveness observed at ST [71] while its inhibition at IT might lead to the development of allergic disease [72]. Regulation of apoptosis by miRNAs is expected to occur via NF-κB pathway by inducing the degradation or inhibiting the translation of apoptosis-related mRNA as already described for mmu-miR-146b [63]. In our model, CFLAR, an NF-κB-inducible anti-apoptotic protein which inhibits caspase 8-mediated apoptosis, is a putative target of mmu-miR-146b and -223. The upregulation of these two miRNAs at ST should therefore induce apoptotic mechanisms in inflammatory cells. This hypothesis is further reinforced by the demonstration that mmu-miR-146b inhibits the expression of CARD10 (see Figure 4H), a molecular scaffold for the assembly of a BCL10 signalling complex that activates NF-κB [73].

MiR-146 is also associated with inflammation and innate immune responses where it regulates the response to a variety of microbial components and proinflammatory cytokines [63]. In our ST protocol, upregulation of mmu-miR-146b and -223 is predicted to repress SCUBE2 and IL-6, factors that are under the control of IL-1β and TNF-α [74], [75], probably through NF-κB activation.

**Figure 6:**
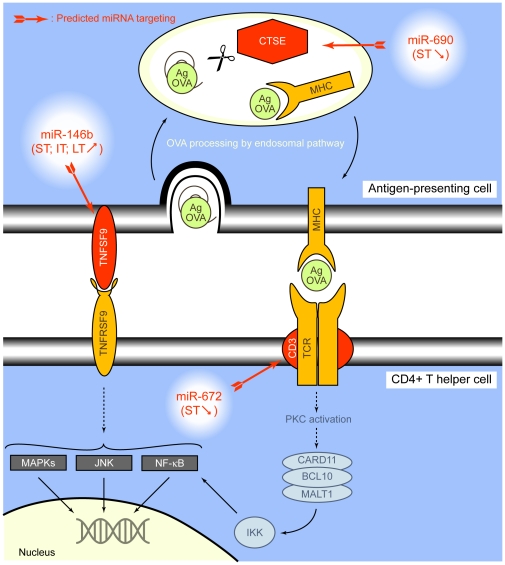
Potential influence of miRNAs on immune response induced by OVA. In antigen presenting cells, MHC complexes are maturated in endosomes by lysosomal reductases while CTSE processes antigen (Ag), i.e. OVA. Finally, the MHC/antigenic peptide complex translocates to the plasma membrane and is presented to the TCR/CD3 complex on CD4+ T helper cell surface. Activation of TCR/CD3 induces the activation of NF-κB through PKC activation and CARD11/BCL10/MALT1 complex recruitment. MiRNA modulation could occur through mmu-miR-690, -672 and -146b. Cross-linking of costimulatory receptors on the T helper cell with corresponding ligands, such as TNFRSF9 with TNFSF9, also induces NF-κB and regulators (MAPKs, JNK) of the activity of multiple transcription factors. Production of TNFSF9 could be under the control of mmu-miR-146b, thus regulating the T helper cells properties. ST, IT, LT: short, intermediate and long-term treatments, respectively. The observed up- (<$>\raster="rg1"<$>) and down- (<$>\raster="rg2"<$>) regulations of the expression of some specific miRNAs are reported.

The immune response, clearly stimulated by OVA in the early phase of asthma development [4], is mediated by CD4^+^ T helper cells, eosinophils, neutrophils, macrophages, and IgE antibodies. The increased expression of CTSE (cathepsin E) at ST [4] stimulates the generation of antigenic epitopes from OVA (Figure 6) [76]. Similarly, an overexpression of CD3G, a protein forming a complex with TCR, favour the activation process. Signalling upon TCR stimulated by MHC presented antigen induces IL-13 production, antigen-specific Th2 response and expression of anti-apoptotic genes through NF-κB translocation [77]. A co-stimulatory pathway of regulation involves the cross-link of receptors on T cells with their corresponding ligand(s), such as TNFRSF9 with TNFSF9 (Figure 6). These different pathways are under direct control of several miRNAs. Mmu-miR-690 and-672, that were repressed at ST, are potential inhibitors of CTSE and CD3G production meaning that their downregulation could increase the activation of T cells. Similarly, the continuous overexpression of mmu-miR-146b should reduce the synthesis of CLEC4D and TNFSF9. The stimulation of TNFRSF9 *in vivo* has been shown to inhibit allergic asthma by decreasing IgE production [78] and regulation of the B cell response [79], [80] and to fine-tune the Th1/Th2 balance [77]. The inhibition of TNFSF9 by mmu-miR-146b could therefore play a central function in the evolution of the disease.

Another aspect of cell function appeared to be modulated by mmu-miR-146b. NUMB expression is inhibited by miR-146a in various cell lines [81]. NOTCH and NUMB controls the proliferative/differentiation balance in development and homeostasis. An increased degradation of NUMB, that antagonizes the cell surface receptor NOTCH, leads to uncontrolled cell proliferation in human mammary gland tumors [82]. Hyperplasia is also a characteristic of remodelling of airway walls and concerns epithelial cells, smooth muscle cells, fibroblasts and goblet cells. A decrease in NUMB at ST should result in substantial increase of cell proliferation. In the LT protocol, a downregulation of WDR12, another putative target of mmu-miR-146b, was also observed. This factor is involved in differentiation processes related to NOTCH signalling [83]. Therefore, mmu-miR-146b probably controls some aspect of cell proliferation and may participate in the development of subepithelial fibrosis in asthma, by regulating NUMB and WDR12 expression in the NOTCH signalling pathway.

### MiRNAs modulation in different mice models of asthma was significantly associated with four signalling pathways

A large scale unbiased approach to determine the significance of the observed regulations of miRNA expression was also used. At each time-point, a set of predicted mRNA targets was identified for each miRNA significantly up- or downregulated above 1.5 fold. The use of a cut-off threshold of 1.5 on the fold-change is not uncommon in the context of miRNA profiling studies [84]-[86]. Since a single miRNA has several targets, moderate modifications of its expression may have deep impact at a cellular level. Moreover for studies profiling miRNA in complex tissues, such as lung in this study, a cut-off of 1.5 is more appropriate to identify regulations affecting only subpopulation of cells and not the entire organ. Based on the known functions of the protein encoded by these mRNA, pathways of regulation affecting behaviour and partly controlled by miRNAs can be identified. In order to further strengthen the analysis, two different databases were used. Target prediction by MicroCosm Targets database is performed on optimal sequence complementarity between a set of mature miRNAs and a given mRNA using an algorithm reflecting the weight of each base position and allowing mismatches at the 5′ end of miRNA [87]. Target prediction by TargetScan database is established on sequence complementarity to target sites with emphasis on perfect base-pairing in the seed region and on sequence conservation among species [88].

**Figure 7:**
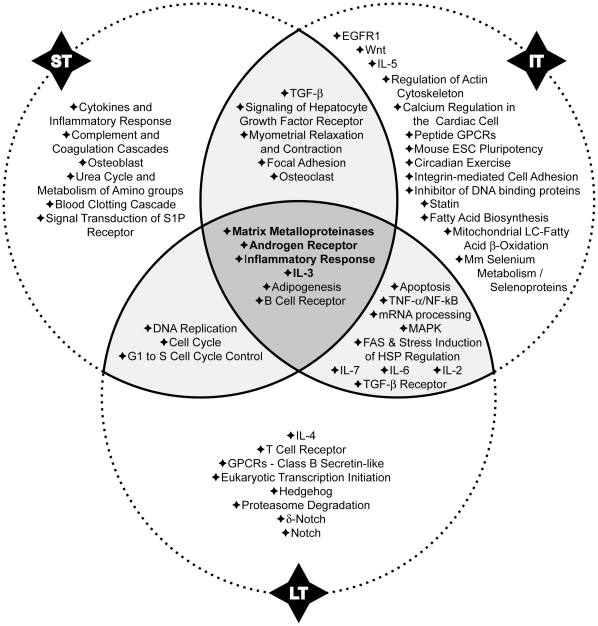
Regulatory pathways regulated by miRNAs as determined by the MicroCosm Targets algorithm. Fifty-one pathways were identified at one time-point at least. While 28 pathways appeared to be modulated at only one stage of the disease (ST, IT or LT), 17 were regulated at 2 different time-points and 6 during the entire course of the disease. Stouffer's method was used to identify significant enrichment for pathways annotations among predicted targets of modulated miRNA in the model. ST, IT, LT: short, intermediate and long-term treatments, respectively.

**Figure 8:**
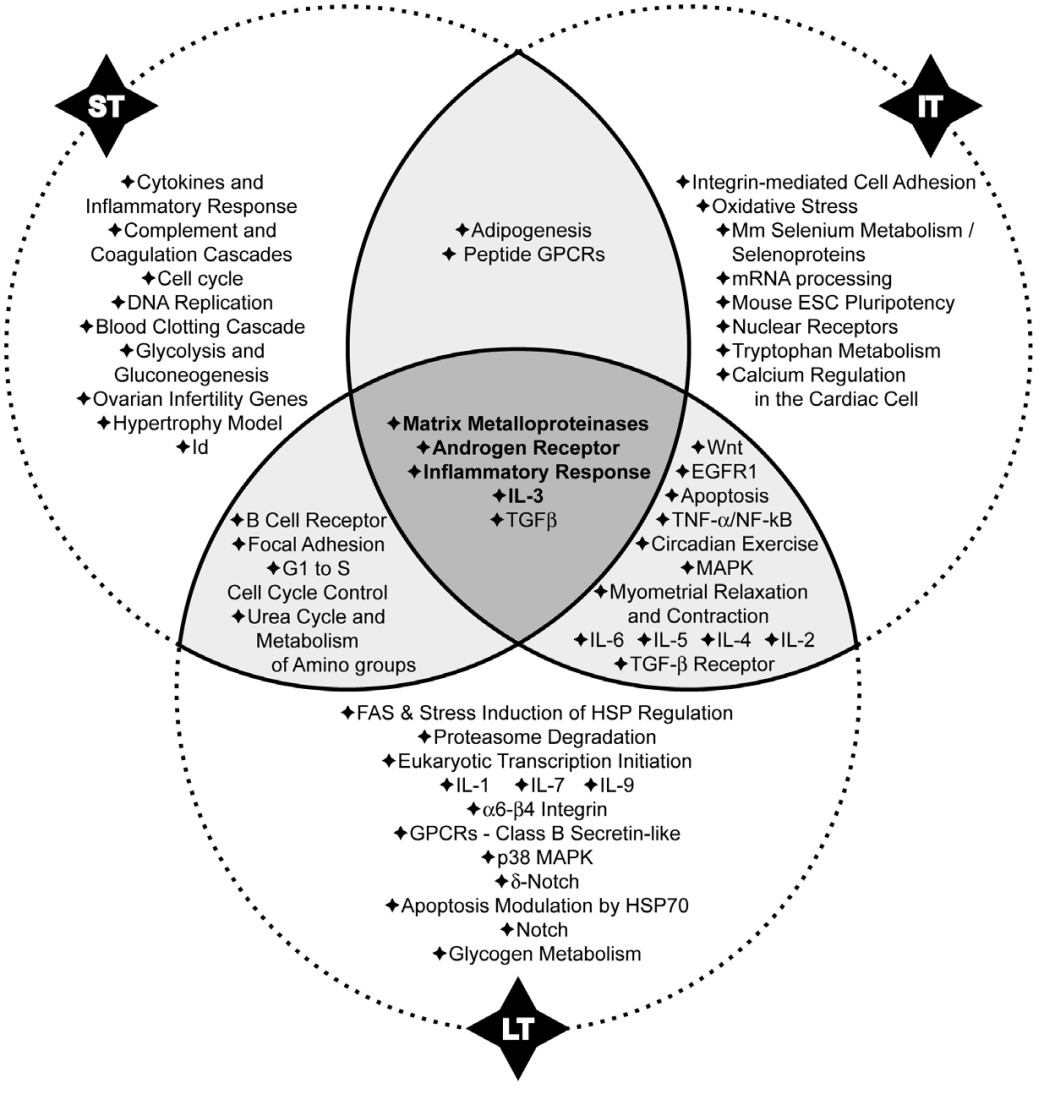
Regulatory pathways regulated by miRNAs as determined by the TargetScan algorithm. Fifty-three pathways were identified at one time-point at least. While 30 pathways appeared to be modulated at only one stage of the disease (ST, IT or LT), 18 were regulated at 2 different time-points and 5 during the entire study. Stouffer's method was used to identify significant enrichment for pathways annotations among predicted targets of modulated miRNA in the model. ST, IT, LT: short, intermediate and long-term treatments, respectively.

Sixteen pathways at ST, 25 at IT and 19 at LT were selected with a combined *p-value* <0.05 (Tables S5, S6 and S7) with MicroCosm Targets database and 16 pathways at ST, 21 at IT and 32 at LT were selected with a combined *p-value* <0.05 (Tables S8, S9 and S10) with TargetScan database. The comparison of these data highlighted four common pathways that are modulated at the three time-points (Figures 7 and 8). Matching results from the two algorithms allowed us to select signalling pathways strongly involved in the development of asthma. Some interactions between modulated miRNAs and their potential targets are discussed below.

#### Matrix Metalloproteinases Pathway

Matrix Metalloproteinases (MMPs) Pathway (WP441) was the most significantly modified by the miRNA modulation at the three time-points (*p-value* ≤0.01), which confirms our previous transcriptomic analysis [4]. MMPs and their inhibitors (tissue inhibitors of matrix metalloproteinases, TIMPs) contribute to the pathogenesis of asthma by regulating the cleavage of peptidic mediators that influence the functions and the migration of inflammatory cells and by modifying the extracellular matrix homeostasis. Apart from their role in acute airway inflammation, MMPs may contribute to features of airway remodelling including reorganization of matrix, angiogenesis and smooth muscle hyperplasia.

**Figure 9:**
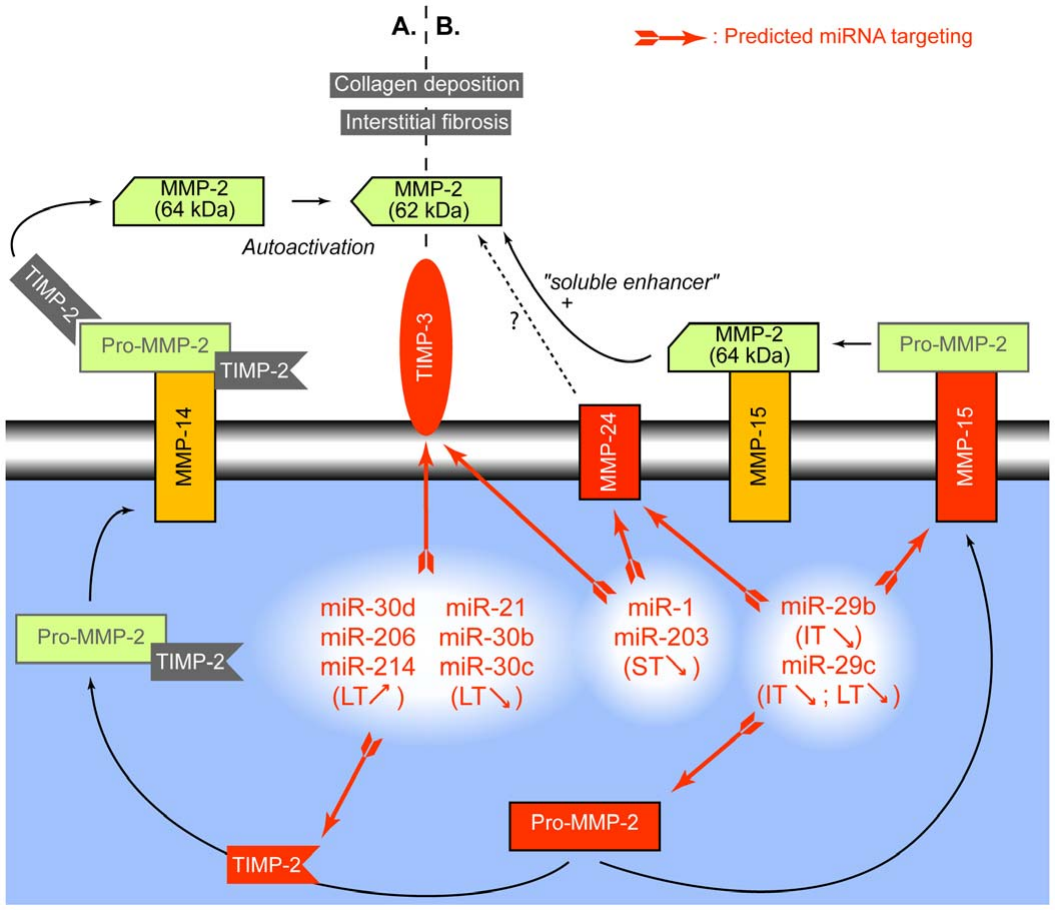
miRNAs and MMP-2 activation. Panel A represents MMP-14 (MT1-MMP)-dependent activation pathway for MMP-2. TIMP-2 activates pro-MMP-2 by forming a complex that interacts with the MMP-14/TIMP-2 complex at the cell membrane. Activation of pro-MMP-2 occurs in a two step process: cleavage within the MMP-2 prodomain followed by an autocatalytic cleavage which results in the active 62 kD form. Panel B represents the presumed MMP-15 (MT2-MMP) and MMP-24 (MT5-MMP)-dependent activation pathway for MMP-2. Activation of pro-MMP-2 occurs in a two step process: cleavage within the MMP-2 prodomain in the absence of TIMP-2 followed by a second cleavage, enhanced by an unidentified secreted soluble protein which results in the active 62 kD form. The mechanism by which MMP-24 releases active MMP-2 is currently unknown. The intensive activation of MMP-2 contributes to collagen deposition and interstitial fibrosis. An excess of TIMP-2 and the extracellular matrix-anchored TIMP-3 contribute, respectively, to the degradation of pro-MMP-2 and to the inhibition of MMP-2. ST, IT, LT: short, intermediate and long-term treatments, respectively. The observed up- (<$>\raster="rg1"<$>) and down- (<$>\raster="rg2"<$>) regulations of the expression of some specific miRNA are reported.

At ST, a decrease in mmu-miR-203 and -1 expression is expected to induce the upregulation of MMP-24 and TIMP-3 (Figure 9). At IT, repression of mmu-miR-29b and -29c should induce an increase expression of MMP-15 and -24 (Figure 4A-D) but also of MMP-2. Moreover, these miRNAs can prospectively target MMP-2 and MMP-15. Finally, at LT, TIMP-2 and TIMP-3 mRNAs are the predicted targets of several modulated miRNAs (mmu-miR-30b, -30c, -30d, -21, -214 and -206).

Deregulation of TIMPs and MMPs activities can lead to an exaggerated matrix turnover and be responsible for subepithelial fibrosis observed in asthma. A common feature of MMP-15 and MMP-24 is that they are membrane-type MMPs sharing an identical substrate, the pro-MMP-2. In lung fibrosis, in the late stages of the disease, MMP-2 may be associated to pathological collagen deposition and interstitial fibrosis [89]. Since no significant modifications in MMP-2, MMP-15 and MMP-24 mRNA levels were observed [4], it strongly suggests that miRNAs regulate the translation of these MMPs [11]. This hypothesis is further reinforced by our functional data showing that 3′UTRs of both human and mouse Mmp-15 and -24 are similarly targeted by miR-29b and -29c. Finally these results are in line with those of Henderson *et al.* [90] describing post-transcriptional regulation of MMP-2 and TIMP-2 protein levels and demonstrating a sharp increase in MMP-2 activation during airways remodelling in asthma.

TIMPs are key regulators of the activity of many metalloproteinases including MMPs, ADAMs and ADAMTS. TIMP-1 mRNA upregulation during lung fibrosis has already been described as TIMP-2 and TIMP-3 mRNA were shown to be constitutively expressed [4], [89]. TIMP-2 not only inhibits MMP-2 activity but is also involved in docking pro-MMP-2 to the cell surface where the enzyme is activated [91] by membrane-bound MMPs, including MMP-15 and MMP-24 [92], and by a second molecule of TIMP-2. TIMP-3 binds to the extracellular matrix and may be important in allowing excess matrix accumulation in asthmatic airways [93]. At LT, 6 different miRNAs potentially targeting TIMP-2 and TIMP-3 are significantly up or downregulated which is highly susceptible to affect the complex array of interactions between MMPs and between MMPs and their targets (extracellular matrix macromolecules, cytokines…) and may represent a crucial regulatory switch during asthma disease.

#### Androgen Receptor Signalling Pathway

Several miRNAs regulated in asthma target mRNAs that are implicated in the Androgen Receptor Signalling Pathway (WP252). Although it could seem surprising, cross-talk between this pathway and regulatory cascades originating from growth factors (IGF1, FGF2, EGF, TGFβ) are well documented. In prostate and lung, they have been shown to regulate several cell functions with direct implication in chronic asthma (such as apoptosis, survival, proliferation and differentiation [94], [95]).

IGF1 is known to increase fibroblast survival and growth, and to be an important mediator of inflammation and remodelling in the asthmatic airways, as well as an inducer of bronchial smooth muscle contraction [96], [97]. We had previously shown that IGF1 mRNA is strongly upregulated at ST, IT and LT [4]. This may be due to transcriptional regulation [98] but possibly also to the repression of miRNAs (mmu-miR-1 at ST; mmu-miR-450a-5p at IT; mmu-miR-27a and -92a at LT) that target IGF1 mRNA. Constant upregulation of mmu-miR-146b could also influence changes that occur in the complex IGF1-dependent regulatory cascades.

In this study, we also underlined the importance of interactions between modulated miRNAs during mouse model of asthma and transcription factors (SP1, POU2F2, PATZ1…), receptors (NRIP, NR3C1…), nuclear receptor co-factors (NCOR2, NCOA1, NCOA3, PIAS1, PIAS3, NROB1, PNRC1…) or signal transducers (IL6ST, RAN…) participating in the Androgen Receptor Signalling Pathway. These evidences support the hypothesis that, besides transcription factors only, miRNA or miRNA/transcription factors networks participate in the control of this pathway and of the release of IGF1.

#### Inflammatory Response Pathway

Recent reviews [99], [100] have shown evidence that miRNAs play a role in the control of inflammation cascades and particularly in cytokines regulation. Tumour necrosis factor-alpha (TNFα) promotes inflammation and airway remodelling. It is intensely released in asthmatic airways by pro-inflammatory cells, including activated macrophages, but also by structural cells [101]. Its elevated levels in asthmatic patients seem mainly due to a massive release of the preformed cytokine [102] rather than to an increased transcription. No correlation between miRNA modulation and TNFα was made in this study. By contrast, the expression of its two main receptors, TNFRSF1A (TNF-R1) and TNFRSF1B (TNF-R2), is expected to be upregulated at ST and IT as a result of the downregulation of mmu-miR-690, -805 and -574-5p (at ST) and mmu-miR-29b, -29c, -152, -218 and 690 (at IT). This would induce an enhanced receptor production and accumulation at cell surface, and could contribute to an amplification of this regulatory cascade, in synergy with the massive release of TNFα. At LT, mmu-miR-29c and -98 are downregulated but upregulation of mmu-miR-125b-5p and -574-5p, and progressive normalization of the levels of mmu-miR-218, -690 and -805 would then be part of the reduction of the inflammatory process at the late stage of the asthma model through the modulation of TNFα receptors.

Downregulation of mmu-miR-29 members is strongly correlated with Inflammatory Response Pathway (WP458) especially with the extracellular matrix components directly involved in fibrosing processes. These miRNAs are speculated to regulate the expression of type I and type III collagens but also the laminin γ chain LAMC1. Evidence that miR-29b attenuates expression of collagen genes by blocking their mRNA translation has already been described [103], [104] and an inverse correlation between the expression of mmu-miR-29b and -29c and the synthesis of collagen type I and III is further evidenced here. This suggests that downregulation of mmu-miR-29 members coud be one of the causes of the subepithelial fibrosis observed in chronic asthma.

Fibronectin (FN1) is present in large quantities in fibrosing areas [105], precedes collagen type I deposition and is necessary for pulmonary fibrosis to develop [106]. Surprisingly however, its mRNA is only slightly upregulated at IT and not at ST or LT [4]. This apparent discrepancy can probably be explained by the decrease of the post-transcriptional repression by miRNAs at each step of the progression of the disease [107]-[109]. Here we showed that several modulated miRNAs could potentially target Fn1 mRNA at the three time-points. All of them were downregulated: mmu-miR-1 and -805 at ST, mmu-miR-199a-3p, -200a and -429 at IT and mmu-miR-27a and -200b at LT, except mmu-miR-206 which was upregulated at LT.

Together with collagens and fibronectin, laminin is another key component of the extracellular matrix that is exceedingly accumulated in airway walls during remodelling processes in patients with severe bronchial asthma [110]. In this mouse model of asthma, various laminin chains (LAMA5, LAMB1, LAMC1 and LAMC2) expressions were predicted to be affected by many miRNAs. However, there is no clear tendency at ST and LT where targeting miRNAs are up- or downregulated in equal proportions. At IT, expressions of mmu-miR-29b, -29c, -152, -200a and -690 that potentially target laminin γ chain LAMC1 are inhibited.

The miRNAs implication in the regulation of the Inflammatory Response Pathway appears to occur principally through the regulation of extracellular matrix component expression rather than through the modulation of cytokines and their receptors, except for the TNF-α receptors.

#### IL-3 Signalling Pathway

*In silico* analysis predicted IL-3 Signalling Pathway (WP373) as regulated by miRNAs. Eosinophils activated by IL-3 contribute to T cell activation in allergic diseases [111] and play a critical role in the induction of airway hyperreactivity and the development of lesions that underpin chronic airway wall remodelling [112].

IL-3 induces transient association between paxillin (PXN) and vinculin (VCL), two cytoskeletal proteins necessary for the contractile response of smooth muscle cells as observed during asthma [113]. During the whole time course of the model, PXN is a potential target of several downregulated (mmu-miR-203, -218, -30b, -30c, -27a and -21) and upregulated (mmu-miR-20b, -466g, -30d and -145) miRNAs. At IT and LT, VCL is a potential target of 5 downregulated miRNAs (mmu-miR-21, -25, -29b, -29c and -92a). Besides VCL binding, PXN interacts with SRC and CRK family members [114], which leads to proliferation and differentiation processes in fibroblasts through the MAPK pathway or regulates the formation of physical links between the cytoskeleton and integrin proteins that mediate transmission of contraction forces [115]. It is of interest that a large number of modulated miRNAs were pointed out to interact with SRC (mmu-miR-197 and -230), CRK and CRKL (mmu-miR-203, -497, -218, -320, -214, -328), and various MAPKs (4 miRNAs for MAPK1, 3, 8 and 14; 7 miRNAs for MAPK9; 13 miRNAs for MAPK7; see Tables S5-S10 for details).

IL-3 induces Toll-like receptors expression in dendritic cells [116] that then initiate the activation of T lymphocytes for specific antigens [117]. Genes implicated in Toll-like receptors signaling such as GSK3B (a glycogen synthase kinase), SOCS3 (a suppressor of cytokine signalling-3) and ATF2 (an activating transcription factor) were defined as targets of modulated miRNAs during all time-course of the mouse model of asthma, respectively mmu-miR-23a, -23b, -26b, -29b, -29c, -155 and -214 for Gsk3b; mmu-miR30b, -30c, -30d, -152, -203, -207, -218 and -455 for Atf2; mmu-miR30b, -30c, -30d, -152, -203, -207, -218 and -455 for Socs3.

YWHA proteins (tyrosine 3-monooxygenase/tryptophan 5-monooxygenase activation protein) act as adaptor proteins in cellular signaling and metabolism by regulating and coordinating a diverse array of cellular processes, such as cell cycle progression, apoptosis, protein trafficking, cytoskeleton rearrangements, metabolism, and transcriptional regulation of gene expression [118]. They can bind to GSK3 [119], implicated in Toll-like receptors signalling. For the three time-points, 23 modulated miRNAs (see Tables S5-S10 for details) could target mRNAs of three members of YWHA protein family: Ywhab, Ywhaq and Ywhaz. To our knowledge, the role of these proteins in asthma is not known.

The whole analysis for each time-point, including details on number of miRNA and miRNA-mRNA interactions in specific pathways is available in online supplemental data.

In conclusion, this work represents the first large scale study aiming at evaluating the implication of miRNAs during asthma, from early inflammation to chronically remodelled airways. Highly significant correlations between regulation of miRNAs expression and several biological processes or regulatory pathways have been found at each step of the evolution of allergen-induced asthma in mice. Similar results were obtained by comparing our data with two different databases using different computational analyses, further reinforcing the significance of this study. Although direct confirmation of the specific implication *in vivo* of the most promising miRNA-mRNA pairs have yet to be performed, the miRNAs profiling data described in the present work are new information allowing a better understanding of molecular mechanisms participating in this complex and progressive pathology and might lead to novel therapeutic approaches targeting either some specific miRNAs of the regulatory processes identified here.

## Methods

### Mouse model of asthma

**Figure 10:**
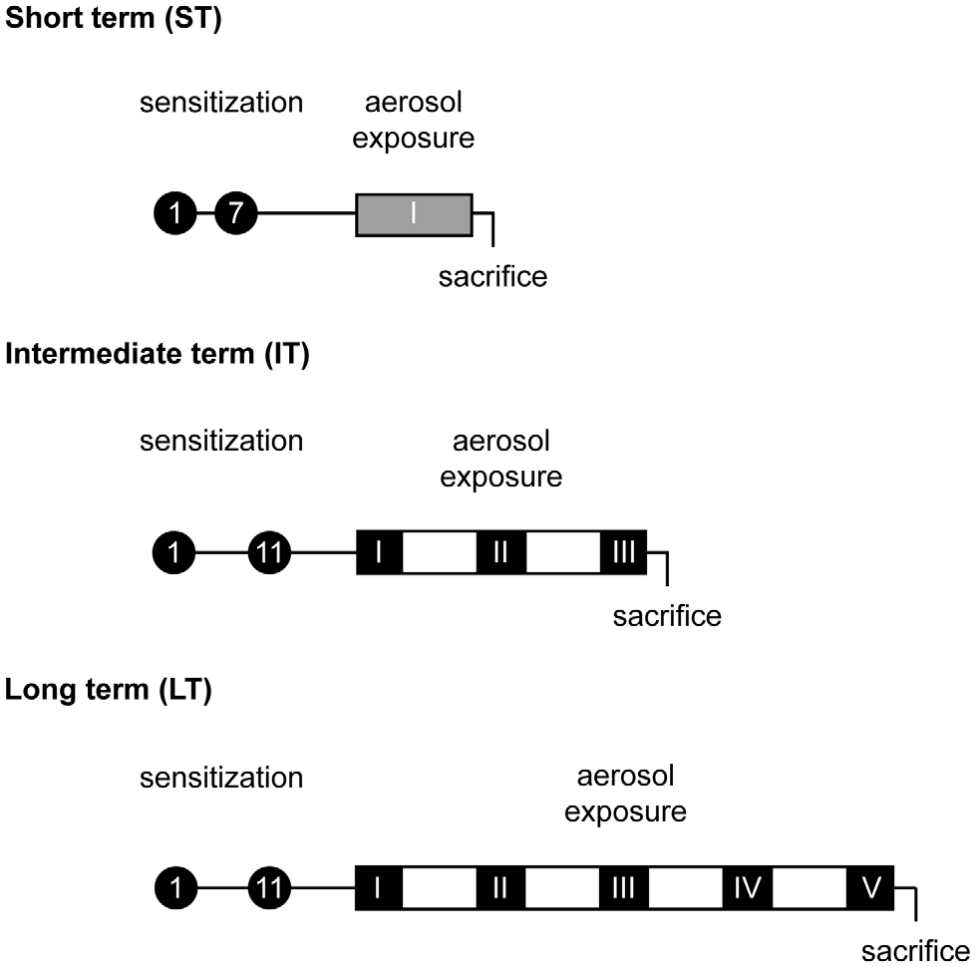
Experimental protocol. Sensitization and short-term (ST), intermediate-term (IT) and long-term (LT) PBS/ovalbumin (OVA) exposure protocols. BALB/c male mice were sensitized on days 1 and 7 (ST) or 11 (IT and LT) by intraperitoneal injection of 10 µg OVA. At day 22, mice were subsequently exposed to PBS or OVA 1% aerosol for 30 min per day. For ST, aerosol challenge was performed for 7 consecutive days (grey box). For IT or LT, aerosol challenges were performed three or five times (black boxes) according to a pattern of 5-day inhalation (black boxes) followed by a 9-day time off (white boxes). Mice were sacrificed the day after last aerosol challenge.

BALB/c mice were used following “Principles of Laboratory Animal Care” formulated by the National Society for Medical Research, and the experimental protocols were approved by the local animal ethical committee (University of Liège) under the no. 03/158. Six- to 8-week old BALB/c male mice were sensitized on days 1 and 7 or 11 (see below) by intraperitoneal injection of 10 µg of ovalbumin (OVA Grade III; Sigma-Aldrich, Schnelldorf, Germany) emulsified in aluminum hydroxide (AlumInject; Perbio, Erembodegem, Belgium). Animals were subsequently divided into 2 groups of six animals: 1 group of control mice was exposed to phosphate buffer saline (PBS) aerosol and the other experimental group was subjected to ovalbumin (OVA) aerosol 1% aerosol for 30 min. A three time-points, named “short-term” (ST), “intermediate-term” (IT) and “long-term” (LT), exposure protocol was designed to reproduce the airway hyperresponsiveness, the inflammation phase and the remodelling process observed during the course of asthma development. At day 21, aerosols were generated daily by ultrasonic nebulizer (DeVilbiss 2000) and performed for 7 consecutive days (ST protocol). For IT or LT protocols, aerosols were performed three (IT) or five (LT) times according to a pattern of 5-day inhalation followed by a 9-day time off. After determination of airway reactivity, mice were sacrificed by cervical dislocation the day after last aerosol challenge. The experimental protocols are illustrated in Figure 10.

### Lung tissue processing and RNA extraction

For each time-point experiment (ST, IT or LT), PBS-treated (n = 6) and OVA-treated (n = 6) mice were killed by cervical dislocation. Further to lung eosinophilia, differential cell counts were performed in bronchoalveolar lavages (BAL) in PBS- and OVA-treated mice. After sacrifice of mice, a cannula was placed in the trachea and a BAL was performed by gentle manual instillation and aspiration using 4×1 ml of 0.05 mM cold PBS-EDTA (Calbiochem, Germany). The BAL was centrifuged (250 g, 10 min, 4°C). The cell pellet was resuspended in 1 ml of PBS-EDTA. Total cells counts were obtained using a hemocytometer. The differential cell counts were performed using morphological criteria on cytocentrifuged preparations (Cytospin) after staining with Diff-Quick (Dade, Belgium). Differential cell counts were performed by one observer unaware of the various experimental conditions.

Immediately after BAL, the right lobe of the lung was stored in liquid nitrogen. The left lobe was used for histological assessment of airway remodeling histology as previously described [4].

Tissue disruption was performed with a Mikro-Dismembrator in liquid nitrogen and tissue powder (50 to 100 mg) was directly homogeneized in 1 ml of Trizol reagent. Phase separation was performed according to reagent protocol. Total RNA was precipitated by an overnight incubation in 0.5 ml of isopropanol at −80°C. Centrifugation (12,000 g, 10 min, 4°C) was then performed and RNA pellet was washed twice by ethanol 75% and centrifugation (7,500 g, 5 min, 4°C). Total RNA pellet was briefly air-dried in a sterile hood, finally dissolved in RNase free water (50 to 100 µl) and stored at −80°C.

### RNA quality control

Each total RNA sample concentration was determined using a NanoDrop ND-1000 spectrophotometer. The integrity of each total RNA extract was assessed with an Agilent 2100 Bioanalyzer and degraded samples were rejected. The passing criteria for use in miRNA microarray and quantitative real-time PCR was a 28S/18S rRNA ratio between 0.90 and 1.80 (maximum obtained). The presence of small RNA was evidenced by a clear peak at about 25 seconds.

### miRNA microarray analysis

Microarray assay was performed using a service provider (LC Sciences, Houston, Texas). The assay started with 5 µg total RNA pools containing equal amount of RNA from individual lung (n = 6 per pool), which was size-fractionated using a YM-100 Microcon centrifugal filter (Millipore, Billerica, MA), and the isolated small RNAs (<300 nt) were 3′-extended with a poly(A) tail using poly(A) polymerase. An oligonucleotide tag was then ligated to the poly(A) tail for later fluorescent dye staining (Cy3 and Cy5 were used for the two RNA pools, OVA versus PBS for each time-point, in a duplicate experiment). Hybridization was performed overnight on a µParaflo microfluidic chip using a microcirculation pump (Atactic Technologies, Houston, TX) [120]. On the microfluidic chip, each detection probe consisted of a chemically modified nucleotide coding a segment complementary to target microRNA (with mice probe content from miRBase, version 10.0, http://microrna.sanger.ac.uk/sequences/) or other control RNA, and a spacer segment of polyethylene glycol to extend the coding segment away from the substrate. The detection probes were made by *in situ* synthesis using photogenerated reagent chemistry.

Hybridization used 100 µl 6× SSPE buffer (0.90 M NaCl, 60 mM Na_2_HPO_4_, 6 mM EDTA, pH 6.8) containing 25% formamide at 34°C. After RNA hybridization, tag-conjugating Cy3 or Cy5 dyes were circulated through the microfluidic chip for dye staining. Fluorescence images were collected using a laser scanner (GenePix 4000B, Molecular Devices, Sunnyvale, CA) and digitized using Array-Pro image analysis software (Media Cybernetics, Bethesda, MD). Data were analyzed by first substracting the background and then normalizing with a cyclic LOWESS filter (Locally-weighted Regression) [121]. For two color experiments (OVA *versus* PBS pools for each time-point), the ratio (log transformed) of the two sets of detected signals, and *p-values* of the *t-test*, were calculated. Differentially detected signals was accepted as true when the ratios of the *p-value* was less than 0.01. Results were finally given in terms of “fold induction”.

### Real-time quantitative RT-PCR validation

In order to reassess the reproducibility of our mouse model asthma and to use in this study the previously described transcriptomic data [4], the expression of 8 genes regulated at least at one time-point was measured for each individual mouse. Total RNA (1 µg) was reverse transcribed using random primers (Eurogentec, Belgium) and SuperScript III Reverse Transcriptase (Invitrogen) according to manufacturer's protocol. cDNAs were then subjected to real time PCR using SYBR Green qPCR Mastermix buffer (Eurogentec, Belgium). Primers used to amplify Ccl8, Fcgr2b, Chia, Birc5, Cdc2a, Pon1, Arg1 and Mmp-12 have been previously described [4]. Expression levels of each mRNA were evaluated using comparative threshold cycle (Ct) method, normalized to those of β2-microglobulin and hypoxanthine guanine phosphoribosyl transferase 1 (house-keeping genes) for each condition (delta Ct). Experiments were done at least in duplicate. The fold change of each mRNA was calculated as a difference between mean values obtained for OVA- and PBS-treated mice at each time-point (delta delta Ct). The fold difference in expression was calculated using the 2^-delta delta Ct^ method. Mean values of fold difference for each cohort used here were compared to those obtained previously [4] (Figure S1).

In order to validate miRNA microarray analysis and to evaluate inter-individual variation in each group of mice, real-time quantitative RT-PCR assays (QuantiMir RT Kit Small RNA Quantitation System, SBI System Biosciences, USA) were on individual lung RNA sample to confirm the differential expression of 14 miRNAs. The selection of the miRNAs is based on their potential role in the pathology of the lung (mmu-miR-1, 146b, -203, -21, -223, -29b, -29c) or on their high and significant differential expression in the model (mmu-miR-455, -574-5p, -672, -690) or for their high (mmu-let-7b a, mmu-miR-145) or low (mmu-miR-450a-5p) signal intensity in microarray analysis. The protocol is based on poly(A) tailing and reverse transcription of small non-coding RNAs to provide cDNA synthesis of miRNA. A universal 3′-tag sequence is incorporated during reverse transcription to enable miRNA expression analysis by quantitative PCR. The reverse sense primer is universal and provided by the kit. The forward sense primer is designed by using the sequence of the mature miRNA (5′-19 nucleotides) converted in DNA sequence (Table 6). Forward LNA primers (Exiqon, Denmark) were used to amplify mmu-miR-146b, -29b and 29c in order to increase specificity and discrimination that were not obtained with native DNA primers. Reactions are performed following manufacturer's protocol with 2 µg of total RNA and SYBR Green qPCR Mastermix buffer (Eurogentec, Belgium). Expression levels of each mature miRNA were evaluated using comparative threshold cycle (Ct) method, normalized to those of U6 small nuclear RNA (house-keeping gene) for each condition (delta Ct). The fold change (OVA-PBS means) of each miRNA was calculated and expressed as 2^−delta delta Ct^. Mean values of fold difference for each cohort were compared to mean values obtained for pools analyzed by microarray hybridization.

**Table 6:** Mature miRNA sequences and forward primers sequences for real-time quantitative PCR.

miRNA	miRBase #	miRNA sequence	Forward primer sequence
mmu-let-7b	MIMAT0000522	5′ - ugagguaguagguugugugguu – 3′	5′ - tgaggtagtaggttgtgtg – 3′
mmu-miR-1	MIMAT0000123	5′ - uggaauguaaagaaguauguau - 3′	5′ - tggaatgtaaagaagtatg - 3′
mmu-miR-145	MIMAT0000157	5′ - guccaguuuucccaggaaucccu - 3′	5′ - gtccagttttcccaggaat - 3′
mmu-miR-203	MIMAT0000236	5′ - gugaaauguuuaggaccacuag - 3′	5′ - gtgaaatgtttaggaccac - 3′
mmu-miR-21	MIMAT0000530	5′ - uagcuuaucagacugauguuga - 3′	5′ - tagcttatcagactgatgt - 3′
mmu-miR-223	MIMAT0000665	5′ - ugucaguuugucaaauacccca - 3′	5′ - tgtcagtttgtcaaatacc - 3′
mmu-miR-450a-5p	MIMAT0001546	5′ - uuuugcgauguguuccuaauau - 3′	5′ - ttttgcgatgtgttcctaa - 3′
mmu-miR-455	MIMAT0003742	5′ - gcaguccacgggcauauacac - 3′	5′ - gcagtccacgggcatatac - 3′
mmu-miR-574-5p	MIMAT0004893	5′ - ugagugugugugugugagugugu - 3′	5′ - tgagtgtgtgtgtgtgagt – 3′
mmu-miR-672	MIMAT0003735	5′ - ugagguugguguacuguguguga - 3′	5′ - tgaggttggtgtactgtgt - 3′
mmu-miR-690	MIMAT0003469	5′ - aaaggcuaggcucacaaccaaa - 3′	5′ - aaaggctaggctcacaacc - 3′

### mRNA microarray analysis

Microarray raw mRNA expression data were from [4] and are in full agreement with RT-qPCR data obtained in this study for new cohort of mice. Probe-level data was background-adjusted and normalized, and gene expression measurements were obtained with the robust multi-array average (RMA) method [122], as implemented in the Affy package of Bioconductor. For each of the three time points, the gene expression log ratios were computed between the PBS control and from two different OVA-treated pools. Genes with a mean OVA/PBS log ratio more than 0.6 or less than −0.6 were selected as up-regulated or down-regulated, respectively. These values correspond to genes regulated 1.52-fold up or down.

When more than one expression value was present for a gene (multiple probe sets per gene), the gene was considered up- or down-regulated if the direction of change (up/down) was consistent between all probe sets, and at least one of the probe sets satisfied the -0.6/0.6 threshold on log ratios.

### miRNA-mRNA correlation analysis

Potential correlations between miRNAs and mRNAs regulated at each time-point were determined by crossing the lists of modulated mRNAs and miRNAs with a database of miRNA target predictions. The list of candidates mRNA targets for each miRNA was retrieved from the MicroCosm Targets database Version 5 (http://www.ebi.ac.uk/enright-srv/microcosm), formely known as mirBase::Targets [12], that uses the miRanda algorithm [123] to identify potential binding sites for a given miRNA in gene sequences. All modulated miRNAs were represented in this database. 

Fifteen modulated genes (among 1955 in total) were not predicted as targets of any miRNAs.

At each time-point, we determined all the miRNA-mRNA pairs such that the mRNA is predicted as a potential target of the miRNA and the miRNA and the mRNA are differentially expressed in opposite direction, i.e. the miRNA is down-regulated and the mRNA is up-regulated, or the opposite. Tables S2, S3, and S4 list all these pairs respectively at ST, IT and LT. The *p-values* in these tables are the *p-values* associated with the target prediction as described in [124] (only targets with a *p-value* lower than 0.05 are included in the MicroCosm Targets database).

### Pathway analysis

A pathway enrichment analysis was carried out taking into account both modulated miRNAs and mRNAs. A list of 117 pathways was downloaded from WikiPathways (http://www.wikipathways.org, [125]). Predicted candidates mRNA targets for each differentially expressed miRNA were identified using two databases: MicroCosm Targets Version 5 (http://www.ebi.ac.uk/enright-srv/microcosm) and TargetScan Version 4.2 (http://www.targetscan.org).

For each pathway and each time-point, a *p-value* reflecting the enrichment of the pathway in modulated miRNA targets (with at least 1.5-fold, up or down) was computed as follows: we counted the total number of miRNA-gene pairs such that the miRNA is modulated, the gene belongs to the pathway and is a potential target of the miRNA. A *p-value* was then associated to this number by computing the proportion of times (estimated from 1000 trials) a purely random selection of the modulated miRNAs (among the 566 miRNAs on the chip) gives a number of pairs as high as the observed one. A *p-value* reflecting the enrichment of the pathway in modulated mRNAs was computed from the number of modulated genes in the pathway using the hypergeometric distribution (using the set of 20461 genes of the chip as a reference set). For this analysis, the set of modulated genes was defined as the set of genes with a fold induction greater than 1.5 in both experiments. A unique *p-value* was finally associated to each pathway by combining the miRNA and mRNA *p-values* using Stouffer's method [126].

### Functional validation

#### Cell Culture and Reagents

SV40-transformed human lung fibroblasts WI26 were grown in Dulbecco's modified Eagle's medium (DMEM, Invitrogen) supplemented with 10% FBS (Lonza). All cells were incubated at 37°C in a humidified chamber supplemented with 5% CO_2_.

#### Plasmid constructs

The luciferase-UTR reporter plasmids were constructed by introducing 3′UTR of 21 genes carrying putative miRNA binding sites into pGL3-promoter vector that contains an SV40 promoter upstream of the luciferase gene (#E1761, Promega). 3′UTR sequences were amplified from WI26 cDNA (for human 3′UTRs) and from total lung Balb/c mice cDNA (for murine 3′UTRs) by PCR (see Table 7 for amplified 3′UTRs and primers) using the Easy-A® One-Tube RT-PCR System (#600182, Agilent Technologies-Stratagene Products). After double digestion by FseI and XbaI of the pGL3-promoter vector (#E1761, Promega), the 3′UTRs were cloned into this vector using the In-Fusion cloning kit (#639619, ClonTech). The correct insertion of these 3′UTRs into the pGL3-promoter vector was verified by restriction analysis and sequencing.

**Table 7:** Genes, species and their amplified 3′UTRs with corresponding primers.

Gene	Species	3'UTR size*	Forward Primer	Reverse Primer
Arid4b	*M. musculus*	2385	GCCGTGTAATTCTAGAacaacaaaaagaagggaaaagg	GTCTGCTCGAAGCGGgaatttacggtttgatttggtg
Card10	*M. musculus*	1727	GCCGTGTAATTCTAGAggaggtgactgagaagaatgtcc	GTCTGCTCGAAGCGGtctatgacaaactttaatgactcattg
Cd3g	*M. musculus*	603	GCCGTGTAATTCTAGAtgtgaaaactgcattgagctaaa	GTCTGCTCGAAGCGGtaggttatacttgatcttttaattttgtca
Cdca8	*M. musculus*	786	GCCGTGTAATTCTAGActcccgggtcttcaagact	GTCTGCTCGAAGCGGgacttaaaaccatggcaaactaaga
Col6a2	*M. musculus*	849	GCCGTGTAATTCTAGAtggccttcccactgaccta	GTCTGCTCGAAGCGGaacagggaggctcaaaacct
Ctse	*M. musculus*	980	GCCGTGTAATTCTAGAtgccctggatggaatcc	GTCTGCTCGAAGCGGgatatttaaaatcaaatcagtttatgggtt
Ctsk	*M. musculus*	759	GCCGTGTAATTCTAGAagaggttctaggggcagcc	GTCTGCTCGAAGCGGttgtaaatgagatactttatttcaaataca
Dera	*M. musculus*	848	GCCGTGTAATTCTAGAcctggtcaaggaggaactagg	GTCTGCTCGAAGCGGatttaaaaattcagcgatatccaca
Fst	*M. musculus*	1410	GCCGTGTAATTCTAGAaaaaaatgcctatgggattcc	GTCTGCTCGAAGCGGtcactcatcatttatctacaaatacacatt
Gmnn	*M. musculus*	980	GCCGTGTAATTCTAGAgcgcgtcagctaccg	GTCTGCTCGAAGCGGttttacaatgttcaacaggaaattg
Il6	*M. musculus*	1090	GCCGTGTAATTCTAGAccaagaacgatagtcaattccag	GTCTGCTCGAAGCGGaaatataatataatttatttgtttgaagacagtctaa
Lpin2	*M. musculus*	3069	GCCGTGTAATTCTAGAcagactgtaggatatttactgtgaatcc	GTCTGCTCGAAGCGGttatcagtttatgttagtttattattgtaacattt
Mmp-15	*M. musculus*	1772	GCCGTGTAATTCTAGAgcagcccagaaccctctc	GTCTGCTCGAAGCGGaacactgtatttctgttttatttagaaatgat
Mmp-15	*H. sapiens*	1439	GCCGTGTAATTCTAGAtaacggtgctcaggggg	GTCTGCTCGAAGCGGtttgccggctgtacaattta
Mmp-24	*H. sapiens*	2353	GCCGTGTAATTCTAGAcaggcccttcctcacca	GTCTGCTCGAAGCGGcactctgtatttctgttttatttagaaa
Mmp-24	*M. musculus*	2318	GCCGTGTAATTCTAGAcaggtagcacccgcagc	GTCTGCTCGAAGCGGgcaggttccagtgcattttatt
Nola2	*M. musculus*	1004	GCCGTGTAATTCTAGAggccgcgtggttcct	GTCTGCTCGAAGCGGgttttataactttgagcaaatatattcatag
Nusap1	*M. musculus*	1400	GCCGTGTAATTCTAGAcctcaactacaagccacacaaa	GTCTGCTCGAAGCGGgctaccacattcagcttagcttt
Phb2	*M. musculus*	640	GCCGTGTAATTCTAGAagagtacacagctgctgtagaagc	GTCTGCTCGAAGCGGggctttaagtaataaaaattttattgagaa
Scube2	*M. musculus*	836	GCCGTGTAATTCTAGAattcagttcaagtccaatgaagg	GTCTGCTCGAAGCGGtttaatcatcactgtttagaactcacac
Ube2c	*M. musculus*	931	GCCGTGTAATTCTAGAgcagttgccctttcctctc	GTCTGCTCGAAGCGGacaaaacaatcaatgtgtatttatttaat

*Size (bp) of amplified 3′UTRs for each target.

#### Transfection

WI26 cells were transfected using jetPRIME reagent (Polyplus transfection) following the manufacturer's protocol for DNA and miRNA mimic cotransfection. In brief, 60 000 cells were seeded in a 24-well plate 24 hours prior transfection. They were transfected on the following day with 250 ng of plasmid DNA and with 2.5 to 30 nM miRNA mimic (miScript miRNA Mimic, Qiagen) (Table 8) or 20 nM AllStars Negative Control siRNA (Qiagen) per well. Transfection medium was then replaced by cell growth medium 24 hours after transfection.

**Table 8:** Targeted 3′UTR sequences, corresponding miRNA Mimic and concentrations used.

Gene	miScript miRNA Mimic	Concentrations
Arid4b	Syn-hsa-miR-223	20 nM
Card10	Syn-hsa-miR-146b	2.5, 5, 10, 20, 30 nM
Cd3g	Syn-hsa-miR-672	20 nM
Cdca8	Syn-hsa-miR-690	20 nM
Col6a2	Syn-hsa-miR-29c	2.5, 5, 10, 20, 30 nM
Ctse	Syn-hsa-miR-690	20 nM
Ctsk	Syn-hsa-miR-29c	2.5, 5, 10, 20, 30 nM
Dera	Syn-hsa-miR-574-5p	20 nM
Fst	Syn-hsa-miR-690	20 nM
Gmnn	Syn-hsa-miR-483	20 nM
Il6	Syn-hsa-miR-223	20 nM
Lpin2	Syn-hsa-miR-223	20 nM
Mmp-15 (m, h)	Syn-hsa-miR-29b, Syn-hsa-miR-29c	2.5, 5, 10, 20, 30 nM
Mmp-24 (m, h)	Syn-hsa-miR-29b, Syn-hsa-miR-29c	2.5, 5, 10, 20, 30 nM
Nola2	Syn-hsa-miR-483	20 nM
Nusap1	Syn-hsa-miR-574-5p	20 nM
Phb2	Syn-hsa-miR-672	20 nM
Scube2	Syn-hsa-miR-146b	2.5, 5, 10, 20, 30 nM
Ube2c	Syn-hsa-miR-483	20 nM

(m, h): murine and human 3′UTRs.

#### Luciferase Assay

Luciferase assays were carried out in WI26 cells 40 hours after transfection as a way to determine the effect of microRNAs on the activity of Luc-3′UTRs. Cells were harvested, lysed and luciferase activity was determined by using Luciferase Reporter Gene Assay, high sensitivity (Roche Applied Science, Belgium) according to the manufacturer's protocol. Protein quantification was used for normalization. Knockdown was calculated as a ratio comparing for each construct the luciferase activity measured in presence of the specific miRNA to the activity obtained in presence of the non-functional control.

Experiments were done at least in duplicate and data are presented as means ± SD. Statistical analyses were performed using unpaired two-tailed Student's t-tests.

## Supporting Information

Figure S1 **mRNA expression profiles in our models of allergen-induced asthma.** Validation of reproducibility of our models of allergen-induced asthma was assessed by comparing the mRNA level of a series of selected genes measured by RT-qPCR (in duplicate) on individual lung RNA in each group (ST, IT LT) of PBS- and OVA-treated mice to data obtained by microarray analysis in a previous study [4]. The graphs illustrate a concordance of 8 significantly modulated genes. mRNA levels were normalized by using β2-microglobulin and hypoxanthine guanine phosphoribosyl transferase transcripts. Differences (fold induction) between samples (OVA vs PBS) were calculated using the 2^-delta delta Ct^ method. Results are expressed as mean ± SD.
(TIF)

Table S1 Significantly modulated mature miRNAs and their respective fold induction for each time-point experiment.
(DOC)

Table S2 Modulated mRNAs and inversely correlated modulated miRNAs at ST using MicroCosm Targets.
(DOC)

Table S3 Modulated mRNAs and inversely correlated modulated miRNAs at IT using MicroCosm Targets.
(DOC)

Table S4 Modulated mRNAs and inversely correlated modulated miRNAs at using MicroCosm Targets.
(DOC)

Table S5 MiRNAs/mRNAs regulatory pathways at ST using MicroCosm Targets.
(DOC)

Table S6 MiRNAs/mRNAs regulatory pathways at IT using MicroCosm Targets.
(DOC)

Table S7 MiRNAs/mRNAs regulatory pathways at LT using MicroCosm Targets.
(DOC)

Table S8 MiRNAs/mRNAs regulatory pathways in mice model of asthma at ST using TargetScan.
(DOC)

Table S9 MiRNAs/mRNAs regulatory pathways at IT using TargetScan.
(DOC)

Table S10 MiRNAs/mRNAs regulatory pathways at LT using TargetScan.
(DOC)

## References

[1] Lange P, Parner J, Vestbo J, Schnohr P, Jensen G (1998). A 15-year follow-up study of ventilatory function in adults with asthma.. N Engl J Med.

[2] Pascual RM, Peters SP (2005). Airway remodeling contributes to the progressive loss of lung function in asthma: an overview.. J Allergy Clin Immunol.

[3] Hirst SJ, Martin JG, Bonacci JV, Chan V, Fixman ED (2004). Proliferative aspects of airway smooth muscle.. J Allergy Clin Immunol.

[4] Di Valentin E, Crahay C, Garbacki N, Hennuy B, Gueders M (2009). New asthma biomarkers: lessons from murine models of acute and chronic asthma.. Am J Physiol Lung Cell Mol Physiol.

[5] Pennings JL, Kimman TG, Janssen R (2008). Identification of a common gene expression response in different lung inflammatory diseases in rodents and macaques.. PLoS ONE.

[6] Novershtern N, Itzhaki Z, Manor O, Friedman N, Kaminski N (2008). A functional and regulatory map of asthma.. Am J Respir Cell Mol Biol.

[7] Rolph MS, Sisavanh M, Liu SM, Mackay CR (2006). Clues to asthma pathogenesis from microarray expression studies.. Pharmacol Ther.

[8] Izuhara K, Saito H (2006). Microarray-based identification of novel biomarkers in asthma.. Allergol Int.

[9] Park SG, Choi JW, Kim H, Roh GS, Bok J (2008). Genome-wide profiling of antigen-induced time course expression using murine models for acute and chronic asthma.. Int Arch Allergy Immunol.

[10] Zimmermann N, King NE, Laporte J, Yang M, Mishra A (2003). Dissection of experimental asthma with DNA microarray analysis identifies arginase in asthma pathogenesis.. J Clin Invest.

[11] Bartel DP (2004). MicroRNAs: genomics, biogenesis, mechanism, and function.. Cell.

[12] Griffiths-Jones S, Saini HK, van Dongen S, Enright AJ (2008). miRBase: tools for microRNA genomics.. Nucleic Acids Res.

[13] Erson AE, Petty EM (2008). MicroRNAs in development and disease.. Clin Genet.

[14] Zhang B, Farwell MA (2008). microRNAs: a new emerging class of players for disease diagnostics and gene therapy.. J Cell Mol Med.

[15] Wang Y, Liang Y, Lu Q (2008). MicroRNA epigenetic alterations: predicting biomarkers and therapeutic targets in human diseases.. Clin Genet.

[16] Zhang B, Pan X, Cobb GP, Anderson TA (2007). microRNAs as oncogenes and tumor suppressors.. Dev Biol.

[17] Calin GA, Croce CM (2006). MicroRNA signatures in human cancers.. Nat Rev Cancer.

[18] Esquela-Kerscher A, Slack FJ (2006). Oncomirs - microRNAs with a role in cancer.. Nat Rev Cancer.

[19] Wiemer EA (2007). The role of microRNAs in cancer: no small matter.. Eur J Cancer.

[20] Ma L, Teruya-Feldstein J, Weinberg RA (2007). Tumour invasion and metastasis initiated by microRNA-10b in breast cancer.. Nature.

[21] Thum T, Catalucci D, Bauersachs J, Thum T, Catalucci D (2008). MicroRNAs: novel regulators in cardiac development and disease. [see comment].. Cardiovascular Research.

[22] Catalucci D, Latronico MV, Condorelli G, Catalucci D, Latronico MVG (2008). MicroRNAs control gene expression: importance for cardiac development and pathophysiology.. Annals of the New York Academy of Sciences.

[23] Callis TE, Chen JF, Wang DZ, Callis TE, Chen J-F (2007). MicroRNAs in skeletal and cardiac muscle development.. DNA & Cell Biology.

[24] Poy MN, Eliasson L, Krutzfeldt J, Kuwajima S, Ma X (2004). A pancreatic islet-specific microRNA regulates insulin secretion.. Nature.

[25] Hennessy E, O'Driscoll L, Hennessy E, O'Driscoll L (2008). Molecular medicine of microRNAs: structure, function and implications for diabetes.. Expert Reviews in Molecular Medicine.

[26] Esau C, Davis S, Murray SF, Yu XX, Pandey SK (2006). miR-122 regulation of lipid metabolism revealed by in vivo antisense targeting.. Cell Metab.

[27] Poy MN, Spranger M, Stoffel M (2007). microRNAs and the regulation of glucose and lipid metabolism.. Diabetes Obes Metab.

[28] Gottwein E, Cullen BR, Gottwein E, Cullen BR (2008). Viral and cellular microRNAs as determinants of viral pathogenesis and immunity.. Cell Host & Microbe.

[29] Lecellier CH, Dunoyer P, Arar K, Lehmann-Che J, Eyquem S (2005). A cellular microRNA mediates antiviral defense in human cells.. Science.

[30] Cullen BR (2006). Viruses and microRNAs.. Nat Genet.

[31] Izzotti A, Calin GA, Arrigo P, Steele VE, Croce CM (2009). Downregulation of microRNA expression in the lungs of rats exposed to cigarette smoke.. Faseb J.

[32] Nana-Sinkam SP, Hunter MG, Nuovo GJ, Schmittgen TD, Gelinas R (2009). Integrating the MicroRNome into the study of lung disease.. Am J Respir Crit Care Med.

[33] Moschos SA, Williams AE, Perry MM, Birrell MA, Belvisi MG (2007). Expression profiling in vivo demonstrates rapid changes in lung microRNA levels following lipopolysaccharide-induced inflammation but not in the anti-inflammatory action of glucocorticoids.. BMC Genomics.

[34] Williams AE, Larner-Svensson H, Perry MM, Campbell GA, Herrick SE (2009). MicroRNA expression profiling in mild asthmatic human airways and effect of corticosteroid therapy.. PLoS ONE.

[35] Lu TX, Munitz A, Rothenberg ME (2009). MicroRNA-21 is up-regulated in allergic airway inflammation and regulates IL-12p35 expression.. J Immunol.

[36] Mattes J, Collison A, Plank M, Phipps S, Foster PS (2009). Antagonism of microRNA-126 suppresses the effector function of TH2 cells and the development of allergic airways disease.. Proc Natl Acad Sci U S A.

[37] Chiba Y, Tanabe M, Goto K, Sakai H, Misawa M (2009). Down-Regulation of miR-133a Contributes to Up-Regulation of RhoA in Bronchial Smooth Muscle Cells.. Am J Respir Crit Care Med.

[38] Polikepahad S, Knight JM, Naghavi AO, Oplt T, Creighton CJ (2010). Proinflammatory role for let-7 microRNAS in experimental asthma.. J Biol Chem.

[39] Southam DS, Ellis R, Wattie J, Glass W, Inman MD (2008). Goblet cell rebound and airway dysfunction with corticosteroid withdrawal in a mouse model of asthma.. Am J Respir Crit Care Med.

[40] Gueders MM, Paulissen G, Crahay C, Quesada-Calvo F, Hacha J (2009). Mouse models of asthma: a comparison between C57BL/6 and BALB/c strains regarding bronchial responsiveness, inflammation, and cytokine production.. Inflamm Res.

[41] Van Hove CL, Maes T, Cataldo DD, Gueders MM, Palmans E (2009). Comparison of acute inflammatory and chronic structural asthma-like responses between C57BL/6 and BALB/c mice.. Int Arch Allergy Immunol.

[42] Gueders MM, Bertholet P, Perin F, Rocks N, Maree R (2008). A novel formulation of inhaled doxycycline reduces allergen-induced inflammation, hyperresponsiveness and remodeling by matrix metalloproteinases and cytokines modulation in a mouse model of asthma.. Biochemical Pharmacology.

[43] Tang X, Muniappan L, Tang G, Ozcan S (2009). Identification of glucose-regulated miRNAs from pancreatic {beta} cells reveals a role for miR-30d in insulin transcription.. Rna.

[44] Malumbres R, Sarosiek KA, Cubedo E, Ruiz JW, Jiang X (2009). Differentiation stage-specific expression of microRNAs in B lymphocytes and diffuse large B-cell lymphomas.. Blood.

[45] Baltimore D, Boldin MP, O'Connell RM, Rao DS, Taganov KD (2008). MicroRNAs: new regulators of immune cell development and function.. Nat Immunol.

[46] Bellon M, Lepelletier Y, Hermine O, Nicot C (2009). Deregulation of microRNA involved in hematopoiesis and the immune response in HTLV-I adult T-cell leukemia.. Blood.

[47] Johnnidis JB, Harris MH, Wheeler RT, Stehling-Sun S, Lam MH (2008). Regulation of progenitor cell proliferation and granulocyte function by microRNA-223.. Nature.

[48] Anglicheau D, Sharma VK, Ding R, Hummel A, Snopkowski C (2009). MicroRNA expression profiles predictive of human renal allograft status.. Proc Natl Acad Sci U S A.

[49] Laios A, O'Toole S, Flavin R, Martin C, Kelly L (2008). Potential role of miR-9 and miR-223 in recurrent ovarian cancer.. Mol Cancer.

[50] Wong QW, Lung RW, Law PT, Lai PB, Chan KY (2008). MicroRNA-223 is commonly repressed in hepatocellular carcinoma and potentiates expression of Stathmin1.. Gastroenterology.

[51] Gottardo F, Liu CG, Ferracin M, Calin GA, Fassan M (2007). Micro-RNA profiling in kidney and bladder cancers.. Urol Oncol.

[52] Wang J, Xu R, Lin F, Zhang S, Zhang G (2009). MicroRNA: novel regulators involved in the remodeling and reverse remodeling of the heart.. Cardiology.

[53] Walden TB, Timmons JA, Keller P, Nedergaard J, Cannon B (2009). Distinct expression of muscle-specific microRNAs (myomirs) in brown adipocytes.. J Cell Physiol.

[54] Pekarsky Y, Santanam U, Cimmino A, Palamarchuk A, Efanov A (2006). Tcl1 expression in chronic lymphocytic leukemia is regulated by miR-29 and miR-181.. Cancer Res.

[55] Yanaihara N, Caplen N, Bowman E, Seike M, Kumamoto K (2006). Unique microRNA molecular profiles in lung cancer diagnosis and prognosis.. Cancer Cell.

[56] Porkka KP, Pfeiffer MJ, Waltering KK, Vessella RL, Tammela TL (2007). MicroRNA expression profiling in prostate cancer.. Cancer Res.

[57] Iorio MV, Ferracin M, Liu CG, Veronese A, Spizzo R (2005). MicroRNA gene expression deregulation in human breast cancer.. Cancer Res.

[58] Fabbri M, Garzon R, Cimmino A, Liu Z, Zanesi N (2007). MicroRNA-29 family reverts aberrant methylation in lung cancer by targeting DNA methyltransferases 3A and 3B.. Proc Natl Acad Sci U S A.

[59] Park SY, Lee JH, Ha M, Nam JW, Kim VN (2009). miR-29 miRNAs activate p53 by targeting p85 alpha and CDC42.. Nat Struct Mol Biol.

[60] Sengupta S, den Boon JA, Chen IH, Newton MA, Stanhope SA (2008). MicroRNA 29c is down-regulated in nasopharyngeal carcinomas, up-regulating mRNAs encoding extracellular matrix proteins.. Proc Natl Acad Sci U S A.

[61] Wang H, Garzon R, Sun H, Ladner KJ, Singh R (2008). NF-kappaB-YY1-miR-29 regulatory circuitry in skeletal myogenesis and rhabdomyosarcoma.. Cancer Cell.

[62] Schmidt WM, Spiel AO, Jilma B, Wolzt M, Muller M (2009). In vivo profile of the human leukocyte microRNA response to endotoxemia.. Biochem Biophys Res Commun.

[63] Taganov KD, Boldin MP, Chang KJ, Baltimore D (2006). NF-kappaB-dependent induction of microRNA miR-146, an inhibitor targeted to signaling proteins of innate immune responses.. Proc Natl Acad Sci USA.

[64] Bhaumik D, Scott GK, Schokrpur S, Patil CK, Campisi J (2008). Expression of microRNA-146 suppresses NF-[kappa]B activity with reduction of metastatic potential in breast cancer cells.. Oncogene.

[65] Park SE, Xu J, Frolova A, Liao L, O'Malley BW (2005). Genetic deletion of the repressor of estrogen receptor activity (REA) enhances the response to estrogen in target tissues in vivo.. Mol Cell Biol.

[66] Luo L, Yang X, Takihara Y, Knoetgen H, Kessel M (2004). The cell-cycle regulator geminin inhibits Hox function through direct and polycomb-mediated interactions.. Nature.

[67] Wolgemuth DJ (2008). Function of cyclins in regulating the mitotic and meiotic cell cycles in male germ cells.. Cell Cycle.

[68] Yamanaka Y, Heike T, Kumada T, Shibata M, Takaoka Y (2008). Loss of Borealin/DasraB leads to defective cell proliferation, p53 accumulation and early embryonic lethality.. Mech Dev.

[69] Raemaekers T, Ribbeck K, Beaudouin J, Annaert W, Van Camp M (2003). NuSAP, a novel microtubule-associated protein involved in mitotic spindle organization.. J Cell Biol.

[70] Buhling F, Rocken C, Brasch F, Hartig R, Yasuda Y (2004). Pivotal role of cathepsin K in lung fibrosis.. Am J Pathol.

[71] Ohta K, Yamashita N, Tajima M, Miyasaka T, Kawashima R (2001). In vivo effects of apoptosis in asthma examined by a murine model.. Int Arch Allergy Immunol.

[72] Noma T, Sugawara Y, Aoki K, Kawano Y, Ishikawa Y (2002). Induction of peripheral mononuclear cell apoptosis in asthmatic patients in remission.. J Asthma.

[73] Wang L, Guo Y, Huang WJ, Ke X, Poyet JL (2001). Card10 is a novel caspase recruitment domain/membrane-associated guanylate kinase family member that interacts with BCL10 and activates NF-kappa B.. J Biol Chem.

[74] Yang RB, Ng CK, Wasserman SM, Colman SD, Shenoy S (2002). Identification of a novel family of cell-surface proteins expressed in human vascular endothelium.. J Biol Chem.

[75] Akira S, Kishimoto T (1992). IL-6 and NF-IL6 in acute-phase response and viral infection.. Immunol Rev.

[76] Nishioku T, Hashimoto K, Yamashita K, Liou SY, Kagamiishi Y (2002). Involvement of cathepsin E in exogenous antigen processing in primary cultured murine microglia.. J Biol Chem.

[77] Shin SM, Kim YH, Choi BK, Kwon PM, Lee HW (2007). 4-1BB triggers IL-13 production from T cells to limit the polarized, Th1-mediated inflammation.. J Leukoc Biol.

[78] Polte T, Foell J, Werner C, Hoymann HG, Braun A (2006). CD137-mediated immunotherapy for allergic asthma.. J Clin Invest.

[79] Seo SK, Choi JH, Kim YH, Kang WJ, Park HY (2004). 4-1BB-mediated immunotherapy of rheumatoid arthritis.. Nat Med.

[80] Foell J, Strahotin S, O'Neil SP, McCausland MM, Suwyn C (2003). CD137 costimulatory T cell receptor engagement reverses acute disease in lupus-prone NZB x NZW F1 mice.. J Clin Invest.

[81] Kuang W, Tan J, Duan Y, Duan J, Wang W (2009). Cyclic stretch induced miR-146a upregulation delays C2C12 myogenic differentiation through inhibition of Numb.. Biochem Biophys Res Commun.

[82] Pece S, Serresi M, Santolini E, Capra M, Hulleman E (2004). Loss of negative regulation by Numb over Notch is relevant to human breast carcinogenesis.. J Cell Biol.

[83] Nal B, Mohr E, Silva MI, Tagett R, Navarro C (2002). Wdr12, a mouse gene encoding a novel WD-Repeat Protein with a notchless-like amino-terminal domain.. Genomics.

[84] Mestdagh P, Van Vlierberghe P, De Weer A, Muth D, Westermann F (2009). A novel and universal method for microRNA RT-qPCR data normalization.. Genome Biol.

[85] Tzur G, Levy A, Meiri E, Barad O, Spector Y (2008). MicroRNA expression patterns and function in endodermal differentiation of human embryonic stem cells.. PLoS ONE.

[86] Chang TC, Yu D, Lee YS, Wentzel EA, Arking DE (2008). Widespread microRNA repression by Myc contributes to tumorigenesis.. Nat Genet.

[87] Betel D, Wilson M, Gabow A, Marks DS, Sander C (2008). The microRNA.org resource: targets and expression.. Nucleic Acids Res.

[88] Lewis BP, Shih IH, Jones-Rhoades MW, Bartel DP, Burge CB (2003). Prediction of mammalian microRNA targets.. Cell.

[89] Gueders MM, Foidart JM, Noel A, Cataldo DD (2006). Matrix metalloproteinases (MMPs) and tissue inhibitors of MMPs in the respiratory tract: potential implications in asthma and other lung diseases.. Eur J Pharmacol.

[90] Henderson N, Markwick LJ, Elshaw SR, Freyer AM, Knox AJ (2007). Collagen I and thrombin activate MMP-2 by MMP-14-dependent and -independent pathways: implications for airway smooth muscle migration.. Am J Physiol Lung Cell Mol Physiol.

[91] Zucker S, Drews M, Conner C, Foda HD, DeClerck YA (1998). Tissue inhibitor of metalloproteinase-2 (TIMP-2) binds to the catalytic domain of the cell surface receptor, membrane type 1-matrix metalloproteinase 1 (MT1-MMP).. J Biol Chem.

[92] Murphy G, Nagase H (2008). Progress in matrix metalloproteinase research.. Mol Aspects Med.

[93] Barnes PJ, Drazen JM, Rennard S, Thomson NC (2002). Asthma and COPD, Basic Mechanisms and Clinical Management Academic Press.

[94] Zhu ML, Kyprianou N (2008). Androgen receptor and growth factor signaling cross-talk in prostate cancer cells.. Endocr Relat Cancer.

[95] Recchia AG, Musti AM, Lanzino M, Panno ML, Turano E (2009). A cross-talk between the androgen receptor and the epidermal growth factor receptor leads to p38MAPK-dependent activation of mTOR and cyclinD1 expression in prostate and lung cancer cells.. Int J Biochem Cell Biol.

[96] Yamashita N, Tashimo H, Ishida H, Matsuo Y, Arai H (2005). Role of insulin-like growth factor-I in allergen-induced airway inflammation and remodeling.. Cell Immunol.

[97] Gosens R, Schaafsma D, Grootte Bromhaar MM, Vrugt B, Zaagsma J (2004). Growth factor-induced contraction of human bronchial smooth muscle is Rho-kinase-dependent.. Eur J Pharmacol.

[98] Delafontaine P, Song YH, Li Y (2004). Expression, regulation, and function of IGF-1, IGF-1R, and IGF-1 binding proteins in blood vessels.. Arterioscler Thromb Vasc Biol.

[99] Asirvatham AJ, Magner WJ, Tomasi TB (2009). miRNA regulation of cytokine genes.. Cytokine.

[100] Sheedy FJ, O'Neill LA (2008). Adding fuel to fire: microRNAs as a new class of mediators of inflammation.. Ann Rheum Dis.

[101] Brightling C, Berry M, Amrani Y (2008). Targeting TNF-alpha: a novel therapeutic approach for asthma.. J Allergy Clin Immunol.

[102] Thomas PS (2001). Tumour necrosis factor-alpha: the role of this multifunctional cytokine in asthma.. Immunol Cell Biol.

[103] van Rooij E, Sutherland LB, Thatcher JE, DiMaio JM, Naseem RH (2008). Dysregulation of microRNAs after myocardial infarction reveals a role of miR-29 in cardiac fibrosis.. Proc Natl Acad Sci U S A.

[104] Li Z, Hassan MQ, Jafferji M, Aqeilan RI, Garzon R (2009). Biological functions of miR-29b contribute to positive regulation of osteoblast differentiation.. J Biol Chem.

[105] Kuhn C, Boldt J, King TE, Crouch E, Vartio T (1989). An immunohistochemical study of architectural remodeling and connective tissue synthesis in pulmonary fibrosis.. Am Rev Respir Dis.

[106] Muro AF, Moretti FA, Moore BB, Yan M, Atrasz RG (2008). An essential role for fibronectin extra type III domain A in pulmonary fibrosis.. Am J Respir Crit Care Med.

[107] Shan SW, Lee DY, Deng Z, Shatseva T, Jeyapalan Z (2009). MicroRNA MiR-17 retards tissue growth and represses fibronectin expression.. Nat Cell Biol.

[108] Zhang X, Liu S, Hu T, Liu S, He Y (2009). Up-regulated microRNA-143 transcribed by nuclear factor kappa B enhances hepatocarcinoma metastasis by repressing fibronectin expression.. Hepatology.

[109] Wang Q, Wang Y, Minto AW, Wang J, Shi Q (2008). MicroRNA-377 is up-regulated and can lead to increased fibronectin production in diabetic nephropathy.. Faseb J.

[110] Saotome A, Kanai N, Nagai T, Yashiro T, Tokudome S (2003). Immunohistochemical classification of the localization of laminin in the thickened bronchial epithelial basement membrane of deceased bronchial asthma patients.. Respir Med.

[111] Meeker TC, Hardy D, Willman C, Hogan T, Abrams J (1990). Activation of the interleukin-3 gene by chromosome translocation in acute lymphocytic leukemia with eosinophilia.. Blood.

[112] Asquith KL, Ramshaw HS, Hansbro PM, Beagley KW, Lopez AF (2008). The IL-3/IL-5/GM-CSF common receptor plays a pivotal role in the regulation of Th2 immunity and allergic airway inflammation.. J Immunol.

[113] Fabry B, Fredberg JJ (2007). Mechanotransduction, asthma, and airway smooth muscle.. Drug Discov Today Dis Models.

[114] Turner CE (1998). Molecules in focus Paxillin.. The International Journal of Biochemistry & Cell Biology.

[115] Opazo Saez A, Zhang W, Wu Y, Turner CE, Tang DD (2004). Tension development during contractile stimulation of smooth muscle requires recruitment of paxillin and vinculin to the membrane.. Am J Physiol Cell Physiol.

[116] Ebner S, Hofer S, Nguyen VA, Furhapter C, Herold M (2002). A novel role for IL-3: human monocytes cultured in the presence of IL-3 and IL-4 differentiate into dendritic cells that produce less IL-12 and shift Th cell responses toward a Th2 cytokine pattern.. J Immunol.

[117] Moser M, Murphy KM (2000). Dendritic cell regulation of TH1-TH2 development.. Nat Immunol.

[118] Gardino AK, Smerdon SJ, Yaffe MB (2006). Structural determinants of 14-3-3 binding specificities and regulation of subcellular localization of 14-3-3-ligand complexes: a comparison of the X-ray crystal structures of all human 14-3-3 isoforms.. Semin Cancer Biol.

[119] Agarwal-Mawal A, Qureshi HY, Cafferty PW, Yuan Z, Han D (2003). 14-3-3 connects glycogen synthase kinase-3 beta to tau within a brain microtubule-associated tau phosphorylation complex.. J Biol Chem.

[120] Zhu Q, Hong A, Sheng N, Zhang X, Matejko A (2007). microParaflo biochip for nucleic acid and protein analysis.. Methods Mol Biol.

[121] Bolstad BM, Irizarry RA, Astrand M, Speed TP (2003). A comparison of normalization methods for high density oligonucleotide array data based on variance and bias.. Bioinformatics.

[122] Irizarry RA, Hobbs B, Collin F, Beazer-Barclay YD, Antonellis KJ (2003). Exploration, normalization, and summaries of high density oligonucleotide array probe level data.. Biostatistics.

[123] John B, Enright AJ, Aravin A, Tuschl T, Sander C (2004). Human MicroRNA targets.. PLoS Biol.

[124] Rehmsmeier M, Steffen P, Hochsmann M, Giegerich R (2004). Fast and effective prediction of microRNA/target duplexes.. Rna.

[125] Pico AR, Kelder T, van Iersel MP, Hanspers K, Conklin BR (2008). WikiPathways: pathway editing for the people.. PLoS Biol.

[126] Leroy Folks J, Krishnaiah PR, Sen PK (1984). 6 Combination of independent tests..

