# Supplementary figures and images for: MicroRNAs Profiling in Murine Models of Acute and Chronic Asthma: A Relationship with mRNAs Targets
Source: PLoS One. 2011 Jan 28;6(1):e16509. doi: 10.1371/journal.pone.0016509 (PMC3030602; doi:10.1371/journal.pone.0016509)

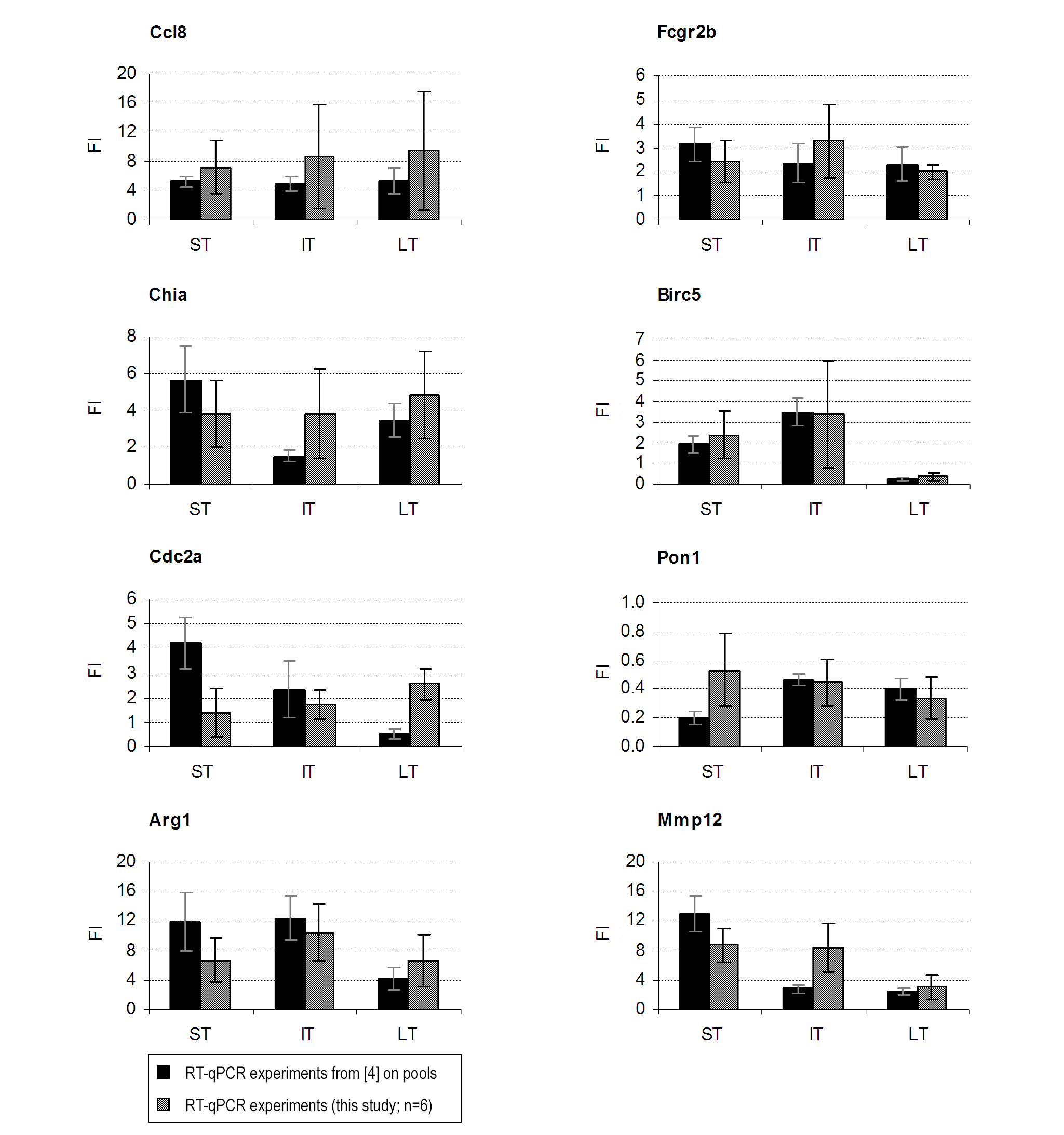

Supplement: Figure S1 — mRNA expression profiles in our models of allergen-induced asthma. Validation of reproducibility of our models of allergen-induced asthma was assessed by comparing the mRNA level of a series of selected genes measured by RT-qPCR (in duplicate) on individual lung RNA in each group (ST, IT LT) of PBS- and OVA-treated mice to data obtained by microarray analysis in a previous study [4]. The graphs illustrate a concordance of 8 significantly modulated genes. mRNA levels were normalized by using β2-microglobulin and hypoxanthine guanine phosphoribosyl transferase transcripts. Differences (fold induction) between samples (OVA vs PBS) were calculated using the 2-delta delta Ct method. Results are expressed as mean ± SD. (TIF) [file pone.0016509.s001.tif]
