# Supplementary material for: MicroRNAs Profiling in Murine Models of Acute and Chronic Asthma: A Relationship with mRNAs Targets
Source: PLoS One. 2011 Jan 28;6(1):e16509. doi: 10.1371/journal.pone.0016509 (PMC3030602; doi:10.1371/journal.pone.0016509)
Supplement: Table S1 — Significantly modulated mature miRNAs and their respective fold induction for each time-point experiment. (DOC) [file pone.0016509.s002.doc]

| **ST** | | **IT** | | **LT** | |
| --- | --- | --- | --- | --- | --- |
| **miRNA** | **FI** | **miRNA** | **FI** | **miRNA** | **FI** |
| mmu-miR-712* | 5.24 | mmu-miR-155 | 4.48 | mmu-miR-705 | 119.30 |
| mmu-miR-122 | 5.08 | mmu-miR-467b* | 2.97 | mmu-miR-188-5p | 117.39 |
| mmu-miR-181d | 2.51 | mmu-miR-467a* | 2.79 | mmu-miR-483 | 115.80 |
| mmu-miR-106a | 2.28 | mmu-miR-466g | 2.64 | mmu-miR-669c | 115.25 |
| mmu-miR-223 | 1.99 | mmu-miR-466f-3p | 2.23 | mmu-miR-568 | 96.03 |
| mmu-miR-146b | 1.91 | mmu-miR-455 | 2.03 | mmu-miR-467b* | 48.35 |
| mmu-miR-181b | 1.91 | mmu-miR-150 | 1.80 | mmu-miR-691 | 42.50 |
| mmu-miR-689 | 1.90 | mmu-miR-423-5p | 1.69 | mmu-miR-671-5p | 39.54 |
| mmu-miR-20b | 1.88 | mmu-miR-146b | 1.55 | mmu-miR-467a* | 39.02 |
| mmu-miR-451 | 1.60 | mmu-miR-375 | 1.52 | mmu-miR-485* | 33.41 |
| mmu-miR-100 | 1.52 | mmu-miR-21 | 1.45 | mmu-miR-744 | 29.56 |
| mmu-miR-93 | 1.46 | mmu-miR-185 | 1.40 | mmu-miR-466f-3p | 22.69 |
| mmu-miR-130a | 1.44 | mmu-miR-200c | 1.35 | mmu-miR-685 | 19.58 |
| mmu-miR-17 | 1.44 | mmu-miR-107 | 1.35 | mmu-miR-709 | 18.98 |
| mmu-miR-486 | 1.42 | mmu-miR-98 | 1.33 | mmu-miR-467e* | 18.51 |
| mmu-miR-107 | 1.38 | mmu-miR-103 | 1.29 | mmu-miR-466c-5p | 17.07 |
| mmu-miR-29c | 1.38 | mmu-miR-320 | 1.28 | mmu-miR-466g | 16.19 |
| mmu-miR-181a | 1.37 | mmu-let-7f | 1.26 | mmu-miR-574-3p | 15.99 |
| mmu-miR-126-3p | 1.36 | mmu-miR-705 | 1.26 | mmu-miR-574-5p | 13.18 |
| mmu-miR-103 | 1.35 | mmu-miR-181a | 1.24 | mmu-miR-667 | 13.13 |
| mmu-miR-20a | 1.35 | mmu-miR-25 | 1.23 | mmu-miR-713 | 11.83 |
| mmu-miR-30e | 1.35 | mmu-let-7a | 1.22 | mmu-let-7d* | 11.72 |
| mmu-miR-150 | 1.34 | mmu-let-7b | 1.22 | mmu-miR-762 | 11.22 |
| mmu-miR-25 | 1.33 | mmu-miR-1 | 1.21 | mmu-miR-466d-3p | 9.95 |
| mmu-miR-21 | 1.26 | mmu-miR-92a | 1.19 | mmu-miR-466b-3-3p | 9.89 |
| mmu-miR-146a | 1.22 | mmu-miR-486 | 1.18 | mmu-miR-466f-5p | 9.50 |
| mmu-miR-30d | 1.22 | mmu-miR-92b | 1.18 | mmu-miR-297a* | 9.21 |
| mmu-miR-27a | 1.20 | mmu-miR-15b | 1.17 | mmu-miR-468 | 8.99 |
| mmu-miR-26a | 1.19 | mmu-miR-214 | 1.16 | mmu-miR-466a-3p | 8.48 |
| mmu-miR-30a | 1.18 | mmu-miR-26a | 1.08 | mmu-miR-197 | 8.06 |
| mmu-miR-15b | 1.14 | mmu-miR-27a | 0.93 | mmu-miR-455 | 7.51 |
| mmu-miR-23b | 1.09 | mmu-let-7g | 0.91 | mmu-miR-877* | 6.41 |
| mmu-miR-30c | 0.91 | mmu-let-7i | 0.89 | mmu-miR-297a | 6.38 |
| mmu-miR-143 | 0.87 | mmu-miR-451 | 0.84 | mmu-miR-15a* | 6.14 |
| mmu-miR-24 | 0.86 | mmu-miR-195 | 0.83 | mmu-miR-207 | 6.03 |
| mmu-miR-16 | 0.85 | mmu-miR-24 | 0.83 | mmu-miR-346 | 5.84 |
| mmu-let-7f | 0.84 | mmu-miR-30c | 0.81 | mmu-miR-466h | 5.54 |
| mmu-miR-195 | 0.84 | mmu-miR-30b | 0.80 | mmu-miR-206 | 5.48 |
| mmu-let-7d | 0.83 | mmu-miR-15a | 0.80 | mmu-miR-328 | 5.38 |
| mmu-miR-200c | 0.83 | mmu-miR-27b | 0.79 | mmu-miR-672 | 5.30 |
| mmu-miR-151-5p | 0.80 | mmu-miR-30a | 0.79 | mmu-miR-214 | 5.29 |
| mmu-miR-199a-3p | 0.80 | mmu-miR-143 | 0.77 | mmu-miR-320 | 4.52 |
| mmu-miR-145 | 0.78 | mmu-miR-29a | 0.77 | mmu-miR-34c* | 4.26 |
| mmu-miR-200b | 0.78 | mmu-miR-30e | 0.76 | mmu-miR-423-5p | 4.04 |
| mmu-miR-709 | 0.77 | mmu-miR-145 | 0.76 | mmu-miR-674 | 3.84 |
| mmu-let-7b | 0.75 | mmu-miR-574-5p | 0.76 | mmu-miR-151-3p | 3.18 |
| mmu-let-7e | 0.74 | mmu-miR-720 | 0.74 | mmu-miR-143 | 2.86 |
| mmu-miR-361 | 0.73 | mmu-miR-20a | 0.71 | mmu-miR-146b | 2.69 |
| mmu-miR-98 | 0.69 | mmu-miR-99a | 0.67 | mmu-miR-720 | 2.69 |
| mmu-miR-187 | 0.66 | mmu-miR-17 | 0.67 | mmu-miR-146a | 2.09 |
| mmu-miR-497 | 0.49 | mmu-miR-322 | 0.66 | mmu-miR-99b | 2.03 |
| mmu-miR-690 | 0.49 | mmu-miR-429 | 0.66 | mmu-miR-125b-5p | 1.88 |
| mmu-miR-1 | 0.46 | mmu-miR-199a-3p | 0.63 | mmu-miR-145 | 1.75 |
| mmu-miR-483 | 0.39 | mmu-miR-152 | 0.63 | mmu-miR-30d | 1.64 |
| mmu-miR-574-5p | 0.37 | mmu-miR-29c | 0.62 | mmu-miR-191 | 1.59 |
| mmu-miR-203 | 0.35 | mmu-miR-218 | 0.62 | mmu-miR-24 | 1.48 |
| mmu-miR-672 | 0.35 | mmu-miR-200a | 0.59 | mmu-let-7i | 1.42 |
| mmu-miR-805 | 0.28 | mmu-miR-10a | 0.59 | mmu-let-7f | 0.85 |
|  |  | mmu-miR-10b | 0.53 | mmu-let-7b | 0.83 |
|  |  | mmu-miR-29b | 0.52 | mmu-miR-29a | 0.76 |
|  |  | mmu-miR-101a | 0.36 | mmu-miR-126-3p | 0.74 |
|  |  | mmu-miR-223 | 0.32 | mmu-miR-200b | 0.61 |
|  |  | mmu-miR-19b | 0.31 | mmu-miR-92a | 0.60 |
|  |  | mmu-miR-690 | 0.24 | mmu-miR-30c | 0.59 |
|  |  | mmu-miR-450a-5p | 0.19 | mmu-miR-27a | 0.53 |
|  |  | mmu-miR-126-5p | 0.14 | mmu-let-7e | 0.50 |
|  |  |  |  | mmu-miR-21 | 0.47 |
|  |  |  |  | mmu-miR-25 | 0.46 |
|  |  |  |  | mmu-miR-30b | 0.44 |
|  |  |  |  | mmu-miR-23b | 0.41 |
|  |  |  |  | mmu-miR-23a | 0.38 |
|  |  |  |  | mmu-miR-26b | 0.31 |
|  |  |  |  | mmu-miR-98 | 0.20 |
|  |  |  |  | mmu-miR-15a | 0.19 |
|  |  |  |  | mmu-miR-29c | 0.11 |

Modulated mature miRNAs with a *p-value* < 0.01, FI: Fold Induction
