# Supplementary material for: MicroRNAs Profiling in Murine Models of Acute and Chronic Asthma: A Relationship with mRNAs Targets
Source: PLoS One. 2011 Jan 28;6(1):e16509. doi: 10.1371/journal.pone.0016509 (PMC3030602; doi:10.1371/journal.pone.0016509)
Supplement: Table S2 — Modulated mRNAs and inversely correlated modulated miRNAs at ST using MicroCosm Targets. (DOC) [file pone.0016509.s003.doc]

| **Modulated miRna at ST** | **FI** | **Target gene** | **FI** | **MicroCosm Targets**  ***p-value*** |
| --- | --- | --- | --- | --- |
| mmu-miR-712* | 5.24 | MBD1 | 0.66 | 0.00228546 |
| mmu-miR-122 | 5.06 | CSF3R | 0.39 | 3.82E-004 |
|  |  | IL6 | 0.47 | 0.00107067 |
|  |  | VLDLR | 0.59 | 0.00457035 |
| mmu-miR-181d | 2.51 | A930001N09RIK | 0.64 | 0.0024959 |
|  |  | CYR61 | 0.23 | 3.45E-004 |
|  |  | D4WSU53E | 0.47 | 0.0036682 |
|  |  | DLC1 | 0.44 | 7.98E-004 |
|  |  | FOXP1 | 0.52 | 3.17E-004 |
|  |  | HOD | 0.50 | 2.19E-005 |
|  |  | IL6 | 0.47 | 6.46E-005 |
|  |  | LEPR | 0.64 | 0.0016307 |
|  |  | RASSF8 | 0.57 | 4.77E-005 |
|  |  | SIPA1L2 | 0.59 | 0.00150953 |
|  |  | SLC40A1 | 0.59 | 6.44E-004 |
| mmu-miR-106a | 2.28 | D4WSU53E | 0.47 | 0.0017689 |
|  |  | DBP | 0.55 | 0.00207644 |
|  |  | ELK3 | 0.66 | 2.94E-005 |
|  |  | EPHB4 | 0.59 | 8.64E-004 |
|  |  | IRF2BP2 | 0.64 | 7.30E-006 |
|  |  | MAPRE1 | 0.62 | 0.00362909 |
|  |  | NFAT5 | 0.57 | 0.00431135 |
|  |  | RASSF8 | 0.57 | 0.00305061 |
|  |  | RORA | 0.50 | 4.52E-004 |
|  |  | SGIP1 | 0.64 | 6.09E-004 |
|  |  | SLC40A1 | 0.59 | 1.33E-004 |
|  |  | VLDLR | 0.59 | 0.00277353 |
| mmu-miR-223 | 1.99 | ARID4B | 0.64 | 9.24E-004 |
|  |  | LPIN2 | 0.57 | 0.00496082 |
|  |  | MYH10 | 0.54 | 0.0278464 |
|  |  | SCUBE2 | 0.52 | 2.10E-005 |
| mmu-miR-689 | 1.91 | DBP | 0.55 | 0.00240732 |
|  |  | EPHB4 | 0.59 | 2.10E-004 |
|  |  | GP1BB | 0.57 | 0.00460306 |
|  |  | HOXB5 | 0.55 | 0.00258136 |
|  |  | LIPE | 0.66 | 5.71E-004 |
|  |  | LPIN2 | 0.57 | 2.77E-004 |
|  |  | SKI | 0.66 | 0.00365195 |
| mmu-miR-181b | 1.91 | A930001N09RIK | 0.64 | 0.0024959 |
|  |  | D4WSU53E | 0.47 | 0.0036682 |
|  |  | FOXP1 | 0.52 | 3.17E-004 |
|  |  | HOD | 0.50 | 2.19E-005 |
|  |  | IL6 | 0.47 | 6.46E-005 |
|  |  | LEPR | 0.64 | 0.0016307 |
|  |  | RASSF8 | 0.57 | 4.77E-005 |
|  |  | SLC40A1 | 0.59 | 6.44E-004 |
|  |  | TCN2 | 0.50 | 0.00485041 |
| mmu-miR-146b | 1.91 | ALS2CL | 0.59 | 0.00348843 |
|  |  | AP4S1 | 0.57 | 0.0027992 |
|  |  | CARD10 | 0.55 | 2.89E-004 |
|  |  | CLEC4D | 0.39 | 0.00158968 |
|  |  | GPR116 | 0.57 | 0.00379661 |
|  |  | RASIP1 | 0.66 | 1.05E-005 |
|  |  | SCUBE2 | 0.52 | 1.94E-004 |
|  |  | TNFSF9 | 0.59 | 0.00207861 |
|  |  | UBR1 | 0.66 | 1.35E-004 |
|  |  | ZFP451 | 0.57 | 3.29E-004 |
| mmu-miR-20b | 1.88 | ARID4B | 0.64 | 2.79E-009 |
|  |  | D4WSU53E | 0.47 | 0.00112898 |
|  |  | DBP | 0.55 | 0.00473712 |
|  |  | ELK4 | 0.59 | 0.00328943 |
|  |  | MAPRE1 | 0.62 | 6.84E-004 |
|  |  | SLC40A1 | 0.59 | 1.20E-004 |
|  |  | VEGFA | 0.52 | 8.95E-004 |
|  |  | VLDLR | 0.59 | 5.36E-005 |
| mmu-miR-451 | 1.59 | CDKN1C | 0.59 | 0.0023708 |
|  |  | CENTD3 | 0.54 | 0.00389712 |
|  |  | ERG | 0.57 | 1.73E-005 |
|  |  | NPAL3 | 0.55 | 2.41E-004 |
|  |  | NR3C1 | 0.64 | 0.00149003 |
|  |  | SGIP1 | 0.64 | 6.02E-006 |
| mmu-miR-100 | 1.53 | ARHGEF18 | 0.64 | 8.29E-004 |
|  |  | TLR2 | 0.48 | 5.97E-005 |
| mmu-miR-187 | 0.66 | 1100001H23RIK | 1.62 | 0.00216149 |
|  |  | ATP2B2 | 2.00 | 0.00218136 |
|  |  | CAPG | 1.93 | 6.63E-005 |
|  |  | F7 | 2.00 | 2.43E-005 |
|  |  | H2AFX | 1.62 | 0.00370755 |
|  |  | MAFB | 3.48 | 0.00124494 |
|  |  | P2RY14 | 2.00 | 1.96E-004 |
|  |  | TBXAS1 | 1.87 | 0.00221293 |
|  |  | TMSB10 | 1.62 | 9.74E-004 |
|  |  | TNFAIP8L2 | 2.00 | 6.40E-004 |
| mmu-miR-690 | 0.49 | 5730536A07RIK | 1.74 | 0.00376481 |
|  |  | AURKA | 2.55 | 8.35E-004 |
|  |  | CIDEB | 1.93 | 0.00112531 |
|  |  | CXCL16 | 1.57 | 0.00437137 |
|  |  | FST | 1.87 | 7.76E-004 |
|  |  | TM4SF5 | 2.30 | 0.00312545 |
| mmu-miR-497 | 0.49 | 2310008M10RIK | 1.57 | 0.00138867 |
|  |  | COMMD9 | 1.74 | 0.00459539 |
|  |  | DDX39 | 1.52 | 2.23E-004 |
|  |  | KCNN4 | 1.87 | 1.71E-004 |
|  |  | NANS | 1.52 | 0.00112438 |
|  |  | SHCBP1 | 1.93 | 0.00115133 |
| mmu-miR-1 | 0.46 | ASPM | 2.73 | 1.75E-005 |
|  |  | CAPG | 1.93 | 0.00355117 |
|  |  | DOCK10 | 2.30 | 5.18E-004 |
|  |  | FBP1 | 4.00 | 4.37E-005 |
|  |  | MS4A7 | 3.73 | 0.00293356 |
|  |  | PSAT1 | 1.80 | 0.00493009 |
|  |  | SH3BGRL3 | 1.62 | 1.66E-004 |
| mmu-miR-483 | 0.39 | CD68 | 2.22 | 0.00219125 |
|  |  | F7 | 2.00 | 1.01E-004 |
|  |  | GPR65 | 2.38 | 2.78E-004 |
|  |  | MKI67 | 2.64 | 0.00140827 |
|  |  | PFDN1 | 1.57 | 0.00224953 |
|  |  | TACC3 | 2.30 | 0.00187266 |
|  |  | TIMP1 | 3.73 | 4.74E-004 |
|  |  | UBE2C | 2.46 | 0.00124363 |
| mmu-miR-574-5p | 0.37 | 9130211I03RIK | 3.86 | 0.00370092 |
|  |  | CAPG | 1.93 | 0.00387167 |
|  |  | CDCA8 | 3.03 | 5.99E-004 |
|  |  | D17WSU104E | 1.62 | 0.00194185 |
|  |  | MRPL54 | 1.52 | 0.00196901 |
|  |  | NUDT1 | 1.68 | 0.00104013 |
|  |  | OXCT1 | 1.68 | 0.00497954 |
|  |  | PRDX4 | 1.87 | 0.00141653 |
|  |  | REXO2 | 1.68 | 0.00264455 |
|  |  | SLPI | 4.59 | 0.00494159 |
|  |  | TNFSF13B | 1.68 | 1.73E-004 |
|  |  | TXNL2 | 1.62 | 0.00234016 |
|  |  | WAS | 1.62 | 0.00200928 |
| mmu-miR-672 | 0.35 | ADAM8 | 1.74 | 0.00218344 |
|  |  | CKLF | 1.87 | 0.00348343 |
|  |  | LY86 | 2.64 | 0.00212956 |
|  |  | PHB2 | 1.68 | 0.00421312 |
|  |  | SERPINA3N | 1.68 | 0.00134545 |
|  |  | SULF2 | 1.68 | 0.00463612 |
|  |  | TOP2A | 2.46 | 0.00365103 |
| mmu-miR-203 | 0.35 | AA467197 | 5.10 | 8.84E-004 |
|  |  | CYP7B1 | 2.30 | 8.03E-004 |
|  |  | PBK | 3.61 | 0.00368264 |
|  |  | SCIN | 2.55 | 0.018219 |
|  |  | TDO2 | 2.14 | 0.00214135 |
| mmu-miR-805 | 0.28 | CTSS | 1.93 | 6.27E-005 |
|  |  | CXCL16 | 1.57 | 0.00239309 |
|  |  | HEXB | 1.62 | 0.00121168 |
|  |  | IRGM | 1.62 | 4.66E-004 |
|  |  | OXCT1 | 1.68 | 0.00414127 |

Modulated mature miRNAs with a *p-value* < 0.005, FI: Fold Induction
