# Supplementary material for: MicroRNAs Profiling in Murine Models of Acute and Chronic Asthma: A Relationship with mRNAs Targets
Source: PLoS One. 2011 Jan 28;6(1):e16509. doi: 10.1371/journal.pone.0016509 (PMC3030602; doi:10.1371/journal.pone.0016509)
Supplement: Table S3 — Modulated mRNAs and inversely correlated modulated miRNAs at IT using MicroCosm Targets. (DOC) [file pone.0016509.s004.doc]

| **Modulated miRna at IT** | **FI** | **Target gene** | **FI** | **MicroCosm Targets**  ***p-value*** |
| --- | --- | --- | --- | --- |
| mmu-miR-155 | 4.47 | BAIAP2L1 | 0.66 | 5.28E-005 |
| mmu-miR-466g | 2.64 | HOXB5 | 0.57 | 1.39E-004 |
|  |  | MAPK10 | 0.25 | 6.98E-004 |
| mmu-miR-466f-3p | 2.23 | HIST2H3C2 | 0.52 | 8.68E-004 |
|  |  | LTF | 0.50 | 0.00286646 |
|  |  | TPCN1 | 0.64 | 2.70E-005 |
|  |  | VAT1 | 0.62 | 9.95E-004 |
| mmu-miR-455 | 2.03 | BCL9L | 0.57 | 0.0340771 |
|  |  | CENTD3 | 0.62 | 0.00116516 |
|  |  | GFRA2 | 0.50 | 3.66E-005 |
| mmu-miR-150 | 1.80 | CENTD3 | 0.62 | 0.00190938 |
|  |  | CSAD | 0.66 | 3.66E-004 |
|  |  | GFRA2 | 0.50 | 3.88E-004 |
| mmu-miR-423-5p | 1.69 | LTF | 0.50 | 0.00113467 |
| mmu-miR-146b | 1.56 | BAIAP2L1 | 0.66 | 4.48E-004 |
|  |  | PKP2 | 0.62 | 0.00100974 |
| mmu-miR-375 | 1.52 | MECP2 | 0.66 | 0.00194226 |
|  |  | POLR2A | 0.59 | 7.18E-004 |
| mmu-miR-322 | 0.66 | 6430527G18RIK | 2.07 | 2.53E-004 |
|  |  | AP3B1 | 2.14 | 0.0021635 |
|  |  | BC037112 | 4.59 | 4.09E-004 |
|  |  | CAB39 | 2.00 | 4.12E-005 |
|  |  | CCNT2 | 1.87 | 2.07E-004 |
|  |  | CD72 | 1.62 | 0.00411064 |
|  |  | EIF3S10 | 2.30 | 0.00302767 |
|  |  | GNA12 | 2.55 | 0.00108294 |
|  |  | ISOC1 | 2.55 | 0.00182257 |
|  |  | KIF1B | 1.52 | 0.00166905 |
|  |  | LRIG1 | 8.28 | 0.00822642 |
|  |  | RNF125 | 1.62 | 0.00169623 |
|  |  | SOS2 | 2.93 | 3.43E-005 |
|  |  | TCTA | 3.73 | 4.93E-004 |
|  |  | TOP2A | 2.38 | 0.00263857 |
| mmu-miR-429 | 0.66 | 2310047C04RIK | 3.48 | 6.14E-004 |
|  |  | ANGPTL2 | 1.93 | 4.75E-004 |
|  |  | APBB2 | 1.93 | 3.12E-004 |
|  |  | ARID5B | 1.80 | 1.64E-005 |
|  |  | ARIH1 | 1.93 | 5.58E-006 |
|  |  | B3GNT1 | 2.07 | 0.00130591 |
|  |  | BCAP29 | 1.52 | 1.73E-004 |
|  |  | BIRC5 | 2.38 | 7.98E-004 |
|  |  | CCNG2 | 2.00 | 0.00428737 |
|  |  | CLASP2 | 2.73 | 1.44E-005 |
|  |  | DDX46 | 1.87 | 5.98E-005 |
|  |  | DNAJC13 | 2.55 | 5.79E-004 |
|  |  | ERRFI1 | 1.57 | 1.49E-004 |
|  |  | FN1 | 1.93 | 1.38E-006 |
|  |  | FRMD4B | 2.00 | 7.23E-006 |
|  |  | FUBP1 | 1.93 | 4.46E-004 |
|  |  | GJA7 | 1.80 | 7.37E-004 |
|  |  | LATS2 | 4.29 | 1.61E-004 |
|  |  | LOX | 2.93 | 7.22E-005 |
|  |  | MAPRE1 | 2.38 | 6.74E-005 |
|  |  | MCM6 | 1.87 | 0.00251539 |
|  |  | PDIA4 | 1.80 | 0.00156468 |
|  |  | RASSF8 | 1.93 | 8.33E-005 |
|  |  | SLC39A6 | 1.62 | 2.74E-004 |
|  |  | SMURF2 | 2.00 | 1.77E-006 |
|  |  | SQLE | 1.62 | 0.00263316 |
|  |  | TCERG1 | 1.80 | 1.91E-006 |
|  |  | VLDLR | 2.14 | 0.00108622 |
|  |  | ZFR | 1.74 | 6.49E-004 |
| mmu-miR-152 | 0.63 | ABCB7 | 3.14 | 1.75E-007 |
|  |  | C1GALT1 | 2.83 | 6.47E-005 |
|  |  | CCNA2 | 3.03 | 0.00307572 |
|  |  | CCNB2 | 2.07 | 0.0023313 |
|  |  | CUL5 | 2.14 | 1.97E-004 |
|  |  | EAR11 | 5.46 | 0.00288076 |
|  |  | FBN1 | 2.30 | 1.05E-006 |
|  |  | FNBP4 | 1.93 | 0.00298011 |
|  |  | GLIPR2 | 2.46 | 1.08E-004 |
|  |  | HSD17B12 | 1.80 | 0.00128142 |
|  |  | MS4A7 | 2.07 | 0.00223765 |
|  |  | PAPOLA | 2.83 | 0.00282904 |
|  |  | RASSF8 | 1.93 | 2.57E-005 |
|  |  | RCC2 | 1.57 | 2.30E-005 |
|  |  | SFRS2IP | 2.64 | 3.36E-005 |
|  |  | SOS2 | 2.93 | 2.25E-007 |
|  |  | TMED7 | 2.64 | 0.00155557 |
|  |  | UBE2W | 1.68 | 0.00404835 |
|  |  | ZFP161 | 2.00 | 0.00250528 |
| mmu-miR-199a-3p | 0.63 | ARID4B | 19.70 | 3.65E-004 |
|  |  | CAB39 | 2.00 | 0.00279529 |
|  |  | CENTA2 | 2.38 | 6.70E-004 |
|  |  | DDHD2 | 2.38 | 2.20E-005 |
|  |  | FBLIM1 | 1.93 | 1.81E-004 |
|  |  | FBXW11 | 3.14 | 0.00259243 |
|  |  | GCLC | 1.80 | 0.00436786 |
|  |  | GJA7 | 1.80 | 0.00220312 |
|  |  | GNA12 | 2.55 | 1.54E-005 |
|  |  | JAK1 | 2.14 | 3.34E-004 |
|  |  | JARID1A | 4.29 | 2.26E-004 |
|  |  | MYO5A | 1.57 | 7.78E-004 |
|  |  | PPM1L | 1.62 | 4.57E-004 |
|  |  | SCAMP1 | 1.93 | 0.00245135 |
|  |  | SCYL1BP1 | 2.07 | 0.00257313 |
|  |  | SESN3 | 2.64 | 0.00126022 |
|  |  | SFRS8 | 3.03 | 6.77E-004 |
|  |  | TMOD2 | 1.93 | 0.00360147 |
| mmu-miR-218 | 0.62 | APBB2 | 1.93 | 1.68E-005 |
|  |  | ARAF | 1.68 | 0.00238438 |
|  |  | ARID4B | 19.70 | 2.68E-005 |
|  |  | GNAS | 1.87 | 0.0120314 |
|  |  | PDE7A | 1.80 | 0.00132983 |
|  |  | PHF3 | 3.73 | 0.00109395 |
|  |  | PKN2 | 2.30 | 0.00509315 |
|  |  | SEC61A1 | 2.83 | 1.77E-004 |
|  |  | TFPI | 1.68 | 0.00196996 |
| mmu-miR-29c | 0.62 | 1810073N04RIK | 2.00 | 0.0296083 |
|  |  | A230046K03RIK | 1.87 | 7.46E-004 |
|  |  | AP3B1 | 2.14 | 0.00417875 |
|  |  | ARHGAP29 | 5.86 | 0.00467076 |
|  |  | BACH2 | 1.74 | 1.54E-007 |
|  |  | CCNA2 | 3.03 | 6.58E-005 |
|  |  | CCNL2 | 1.68 | 3.63E-005 |
|  |  | CDCA3 | 1.87 | 5.89E-004 |
|  |  | COL4A5 | 1.68 | 7.70E-005 |
|  |  | COL6A2 | 1.80 | 4.48E-009 |
|  |  | CTNNB1 | 1.93 | 0.00158884 |
|  |  | CTSK | 1.87 | 6.88E-004 |
|  |  | DGKD | 2.22 | 8.28E-008 |
|  |  | DNMT3A | 2.14 | 5.53E-006 |
|  |  | DYRK1A | 2.30 | 2.43E-004 |
|  |  | ELF2 | 1.87 | 6.84E-006 |
|  |  | FBN1 | 2.30 | 5.99E-006 |
|  |  | GAB1 | 2.00 | 1.39E-005 |
|  |  | HBP1 | 1.74 | 4.65E-008 |
|  |  | MAPK8 | 8.57 | 0.00132099 |
|  |  | METRNL | 2.30 | 0.00412566 |
|  |  | PIK3R1 | 2.38 | 5.15E-006 |
|  |  | RNF19 | 2.00 | 0.00481089 |
|  |  | SLC7A6 | 1.57 | 6.71E-004 |
|  |  | TCEA1 | 2.46 | 8.16E-004 |
|  |  | UNC84A | 1.87 | 0.00464242 |
|  |  | USP22 | 2.38 | 3.14E-006 |
|  |  | USP34 | 1.74 | 0.00205923 |
|  |  | WARS | 1.62 | 0.00196394 |
|  |  | ZFP445 | 2.64 | 0.00343998 |
| mmu-miR-10a | 0.59 | ALDH2 | 1.80 | 5.61E-005 |
|  |  | ITGB1 | 1.74 | 0.00214049 |
|  |  | POLR3C | 1.68 | 0.00207812 |
| mmu-miR-200a | 0.59 | 6430527G18RIK | 2.07 | 0.00151478 |
|  |  | ABI2 | 1.74 | 3.72E-007 |
|  |  | AKAP13 | 1.80 | 0.00243795 |
|  |  | ANKRD25 | 3.03 | 5.34E-004 |
|  |  | APBB2 | 1.93 | 4.58E-004 |
|  |  | API5 | 2.30 | 1.00E-005 |
|  |  | ARID4B | 19.70 | 0.00133228 |
|  |  | ATE1 | 8.28 | 0.00199144 |
|  |  | ATP11C | 2.93 | 2.47E-004 |
|  |  | ATP8A1 | 3.61 | 3.29E-004 |
|  |  | B3GNT1 | 2.07 | 6.88E-004 |
|  |  | CD72 | 1.62 | 2.27E-004 |
|  |  | CLASP2 | 2.73 | 2.01E-008 |
|  |  | CP | 4.14 | 1.23E-004 |
|  |  | DAPK1 | 1.74 | 0.00745181 |
|  |  | DDX46 | 1.87 | 0.0334205 |
|  |  | DNAJB4 | 1.93 | 3.05E-004 |
|  |  | DNAJC13 | 2.55 | 1.51E-004 |
|  |  | DR1 | 2.14 | 3.16E-005 |
|  |  | FN1 | 1.93 | 9.83E-007 |
|  |  | GJA7 | 1.80 | 1.07E-005 |
|  |  | GMFB | 3.61 | 0.00368222 |
|  |  | GULP1 | 5.10 | 1.92E-005 |
|  |  | HNRPU | 1.52 | 0.00365466 |
|  |  | IFNAR1 | 1.74 | 0.00398427 |
|  |  | IGF2R | 2.07 | 0.00417467 |
|  |  | LANCL2 | 2.93 | 0.00337065 |
|  |  | LOX | 2.93 | 9.54E-005 |
|  |  | MCM6 | 1.87 | 6.68E-004 |
|  |  | MS4A7 | 2.07 | 0.00343283 |
|  |  | MTMR6 | 1.93 | 0.003057 |
|  |  | NAB1 | 1.57 | 1.92E-004 |
|  |  | NEBL | 7.73 | 0.00355411 |
|  |  | PSAT1 | 1.74 | 9.20E-006 |
|  |  | RAPGEF5 | 1.80 | 7.59E-004 |
|  |  | RASSF8 | 1.93 | 0.00276772 |
|  |  | SLC6A14 | 2.00 | 4.07E-004 |
|  |  | ST3GAL4 | 2.00 | 0.00108983 |
|  |  | SUZ12 | 1.80 | 0.00380205 |
|  |  | TCERG1 | 1.80 | 7.31E-007 |
|  |  | ZFP644 | 1.62 | 5.05E-004 |
|  |  | ZFR | 1.74 | 2.26E-005 |
| mmu-miR-10b | 0.53 | DNM2 | 2.30 | 6.27E-004 |
|  |  | ITGB1 | 1.74 | 0.00214049 |
|  |  | MBNL2 | 1.74 | 0.00489747 |
|  |  | POLR3C | 1.68 | 0.00207812 |
|  |  | RORA | 2.38 | 6.08E-004 |
|  |  | ZFP644 | 1.62 | 7.19E-004 |
|  |  | ZMYND11 | 1.87 | 1.16E-005 |
| mmu-miR-29b | 0.52 | 1810073N04RIK | 2.00 | 0.0264724 |
|  |  | A430093A21RIK | 2.07 | 2.64E-006 |
|  |  | ABLIM1 | 1.93 | 7.24E-004 |
|  |  | AP3B1 | 2.14 | 0.00417875 |
|  |  | ARHGAP29 | 5.86 | 0.00467076 |
|  |  | BACH2 | 1.74 | 1.54E-007 |
|  |  | BRD1 | 1.80 | 0.00393363 |
|  |  | CCNA2 | 3.03 | 6.58E-005 |
|  |  | CCNL2 | 1.68 | 3.63E-005 |
|  |  | CDCA3 | 1.87 | 5.89E-004 |
|  |  | COL4A5 | 1.68 | 7.70E-005 |
|  |  | COL6A2 | 1.80 | 4.48E-009 |
|  |  | CTNNB1 | 1.93 | 0.00158884 |
|  |  | CTSK | 1.87 | 6.88E-004 |
|  |  | DGKD | 2.22 | 8.28E-008 |
|  |  | DYRK1A | 2.30 | 2.43E-004 |
|  |  | ELF2 | 1.87 | 6.84E-006 |
|  |  | FBN1 | 2.30 | 5.99E-006 |
|  |  | GAB1 | 2.00 | 1.39E-005 |
|  |  | HBP1 | 1.74 | 4.65E-008 |
|  |  | MAPK8 | 8.57 | 0.00132099 |
|  |  | METRNL | 2.30 | 0.00412566 |
|  |  | OTUD4 | 1.62 | 0.00269145 |
|  |  | PDGFRA | 3.61 | 0.00428249 |
|  |  | PIK3R1 | 2.38 | 5.15E-006 |
|  |  | TCEA1 | 2.46 | 8.16E-004 |
|  |  | UNC84A | 1.87 | 0.00464242 |
|  |  | USP22 | 2.38 | 3.14E-006 |
|  |  | USP34 | 1.74 | 0.00205923 |
|  |  | WARS | 1.62 | 0.00196394 |
| mmu-miR-101a | 0.36 | ARCN1 | 2.38 | 0.00181306 |
|  |  | ASPN | 2.22 | 0.00272071 |
|  |  | BCLAF1 | 3.73 | 9.19E-004 |
|  |  | CCNT2 | 1.87 | 9.78E-004 |
|  |  | CD2AP | 2.30 | 9.53E-004 |
|  |  | DDX46 | 1.87 | 0.026005 |
|  |  | FGFR3 | 3.61 | 0.00375306 |
|  |  | FOXP1 | 1.93 | 0.00134763 |
|  |  | GJA1 | 5.10 | 1.66E-005 |
|  |  | GULP1 | 5.10 | 7.28E-004 |
|  |  | KLF6 | 2.30 | 0.00383708 |
|  |  | MAGI1 | 2.83 | 0.00171527 |
|  |  | MRC1 | 1.74 | 7.29E-004 |
|  |  | NPNT | 1.74 | 6.90E-004 |
|  |  | ORC4L | 1.80 | 3.43E-004 |
|  |  | PKN2 | 2.30 | 0.00236261 |
|  |  | RNF25 | 2.22 | 1.65E-005 |
|  |  | RTN4 | 1.93 | 6.10E-004 |
|  |  | SCAMP1 | 1.93 | 0.00293982 |
|  |  | SLC12A2 | 2.14 | 3.28E-004 |
|  |  | SLC12A7 | 1.93 | 0.00413613 |
|  |  | SLC39A6 | 1.62 | 0.00146593 |
|  |  | TFPI2 | 2.46 | 0.00141731 |
|  |  | TMED5 | 1.93 | 8.33E-004 |
|  |  | TOP2A | 2.38 | 0.00223082 |
|  |  | UHMK1 | 2.73 | 3.92E-004 |
|  |  | VPS35 | 2.93 | 4.75E-004 |
|  |  | WDR35 | 3.61 | 4.89E-004 |
|  |  | YTHDF3 | 1.93 | 0.00266108 |
| mmu-miR-223 | 0.32 | 5730596K20RIK | 3.14 | 0.00326304 |
|  |  | ADAMTS9 | 1.80 | 0.00380809 |
|  |  | ALCAM | 1.57 | 0.00327714 |
|  |  | ARID4B | 19.70 | 9.24E-004 |
|  |  | CACNA2D1 | 2.64 | 0.00216334 |
|  |  | GPAM | 2.83 | 0.00766118 |
|  |  | LPIN2 | 2.14 | 0.00496082 |
|  |  | MTM1 | 3.14 | 0.00337633 |
|  |  | MYO5A | 1.57 | 1.91E-004 |
|  |  | NPNT | 1.74 | 0.00124897 |
|  |  | PHF3 | 3.73 | 0.00332717 |
|  |  | RASA1 | 2.07 | 8.58E-005 |
|  |  | SBF2 | 1.62 | 3.87E-004 |
|  |  | TCERG1 | 1.80 | 1.58E-004 |
| mmu-miR-19b | 0.31 | 2310035C23RIK | 1.80 | 8.03E-004 |
|  |  | ABCB7 | 3.14 | 0.0078984 |
|  |  | ANGPT1 | 3.25 | 0.0038333 |
|  |  | ARMC8 | 1.74 | 1.95E-004 |
|  |  | ASAHL | 1.57 | 0.00279309 |
|  |  | BTBD7 | 2.07 | 0.00439766 |
|  |  | CCNB2 | 2.07 | 4.66E-005 |
|  |  | CCNC | 7.73 | 6.73E-004 |
|  |  | CILP | 1.62 | 1.77E-005 |
|  |  | CLASP2 | 2.73 | 0.0014804 |
|  |  | CNOT4 | 2.22 | 0.00153417 |
|  |  | CPD | 3.73 | 0.00153308 |
|  |  | D4WSU53E | 1.93 | 0.00219988 |
|  |  | DLC1 | 6.28 | 0.00170748 |
|  |  | EAR11 | 5.46 | 0.00109749 |
|  |  | GCLC | 1.80 | 0.00269992 |
|  |  | GJA7 | 1.80 | 0.00197008 |
|  |  | GNAS | 1.87 | 0.00424019 |
|  |  | HIP1 | 2.30 | 0.0021145 |
|  |  | IVNS1ABP | 2.30 | 1.15E-005 |
|  |  | LATS1 | 2.38 | 0.0023539 |
|  |  | MAPK6 | 1.52 | 3.77E-005 |
|  |  | MYH11 | 1.57 | 5.21E-004 |
|  |  | NBEAL2 | 2.22 | 0.0139907 |
|  |  | PDGFRA | 3.61 | 0.00131607 |
|  |  | PTPRG | 1.68 | 5.52E-008 |
|  |  | RBM5 | 2.00 | 2.56E-004 |
|  |  | RORA | 2.38 | 0.00358009 |
|  |  | SLC39A10 | 2.55 | 2.12E-005 |
|  |  | SRRM1 | 1.80 | 3.74E-005 |
|  |  | TNFRSF12A | 1.74 | 0.00153933 |
|  |  | WHSC1L1 | 2.55 | 0.00380151 |
| mmu-miR-690 | 0.24 | BBX | 2.93 | 0.00335663 |
|  |  | CPD | 3.73 | 0.00122936 |
|  |  | CTNNB1 | 1.93 | 4.65E-004 |
|  |  | FLT1 | 2.38 | 0.00301485 |
|  |  | FST | 2.64 | 7.76E-004 |
|  |  | MT2 | 2.00 | 8.82E-004 |
|  |  | RBM5 | 2.00 | 0.00336804 |
|  |  | SHC1 | 1.74 | 0.0442264 |
|  |  | TBC1D14 | 2.22 | 8.42E-007 |
|  |  | VIPR2 | 2.22 | 0.00287376 |
| mmu-miR-450a-5p | 0.19 | 1810013L24RIK | 1.68 | 0.0043935 |
|  |  | 2810407C02RIK | 1.68 | 0.00146286 |
|  |  | 9430010O03RIK | 4.00 | 0.00425345 |
|  |  | AFF1 | 6.28 | 3.79E-004 |
|  |  | DDX46 | 1.87 | 0.00179745 |
|  |  | DYRK1A | 2.30 | 3.90E-004 |
|  |  | FCGR2B | 2.73 | 0.00318171 |
|  |  | FRK | 3.03 | 2.56E-005 |
|  |  | GPBP1 | 2.73 | 0.00180486 |
|  |  | GSPT1 | 1.74 | 6.38E-005 |
|  |  | HNRPU | 1.52 | 0.00357063 |
|  |  | IGF1 | 3.61 | 0.00351119 |
|  |  | JAM2 | 2.22 | 0.00471269 |
|  |  | LATS1 | 2.38 | 9.45E-004 |
|  |  | LYPLA1 | 2.93 | 2.03E-004 |
|  |  | MAK10 | 1.87 | 0.00178957 |
|  |  | MAN1A | 2.14 | 1.26E-006 |
|  |  | MEF2A | 12.13 | 0.00318758 |
|  |  | MMP12 | 3.36 | 0.00179745 |
|  |  | PLXDC2 | 1.62 | 1.49E-004 |
|  |  | PLXNC1 | 9.19 | 0.00687778 |
|  |  | PNN | 2.64 | 2.18E-004 |
|  |  | SESN3 | 2.64 | 4.15E-004 |
|  |  | SLK | 2.55 | 0.00345596 |
|  |  | TCERG1 | 1.80 | 9.13E-005 |
|  |  | TOP2A | 2.38 | 0.00179253 |
| mmu-miR-126-5p | 0.14 | 2310035C23RIK | 1.80 | 0.00231139 |
|  |  | AP3B1 | 2.14 | 0.0027776 |
|  |  | BCAP29 | 1.52 | 0.00218895 |
|  |  | C030011O14RIK | 2.64 | 0.00240329 |
|  |  | C920006C10RIK | 2.64 | 3.25E-004 |
|  |  | COL4A5 | 1.68 | 0.00464663 |
|  |  | D16ERTD472E | 1.80 | 0.00458511 |
|  |  | GCLC | 1.80 | 2.46E-004 |
|  |  | GDF10 | 1.80 | 7.75E-005 |
|  |  | ITGA2 | 2.64 | 1.43E-004 |
|  |  | ITGB6 | 2.73 | 0.00287094 |
|  |  | MAP3K7 | 1.93 | 5.80E-004 |
|  |  | MRC1 | 1.74 | 0.00307447 |
|  |  | NCOA4 | 2.22 | 0.00323938 |
|  |  | NUDT4 | 1.68 | 0.00395712 |
|  |  | TTC14 | 2.00 | 9.09E-005 |
|  |  | XRN2 | 2.14 | 0.00448354 |

Modulated mature miRNAs with a *p-value* < 0.005, FI: Fold Induction
