# Supplementary material for: MicroRNAs Profiling in Murine Models of Acute and Chronic Asthma: A Relationship with mRNAs Targets
Source: PLoS One. 2011 Jan 28;6(1):e16509. doi: 10.1371/journal.pone.0016509 (PMC3030602; doi:10.1371/journal.pone.0016509)
Supplement: Table S4 — Modulated mRNAs and inversely correlated modulated miRNAs at using MicroCosm Targets. (DOC) [file pone.0016509.s005.doc]

| **Modulated miRna at LT** | **FI** | **Target gene** | **FI** | **MicroCosm Targets**  ***p-value*** |
| --- | --- | --- | --- | --- |
| mmu-miR-705 | 119.43 | DDX39 | 0.57 | 7.03E-004 |
|  |  | MCM2 | 0.57 | 0.00330521 |
|  |  | MCM3 | 0.57 | 7.12E-004 |
|  |  | XRCC6 | 0.11 | 1.91E-004 |
| mmu-miR-188-5p | 117.78 | REXO4 | 0.64 | 0.00212746 |
| mmu-miR-483 | 116.16 | LY6E | 0.55 | 2.84E-004 |
|  |  | MKI67 | 0.45 | 0.00140827 |
|  |  | UBE2C | 0.39 | 0.00124363 |
|  |  | UBE3C | 0.44 | 8.19E-004 |
| mmu-miR-669c | 115.36 | HMGB2 | 0.34 | 0.00291674 |
|  |  | MCM3 | 0.57 | 4.02E-004 |
|  |  | MYC | 0.45 | 0.00136396 |
| mmu-miR-568 | 96.34 | D11ERTD497E | 0.57 | 9.34E-004 |
|  |  | NIN | 0.55 | 0.0432654 |
|  |  | NOL5 | 0.52 | 0.00324405 |
| mmu-miR-467b* | 48.50 | AURKA | 0.52 | 1.50E-004 |
| mmu-miR-691 | 42.52 | EZH2 | 0.35 | 0.00151528 |
|  |  | KTI12 | 0.55 | 0.00363471 |
|  |  | MTF2 | 0.62 | 8.29E-004 |
| mmu-miR-671-5p | 39.67 | BUB1B | 0.39 | 0.00240807 |
|  |  | C1QBP | 0.66 | 0.0014938 |
|  |  | MCM6 | 0.47 | 0.00297952 |
|  |  | TSSC1 | 0.52 | 0.00154276 |
| mmu-miR-467a* | 39.12 | AURKA | 0.52 | 1.50E-004 |
| mmu-miR-485* | 33.36 | CD3G | 0.32 | 0.00229376 |
|  |  | MCM3 | 0.57 | 0.00216194 |
| mmu-miR-744 | 29.65 | GTF2H4 | 0.31 | 0.00382077 |
|  |  | ZAP70 | 0.39 | 6.45E-004 |
| mmu-miR-466f-3p | 22.63 | 4631424J17RIK | 0.62 | 1.00E-006 |
|  |  | AURKA | 0.52 | 6.33E-005 |
|  |  | CBX1 | 0.57 | 0.00407737 |
|  |  | CCNB2 | 0.22 | 1.00E-006 |
|  |  | CD6 | 0.54 | 1.00E-006 |
|  |  | CDC7 | 0.45 | 2.59E-004 |
|  |  | CDCA3 | 0.44 | 1.35E-004 |
|  |  | GMNN | 0.55 | 8.49E-005 |
|  |  | KIF11 | 0.45 | 6.85E-005 |
|  |  | LCK | 0.30 | 8.37E-009 |
|  |  | MCM3 | 0.57 | 8.61E-004 |
|  |  | MTF2 | 0.62 | 1.88E-005 |
|  |  | PLK4 | 0.52 | 2.11E-008 |
|  |  | TMPO | 0.59 | 0.00435753 |
| mmu-miR-685 | 19.56 | C1QBP | 0.66 | 0.0021573 |
|  |  | CCNB2 | 0.22 | 0.00175737 |
|  |  | COX5A | 0.59 | 0.00470988 |
|  |  | GIMAP9 | 0.41 | 0.00109152 |
|  |  | H2AFZ | 0.66 | 0.00154589 |
|  |  | NNT | 0.59 | 0.00369042 |
|  |  | RAD51 | 0.54 | 8.54E-004 |
|  |  | SLC35A5 | 0.55 | 0.00450505 |
|  |  | TTRAP | 0.62 | 0.00396548 |
| mmu-miR-709 | 19.03 | MCM5 | 0.57 | 2.06E-007 |
| mmu-miR-467e* | 18.51 | AURKA | 0.52 | 1.50E-004 |
| mmu-miR-466c-5p | 17.03 | 4631424J17RIK | 0.62 | 1.00E-006 |
|  |  | CAMP | 0.30 | 0.00178086 |
|  |  | CD6 | 0.54 | 1.00E-006 |
|  |  | CDCA8 | 0.35 | 0.00307543 |
|  |  | DTX1 | 0.32 | 3.24E-007 |
|  |  | HSP110 | 0.55 | 4.89E-004 |
|  |  | LCK | 0.30 | 8.37E-009 |
|  |  | MTF2 | 0.62 | 1.88E-005 |
|  |  | MTM1 | 0.52 | 2.66E-005 |
|  |  | NIN | 0.55 | 1.00E-006 |
|  |  | NOLA2 | 0.55 | 0.00124285 |
|  |  | SLC43A1 | 0.50 | 9.51E-006 |
|  |  | TMPO | 0.59 | 0.00435753 |
| mmu-miR-466g | 16.22 | 4631424J17RIK | 0.62 | 0.00330797 |
|  |  | ATPIF1 | 0.62 | 2.47E-004 |
|  |  | BCL11B | 0.26 | 0.0267009 |
|  |  | BIRC5 | 0.29 | 1.48E-004 |
|  |  | CCNB2 | 0.22 | 1.00E-006 |
|  |  | CD6 | 0.54 | 1.00E-006 |
|  |  | MCM5 | 0.57 | 0.00177858 |
|  |  | MCM6 | 0.47 | 9.31E-004 |
|  |  | PLK4 | 0.52 | 2.11E-008 |
| mmu-miR-574-3p | 16.00 | 2610018G03RIK | 0.54 | 0.00176124 |
|  |  | CCT2 | 0.57 | 0.0030884 |
|  |  | MCM5 | 0.57 | 0.00462822 |
|  |  | NOLA2 | 0.55 | 8.58E-004 |
|  |  | PHB2 | 0.66 | 0.00166477 |
|  |  | PRR6 | 0.54 | 0.00190363 |
| mmu-miR-574-5p | 13.18 | CDCA8 | 0.35 | 5.99E-004 |
|  |  | DTX1 | 0.32 | 4.83E-004 |
| mmu-miR-667 | 13.18 | GTF2E2 | 0.62 | 0.0348136 |
| mmu-miR-713 | 11.79 | 2700029M09RIK | 0.64 | 0.00116955 |
|  |  | 5830411N06RIK | 0.45 | 0.00207554 |
|  |  | CDCA2 | 0.41 | 9.31E-004 |
|  |  | NUCKS1 | 0.54 | 9.32E-004 |
|  |  | PRPF4B | 0.54 | 1.49E-004 |
| mmu-let-7d* | 11.71 | BXDC1 | 0.66 | 0.00109055 |
|  |  | C1QBP | 0.66 | 9.08E-004 |
|  |  | HMGB2 | 0.34 | 6.85E-005 |
|  |  | HSP110 | 0.55 | 0.00339352 |
|  |  | NASP | 0.62 | 0.00387139 |
|  |  | NIN | 0.55 | 0.00434564 |
|  |  | PRR6 | 0.54 | 0.00111737 |
|  |  | RAN | 0.59 | 0.00267945 |
|  |  | SLC25A5 | 0.59 | 0.00286304 |
| mmu-miR-762 | 11.24 | CD3E | 0.42 | 1.08E-004 |
|  |  | DDX39 | 0.57 | 0.00333945 |
|  |  | MCM3 | 0.57 | 0.00142969 |
|  |  | MCM6 | 0.47 | 8.09E-004 |
| mmu-miR-466b-3-3p | 9.92 | 4631424J17RIK | 0.62 | 1.00E-006 |
|  |  | AURKA | 0.52 | 6.33E-005 |
|  |  | BIRC5 | 0.29 | 1.48E-004 |
|  |  | BUB1 | 0.22 | 0.00236536 |
|  |  | CBX1 | 0.57 | 0.00407737 |
|  |  | CCNB2 | 0.22 | 1.00E-006 |
|  |  | CD6 | 0.54 | 1.00E-006 |
|  |  | CDC7 | 0.45 | 2.59E-004 |
|  |  | CENPC1 | 0.48 | 1.08E-004 |
|  |  | GATA3 | 0.44 | 0.00273465 |
|  |  | GMNN | 0.55 | 8.49E-005 |
|  |  | MCM3 | 0.57 | 8.61E-004 |
|  |  | NOL5 | 0.52 | 2.60E-004 |
|  |  | TPX2 | 0.37 | 8.31E-006 |
|  |  | UBE3C | 0.44 | 0.00122279 |
| mmu-miR-466d-3p | 9.92 | 4631424J17RIK | 0.62 | 1.00E-006 |
|  |  | AURKA | 0.52 | 6.33E-005 |
|  |  | BIRC5 | 0.29 | 1.48E-004 |
|  |  | BUB1 | 0.22 | 0.0493025 |
|  |  | CCNB2 | 0.22 | 1.00E-006 |
|  |  | CD6 | 0.54 | 1.00E-006 |
|  |  | CDC7 | 0.45 | 2.59E-004 |
|  |  | CENPC1 | 0.48 | 1.08E-004 |
|  |  | DDX39 | 0.57 | 0.00125405 |
|  |  | GATA3 | 0.44 | 0.00273465 |
|  |  | MCM3 | 0.57 | 8.61E-004 |
|  |  | MTF2 | 0.62 | 1.88E-005 |
|  |  | NOL5 | 0.52 | 2.60E-004 |
|  |  | TPX2 | 0.37 | 8.31E-006 |
| mmu-miR-466f-5p | 9.51 | 4631424J17RIK | 0.62 | 1.00E-006 |
|  |  | BCL11B | 0.26 | 0.00182738 |
|  |  | CD6 | 0.54 | 1.00E-006 |
|  |  | CDCA8 | 0.35 | 0.00307543 |
|  |  | DTX1 | 0.32 | 3.24E-007 |
|  |  | LCK | 0.30 | 8.37E-009 |
|  |  | MCM6 | 0.47 | 9.31E-004 |
|  |  | MTF2 | 0.62 | 1.88E-005 |
|  |  | NIN | 0.55 | 1.00E-006 |
|  |  | PON1 | 0.59 | 0.00488942 |
|  |  | PYCARD | 0.47 | 8.20E-004 |
|  |  | T | 0.00 | 6.20E-005 |
|  |  | TPX2 | 0.37 | 8.31E-006 |
| mmu-miR-297a* | 9.19 | AURKA | 0.52 | 0.00297754 |
|  |  | CCNB2 | 0.22 | 0.00190437 |
|  |  | NOL5 | 0.52 | 0.00217924 |
| mmu-miR-468 | 9.00 | HELLS | 0.45 | 6.22E-007 |
|  |  | TOP2A | 0.29 | 0.00184825 |
| mmu-miR-466a-3p | 8.46 | 4631424J17RIK | 0.62 | 1.00E-006 |
|  |  | AURKA | 0.52 | 6.33E-005 |
|  |  | BIRC5 | 0.29 | 1.48E-004 |
|  |  | BUB1 | 0.22 | 0.04944 |
|  |  | C1QBP | 0.66 | 4.16E-004 |
|  |  | CCNB2 | 0.22 | 1.00E-006 |
|  |  | CD6 | 0.54 | 1.00E-006 |
|  |  | CDC7 | 0.45 | 2.59E-004 |
|  |  | CENPC1 | 0.48 | 1.08E-004 |
|  |  | DDX39 | 0.57 | 0.00125405 |
|  |  | GATA3 | 0.44 | 0.00273465 |
|  |  | MCM3 | 0.57 | 8.61E-004 |
|  |  | NOL5 | 0.52 | 2.60E-004 |
|  |  | TPX2 | 0.37 | 8.31E-006 |
| mmu-miR-197 | 8.06 | DENND2D | 0.47 | 8.71E-004 |
| mmu-miR-455 | 7.52 | H2AFX | 0.45 | 2.59E-004 |
|  |  | H2AFZ | 0.66 | 0.00498934 |
|  |  | HDAC2 | 0.50 | 0.00418774 |
|  |  | NNT | 0.59 | 3.53E-004 |
|  |  | RRS1 | 0.54 | 0.00339709 |
|  |  | ZAP70 | 0.39 | 7.83E-004 |
| mmu-miR-297a | 6.36 | D15WSU75E | 0.45 | 0.00338797 |
|  |  | GIMAP9 | 0.41 | 0.00316627 |
|  |  | MTF2 | 0.62 | 0.00360443 |
|  |  | SLC43A1 | 0.50 | 2.00E-004 |
| mmu-miR-15a* | 6.15 | CCNB2 | 0.22 | 0.00278249 |
|  |  | LCK | 0.30 | 0.00148802 |
| mmu-miR-207 | 6.02 | LCK | 0.30 | 5.79E-004 |
| mmu-miR-346 | 5.86 | MCM3 | 0.57 | 0.0018659 |
| mmu-miR-466h | 5.54 | CAMP | 0.30 | 0.00178086 |
|  |  | CD2 | 0.50 | 0.00141905 |
|  |  | CD6 | 0.54 | 1.00E-006 |
|  |  | CDCA2 | 0.41 | 0.00243299 |
|  |  | CDCA8 | 0.35 | 0.00307543 |
|  |  | LCK | 0.30 | 8.37E-009 |
|  |  | NIN | 0.55 | 1.00E-006 |
|  |  | NOLA2 | 0.55 | 0.00124285 |
|  |  | PON1 | 0.59 | 0.00488942 |
|  |  | RPL13A | 0.54 | 0.00350922 |
|  |  | SLC43A1 | 0.50 | 9.51E-006 |
|  |  | UCHL5 | 0.62 | 5.34E-006 |
|  |  | WDR12 | 0.59 | 0.00319392 |
| mmu-miR-206 | 5.46 | AI586015 | 0.45 | 0.00166143 |
|  |  | NUP160 | 0.62 | 7.12E-004 |
|  |  | RASGRP1 | 0.55 | 0.0241543 |
|  |  | ZFP647 | 0.62 | 0.00227073 |
| mmu-miR-672 | 5.31 | MYC | 0.45 | 6.76E-005 |
|  |  | PHB2 | 0.66 | 0.00421312 |
|  |  | TOP2A | 0.29 | 0.00365103 |
| mmu-miR-214 | 5.28 | BUB1B | 0.39 | 0.00457919 |
|  |  | DDX39 | 0.57 | 0.00286032 |
|  |  | ITM2A | 0.54 | 0.00426815 |
|  |  | NGP | 0.29 | 0.00364866 |
|  |  | PSAT1 | 0.47 | 1.15E-004 |
|  |  | RCC2 | 0.62 | 0.00439248 |
| mmu-miR-320 | 4.53 | KIF22 | 0.29 | 1.25E-004 |
|  |  | PRR6 | 0.54 | 8.41E-005 |
| mmu-miR-423-5p | 4.03 | GTF2E2 | 0.62 | 0.0293515 |
|  |  | HSP110 | 0.55 | 0.00108586 |
| mmu-miR-674 | 3.84 | CDCA2 | 0.41 | 3.36E-004 |
|  |  | TAF1C | 0.62 | 4.78E-005 |
| mmu-miR-151-3p | 3.18 | CBX1 | 0.57 | 0.00206601 |
|  |  | GTF2E2 | 0.62 | 0.017358 |
|  |  | HEMGN | 0.16 | 0.00286939 |
|  |  | MGEA6 | 0.55 | 0.00450962 |
|  |  | REXO4 | 0.64 | 0.00493498 |
| mmu-miR-143 | 2.87 | AI586015 | 0.45 | 0.00237462 |
|  |  | ARHGAP15 | 0.59 | 0.00207925 |
|  |  | SLC25A5 | 0.59 | 0.00452074 |
|  |  | TOP2A | 0.29 | 7.25E-004 |
| mmu-miR-146b | 2.69 | HORMAD1 | 0.47 | 0.00464978 |
|  |  | KIF22 | 0.29 | 0.00363725 |
|  |  | MGEA6 | 0.55 | 0.0230102 |
|  |  | RPS9 | 0.62 | 1.01E-004 |
| mmu-miR-720 | 2.69 | RACGAP1 | 0.57 | 0.00482628 |
| mmu-miR-146a | 2.10 | KIF22 | 0.29 | 0.00363725 |
|  |  | MGEA6 | 0.55 | 0.0465236 |
|  |  | RPS9 | 0.62 | 1.01E-004 |
| mmu-miR-99b | 2.03 | BIRC5 | 0.29 | 0.00444786 |
|  |  | LCK | 0.30 | 0.00482221 |
|  |  | ZBTB7B | 0.55 | 2.62E-006 |
| mmu-miR-125b-5p | 1.88 | CD3G | 0.32 | 7.77E-005 |
|  |  | FKBP5 | 0.52 | 0.00392594 |
|  |  | IMPDH2 | 0.62 | 9.40E-005 |
|  |  | RACGAP1 | 0.57 | 2.06E-004 |
| mmu-miR-145 | 1.75 | MCM5 | 0.57 | 0.00164819 |
|  |  | MYC | 0.45 | 1.44E-004 |
|  |  | RPS9 | 0.62 | 1.39E-004 |
|  |  | T | 0.00 | 4.26E-004 |
| mmu-miR-30d | 1.65 | GTF2E2 | 0.62 | 1.06E-005 |
|  |  | GTF2H4 | 0.31 | 0.00312356 |
|  |  | MSH6 | 0.39 | 0.00322064 |
|  |  | PHGDH | 0.42 | 9.01E-006 |
|  |  | SLC35A5 | 0.55 | 8.98E-004 |
|  |  | TBCA | 0.66 | 5.33E-004 |
|  |  | TCF12 | 0.59 | 0.00241971 |
|  |  | TEX10 | 0.52 | 0.00165217 |
|  |  | UBE3C | 0.44 | 2.23E-006 |
| mmu-miR-191 | 1.59 | BCL11B | 0.26 | 5.74E-004 |
|  |  | C1QBP | 0.66 | 3.60E-004 |
|  |  | CDCA8 | 0.35 | 0.00382699 |
|  |  | MAD2L1 | 0.57 | 3.37E-004 |
|  |  | MCM6 | 0.47 | 9.73E-004 |
|  |  | TSSC1 | 0.52 | 1.75E-004 |
|  |  | UBE2C | 0.39 | 9.52E-005 |
| mmu-miR-200b | 0.61 | ARG1 | 9.85 | 0.00142832 |
|  |  | CD72 | 1.93 | 2.27E-004 |
|  |  | CP | 2.14 | 1.23E-004 |
|  |  | LOX | 1.62 | 9.54E-005 |
|  |  | TDO2 | 2.38 | 6.01E-004 |
| mmu-miR-92a | 0.60 | IGF1 | 2.14 | 0.00102133 |
|  |  | KLRG1 | 1.68 | 0.00331721 |
|  |  | TDO2 | 2.38 | 4.52E-004 |
| mmu-miR-27a | 0.54 | 1700080O16RIK | 7.46 | 0.0014603 |
|  |  | AGR2 | 5.66 | 0.00285178 |
| mmu-let-7e | 0.50 | ADAM28 | 1.52 | 0.00229156 |
|  |  | COL6A2 | 1.87 | 7.34E-004 |
|  |  | CXCL16 | 1.62 | 0.00467071 |
| mmu-miR-21 | 0.47 | TGFBI | 1.57 | 4.03E-007 |
| mmu-miR-25 | 0.46 | C3AR1 | 1.87 | 9.72E-004 |
|  |  | KLRG1 | 1.68 | 0.00432717 |
|  |  | SLC26A4 | 10.56 | 0.00420859 |
| mmu-miR-23b | 0.41 | CD163 | 2.22 | 5.07E-005 |
|  |  | CHI3L1 | 2.22 | 0.0023145 |
|  |  | GPX2 | 1.57 | 4.68E-004 |
| mmu-miR-23a | 0.38 | CD163 | 2.22 | 5.07E-005 |
|  |  | CHI3L1 | 2.22 | 0.0023145 |
|  |  | GPX2 | 1.57 | 4.68E-004 |
| mmu-miR-26b | 0.31 | SCIN | 3.36 | 0.0048844 |
| mmu-miR-98 | 0.20 | ADAM28 | 1.52 | 0.00126204 |
|  |  | TDO2 | 2.38 | 5.11E-006 |
| mmu-miR-29c | 0.11 | 1700080O16RIK | 7.46 | 0.00378462 |
|  |  | COL6A2 | 1.87 | 4.48E-009 |
|  |  | CTSK | 1.80 | 6.88E-004 |
|  |  | METRNL | 1.62 | 0.00412566 |

Modulated mature miRNAs with a *p-value* < 0.005, FI: Fold Induction
