# Supplementary material for: MicroRNAs Profiling in Murine Models of Acute and Chronic Asthma: A Relationship with mRNAs Targets
Source: PLoS One. 2011 Jan 28;6(1):e16509. doi: 10.1371/journal.pone.0016509 (PMC3030602; doi:10.1371/journal.pone.0016509)
Supplement: Table S5 — MiRNAs/mRNAs regulatory pathways at ST using MicroCosm Targets. (DOC) [file pone.0016509.s006.doc]

| **Wikipathway** | **Pathway name** | **Total # of genes/pathway** | **miRNA/mRNA interactions*** | **miRna** | | ***p-value* (miRNA)** | **# of modul. mRNA in the pathway**** | **Proportions of mRna** | | ***p-value* (mRNA)** | **combined**  ***p-value*** |
| --- | --- | --- | --- | --- | --- | --- | --- | --- | --- | --- | --- |
|  |  |  |  | **Up** | **Down** |  |  | **Up** | **Down** |  |  |
| WP441 | Matrix Metalloproteinases | 25 | 21 | miR-100 miR-451 miR-20b miR-181b miR-146b miR-223 miR-106a miR-181d miR-122 | miR-672 miR-574-5p miR-483 miR-497 miR-187 | 0.32100 | 6 | 0.67 | 0.33 | 0.00000 | 0.00080 |
| WP190 | Cell Cycle | 84 | 83 | miR-100 miR-451 miR-20b miR-689 miR-181b miR-146b miR-223 miR-106a miR-181d miR-122 miR-712* | miR-805 miR-672 miR-203 miR-574-5p miR-483 miR-1 miR-690 miR-497 miR-187 | 0.15000 | 8 | 1.00 | 0.00 | 0.00001 | 0.00094 |
| WP222 | Cytokines and Inflammatory Response (BioCarta) | 23 | 27 | miR-100 miR-451 miR-181b miR-223 miR-181d miR-122 | miR-805 miR-672 miR-203 miR-574-5p miR-1 miR-497 miR-187 | 0.07000 | 3 | 0.00 | 1.00 | 0.00291 | 0.00138 |
| WP150 | DNA Replication | 41 | 45 | miR-100 miR-451 miR-20b miR-689 miR-181b miR-146b miR-223 miR-106a miR-181d miR-122 miR-712* | miR-805 miR-203 miR-574-5p miR-483 miR-1 miR-690 miR-187 | 0.27900 | 5 | 1.00 | 0.00 | 0.00016 | 0.00154 |
| WP413 | G1 to S Cell Cycle control | 61 | 73 | miR-100 miR-451 miR-20b miR-689 miR-181b miR-146b miR-223 miR-106a miR-181d miR-122 miR-712* | miR-805 miR-672 miR-203 miR-574-5p miR-483 miR-1 miR-690 miR-497 miR-187 | 0.06400 | 4 | 1.00 | 0.00 | 0.00734 | 0.00254 |
| WP449 | Complement and Coagulation Cascades KEGG | 60 | 41 | miR-100 miR-451 miR-20b miR-689 miR-181b miR-146b miR-223 miR-106a miR-181d | miR-805 miR-672 miR-574-5p miR-483 miR-690 miR-497 miR-187 | 0.73100 | 7 | 0.86 | 0.14 | 0.00001 | 0.00498 |
| WP238 | Osteoblast | 8 | 10 | miR-100 miR-451 miR-146b miR-223 miR-106a miR-181d | miR-672 miR-203 miR-497 miR-187 | 0.01000 | 1 | 1.00 | 0.00 | 0.09655 | 0.00516 |
| WP113 | TGF-beta Signaling Pathway | 50 | 41 | miR-100 miR-451 miR-20b miR-689 miR-146b miR-223 miR-106a miR-122 miR-712* | miR-805 miR-672 miR-574-5p miR-483 miR-1 miR-690 miR-497 miR-187 | 0.17400 | 4 | 0.50 | 0.50 | 0.00361 | 0.00518 |
| WP426 | Urea cycle and Metabolism of Amino groups | 20 | 22 | miR-451 miR-689 miR-181b miR-146b miR-223 miR-106a miR-181d miR-122 | miR-805 miR-203 miR-574-5p miR-690 miR-497 miR-187 | 0.36800 | 3 | 1.00 | 0.00 | 0.00193 | 0.01125 |
| WP385 | Myometrial Relaxation and Contraction Pathways | 159 | 150 | miR-100 miR-451 miR-20b miR-689 miR-181b miR-146b miR-223 miR-106a miR-181d miR-122 miR-712* | miR-805 miR-672 miR-203 miR-574-5p miR-483 miR-1 miR-690 miR-497 miR-187 | 0.03200 | 4 | 0.25 | 0.75 | 0.14190 | 0.01934 |
| WP460 | Blood Clotting Cascade | 18 | 16 | miR-100 miR-689 miR-181b miR-146b miR-223 miR-181d | miR-672 miR-574-5p miR-483 miR-1 miR-187 | 0.52800 | 3 | 0.67 | 0.33 | 0.00141 | 0.01956 |
| WP373 | IL-3 Signaling Pathway | 95 | 71 | miR-100 miR-451 miR-20b miR-689 miR-181b miR-146b miR-223 miR-106a miR-181d miR-122 miR-712* | miR-805 miR-672 miR-203 miR-574-5p miR-483 miR-1 miR-690 miR-497 miR-187 | 0.43600 | 5 | 0.40 | 0.60 | 0.00708 | 0.03225 |
| WP252 | Androgen Receptor Signaling Pathway | 105 | 92 | miR-100 miR-451 miR-20b miR-689 miR-181b miR-146b miR-223 miR-106a miR-181d miR-122 miR-712* | miR-805 miR-672 miR-203 miR-574-5p miR-483 miR-1 miR-690 miR-497 miR-187 | 0.43500 | 5 | 0.60 | 0.40 | 0.01066 | 0.04061 |
| WP57 | Signal Transduction of S1P Receptor | 22 | 26 | miR-100 miR-451 miR-20b miR-689 miR-181b miR-146b miR-106a miR-181d miR-122 miR-712* | miR-805 miR-483 miR-690 miR-497 miR-187 | 0.03900 | 1 | 1.00 | 0.00 | 0.24370 | 0.04117 |
| WP274 | B Cell Receptor Signaling Pathway | 149 | 123 | miR-100 miR-451 miR-20b miR-689 miR-181b miR-146b miR-223 miR-106a miR-181d miR-122 miR-712* | miR-805 miR-672 miR-203 miR-574-5p miR-483 miR-1 miR-690 miR-497 miR-187 | 0.25600 | 5 | 0.80 | 0.20 | 0.04071 | 0.04496 |
| WP85 | Focal Adhesion | 183 | 144 | miR-100 miR-451 miR-20b miR-689 miR-181b miR-146b miR-223 miR-106a miR-181d miR-122 miR-712* | miR-805 miR-672 miR-203 miR-574-5p miR-483 miR-1 miR-690 miR-497 miR-187 | 0.67000 | 8 | 0.88 | 0.13 | 0.00231 | 0.04532 |

Combined *p-value* < 0.05. * Number of interactions between modulated miRNA and genes present in the pathway. ** Number of modulated mRNA associated with genes of the pathway.
