# Supplementary material for: MicroRNAs Profiling in Murine Models of Acute and Chronic Asthma: A Relationship with mRNAs Targets
Source: PLoS One. 2011 Jan 28;6(1):e16509. doi: 10.1371/journal.pone.0016509 (PMC3030602; doi:10.1371/journal.pone.0016509)
Supplement: Table S6 — MiRNAs/mRNAs regulatory pathways at IT using MicroCosm Targets. (DOC) [file pone.0016509.s007.doc]

| **Wikipathway** | **Pathway name** | **Total # of genes/pathway** | **miRNA/mRNA interactions*** | **miRna** | | ***p-value* (miRNA)** | **# of modul. mRNA in the pathway**** | **Proportions of mRna** | | ***p-value***  **(mRNA)** | **combined**  ***p-value*** |
| --- | --- | --- | --- | --- | --- | --- | --- | --- | --- | --- | --- |
|  |  |  |  | **Up** | **Down** |  |  | **Up** | **Down** |  |  |
| WP246 | TNF-alpha/NF-kB Signaling Pathway | 174 | 251 | miR-375 miR-146b miR-423-5p miR-150 miR-455 miR-466f-3p miR-466g miR-467a* miR-467b* miR-155 | miR-126-5p miR-690 miR-19b miR-223 miR-101a miR-29b miR-10b miR-200a miR-10a miR-29c miR-218 miR-152 miR-199a-3p miR-322 miR-429 | 0.00000 | 10 | 0.90 | 0.10 | 0.00898 | 0.00000 |
| WP310 | mRNA processing | 441 | 548 | miR-375 miR-146b miR-423-5p miR-150 miR-455 miR-466f-3p miR-466g miR-467a* miR-467b* miR-155 | miR-126-5p miR-450a-5p miR-690 miR-19b miR-223 miR-101a miR-29b miR-10b miR-200a miR-10a miR-29c miR-218 miR-152 miR-199a-3p miR-322 miR-429 | 0.00000 | 17 | 1.00 | 0.00 | 0.03596 | 0.00000 |
| WP85 | Focal Adhesion | 183 | 223 | miR-375 miR-146b miR-423-5p miR-150 miR-455 miR-466f-3p miR-466g miR-467a* miR-467b* miR-155 | miR-126-5p miR-450a-5p miR-690 miR-19b miR-223 miR-101a miR-29b miR-10b miR-200a miR-10a miR-29c miR-218 miR-152 miR-199a-3p miR-322 miR-429 | 0.03600 | 15 | 1.00 | 0.00 | 0.00003 | 0.00002 |
| WP258 | TGF-beta Receptor Signaling Pathway | 147 | 169 | miR-375 miR-146b miR-423-5p miR-150 miR-455 miR-466f-3p miR-466g miR-467a* miR-467b* miR-155 | miR-126-5p miR-450a-5p miR-690 miR-19b miR-223 miR-101a miR-29b miR-10b miR-200a miR-10a miR-29c miR-218 miR-152 miR-199a-3p miR-322 miR-429 | 0.05500 | 13 | 1.00 | 0.00 | 0.00005 | 0.00005 |
| WP252 | Androgen Receptor Signaling Pathway | 105 | 124 | miR-375 miR-146b miR-423-5p miR-150 miR-455 miR-466f-3p miR-466g miR-467a* miR-467b* miR-155 | miR-126-5p miR-450a-5p miR-690 miR-19b miR-223 miR-101a miR-29b miR-10b miR-200a miR-10a miR-29c miR-218 miR-152 miR-199a-3p miR-322 miR-429 | 0.27900 | 13 | 1.00 | 0.00 | 0.00000 | 0.00009 |
| WP385 | Myometrial Relaxation and Contraction Pathways | 159 | 184 | miR-375 miR-146b miR-423-5p miR-150 miR-455 miR-466f-3p miR-466g miR-467a* miR-467b* miR-155 | miR-126-5p miR-450a-5p miR-690 miR-19b miR-223 miR-101a miR-29b miR-10b miR-200a miR-10a miR-29c miR-218 miR-152 miR-199a-3p miR-322 miR-429 | 0.09300 | 13 | 0.92 | 0.08 | 0.00012 | 0.00020 |
| WP450 | IL-2 Signaling Pathway | 73 | 76 | miR-375 miR-146b miR-150 miR-455 miR-466f-3p miR-466g miR-467a* miR-467b* miR-155 | miR-690 miR-19b miR-223 miR-101a miR-29b miR-10b miR-200a miR-10a miR-29c miR-218 miR-199a-3p miR-322 miR-429 | 0.05800 | 8 | 1.00 | 0.00 | 0.00034 | 0.00022 |
| WP447 | Adipogenesis Mouse | 131 | 142 | miR-375 miR-146b miR-423-5p miR-150 miR-455 miR-466f-3p miR-466g miR-467a* miR-467b* miR-155 | miR-126-5p miR-450a-5p miR-690 miR-19b miR-223 miR-101a miR-29b miR-10b miR-200a miR-10a miR-29c miR-218 miR-152 miR-199a-3p miR-322 miR-429 | 0.22100 | 13 | 1.00 | 0.00 | 0.00002 | 0.00024 |
| WP373 | IL-3 Signaling Pathway | 95 | 106 | miR-375 miR-146b miR-423-5p miR-150 miR-455 miR-466f-3p miR-466g miR-467a* miR-467b* miR-155 | miR-450a-5p miR-690 miR-19b miR-223 miR-101a miR-29b miR-10b miR-200a miR-10a miR-29c miR-152 miR-199a-3p miR-322 miR-429 | 0.06300 | 9 | 1.00 | 0.00 | 0.00044 | 0.00030 |
| WP572 | EGFR1 Signaling Pathway | 171 | 173 | miR-375 miR-146b miR-423-5p miR-150 miR-455 miR-466f-3p miR-466g miR-467a* miR-467b* miR-155 | miR-126-5p miR-450a-5p miR-690 miR-19b miR-223 miR-101a miR-29b miR-10b miR-200a miR-10a miR-29c miR-218 miR-152 miR-199a-3p miR-322 miR-429 | 0.14700 | 12 | 1.00 | 0.00 | 0.00083 | 0.00151 |
| WP539 | Wnt Signaling Pathway NetPath | 107 | 103 | miR-375 miR-146b miR-423-5p miR-150 miR-455 miR-466f-3p miR-466g miR-155 | miR-126-5p miR-450a-5p miR-690 miR-19b miR-223 miR-101a miR-29b miR-10b miR-200a miR-10a miR-29c miR-218 miR-152 miR-199a-3p miR-322 miR-429 | 0.07400 | 7 | 1.00 | 0.00 | 0.01406 | 0.00501 |
| WP493 | MAPK Signaling Pathway | 133 | 142 | miR-375 miR-146b miR-423-5p miR-150 miR-455 miR-466f-3p miR-466g miR-467a* miR-467b* miR-155 | miR-126-5p miR-450a-5p miR-690 miR-19b miR-223 miR-101a miR-29b miR-10b miR-200a miR-10a miR-29c miR-218 miR-152 miR-199a-3p miR-322 miR-429 | 0.27300 | 10 | 1.00 | 0.00 | 0.00132 | 0.00535 |
| WP441 | Matrix Metalloproteinases | 25 | 25 | miR-375 miR-146b miR-423-5p miR-455 miR-466f-3p miR-155 | miR-450a-5p miR-19b miR-223 miR-29b miR-200a miR-29c miR-218 miR-322 miR-429 | 0.45500 | 5 | 1.00 | 0.00 | 0.00027 | 0.00574 |
| WP113 | TGF-beta Signaling Pathway | 50 | 48 | miR-375 miR-146b miR-423-5p miR-150 miR-455 miR-466f-3p miR-467a* miR-467b* miR-155 | miR-126-5p miR-690 miR-19b miR-223 miR-101a miR-29b miR-10b miR-10a miR-29c miR-218 miR-152 miR-199a-3p miR-322 miR-429 | 0.37300 | 6 | 1.00 | 0.00 | 0.00115 | 0.00856 |
| WP151 | IL-5 Signaling Pathway | 65 | 57 | miR-375 miR-146b miR-423-5p miR-150 miR-455 miR-466g miR-155 | miR-690 miR-19b miR-223 miR-101a miR-29b miR-10b miR-200a miR-10a miR-29c miR-199a-3p miR-322 miR-429 | 0.76200 | 9 | 1.00 | 0.00 | 0.00002 | 0.00859 |
| WP523 | Regulation of Actin Cytoskeleton | 146 | 144 | miR-375 miR-146b miR-423-5p miR-150 miR-455 miR-466f-3p miR-466g miR-467a* miR-467b* miR-155 | miR-126-5p miR-450a-5p miR-690 miR-19b miR-223 miR-101a miR-29b miR-10b miR-200a miR-10a miR-29c miR-218 miR-152 miR-199a-3p miR-322 miR-429 | 0.51800 | 11 | 1.00 | 0.00 | 0.00076 | 0.01353 |
| WP168 | Apoptosis Mechanisms | 79 | 105 | miR-375 miR-146b miR-150 miR-455 miR-466f-3p miR-466g miR-467a* miR-467b* miR-155 | miR-126-5p miR-450a-5p miR-690 miR-19b miR-223 miR-101a miR-29b miR-10b miR-200a miR-10a miR-29c miR-218 miR-152 miR-199a-3p miR-322 miR-429 | 0.03100 | 4 | 1.00 | 0.00 | 0.11928 | 0.01566 |
| WP553 | Calcium Regulation in the Cardiac Cell | 148 | 144 | miR-375 miR-146b miR-423-5p miR-150 miR-455 miR-466f-3p miR-466g miR-467a* miR-467b* miR-155 | miR-126-5p miR-450a-5p miR-690 miR-19b miR-223 miR-101a miR-29b miR-10b miR-200a miR-10a miR-29c miR-218 miR-152 miR-199a-3p miR-322 miR-429 | 0.18700 | 8 | 1.00 | 0.00 | 0.02551 | 0.02230 |
| WP234 | Peptide GPCRs | 70 | 74 | miR-375 miR-146b miR-423-5p miR-150 miR-455 miR-466f-3p miR-466g miR-467a* miR-467b* miR-155 | miR-126-5p miR-450a-5p miR-690 miR-19b miR-223 miR-101a miR-29b miR-10b miR-200a miR-10a miR-29c miR-218 miR-152 miR-199a-3p miR-322 miR-429 | 0.07200 | 4 | 1.00 | 0.00 | 0.08549 | 0.02269 |
| WP339 | Mouse ESC Pluripotency Pathways | 109 | 119 | miR-375 miR-146b miR-423-5p miR-150 miR-455 miR-466f-3p miR-466g miR-467a* miR-467b* miR-155 | miR-126-5p miR-450a-5p miR-690 miR-19b miR-223 miR-101a miR-29b miR-10b miR-200a miR-10a miR-29c miR-218 miR-152 miR-199a-3p miR-322 miR-429 | 0.05000 | 5 | 1.00 | 0.00 | 0.11884 | 0.02286 |
| WP544 | Circadian Exercise | 49 | 51 | miR-375 miR-146b miR-423-5p miR-150 miR-466f-3p miR-466g miR-467a* miR-467b* miR-155 | miR-126-5p miR-450a-5p miR-690 miR-19b miR-223 miR-29b miR-10b miR-200a miR-10a miR-29c miR-218 miR-152 miR-199a-3p miR-322 miR-429 | 0.17800 | 4 | 1.00 | 0.00 | 0.02893 | 0.02308 |
| WP6 | Integrin-mediated Cell Adhesion | 97 | 98 | miR-375 miR-146b miR-423-5p miR-150 miR-455 miR-466f-3p miR-466g miR-467a* miR-467b* miR-155 | miR-126-5p miR-450a-5p miR-690 miR-19b miR-223 miR-101a miR-29b miR-10b miR-200a miR-10a miR-29c miR-218 miR-152 miR-199a-3p miR-322 miR-429 | 0.72500 | 9 | 1.00 | 0.00 | 0.00051 | 0.02871 |
| WP297 | IL-7 Signaling Pathway | 42 | 47 | miR-375 miR-146b miR-150 miR-455 miR-466f-3p miR-466g miR-467a* miR-467b* miR-155 | miR-690 miR-19b miR-223 miR-101a miR-29b miR-200a miR-29c miR-218 miR-152 miR-199a-3p miR-322 miR-429 | 0.11100 | 3 | 1.00 | 0.00 | 0.07781 | 0.03091 |
| WP274 | B Cell Receptor Signaling Pathway | 149 | 157 | miR-375 miR-146b miR-423-5p miR-150 miR-455 miR-466f-3p miR-466g miR-467a* miR-467b* miR-155 | miR-126-5p miR-450a-5p miR-690 miR-19b miR-223 miR-101a miR-29b miR-10b miR-200a miR-10a miR-29c miR-218 miR-152 miR-199a-3p miR-322 miR-429 | 0.28600 | 8 | 1.00 | 0.00 | 0.02642 | 0.03847 |
| WP193 | Signaling of Hepatocyte Growth Factor Receptor | 33 | 31 | miR-375 miR-155 | miR-19b miR-223 miR-101a miR-29b miR-10b miR-200a miR-10a miR-29c miR-152 miR-199a-3p miR-322 miR-429 | 0.26500 | 3 | 1.00 | 0.00 | 0.04315 | 0.04876 |

Combined *p-value* < 0.05. * Number of interactions between modulated miRNA and genes present in the pathway. ** Number of modulated mRNA associated with genes of the pathway.
