# Supplementary material for: MicroRNAs Profiling in Murine Models of Acute and Chronic Asthma: A Relationship with mRNAs Targets
Source: PLoS One. 2011 Jan 28;6(1):e16509. doi: 10.1371/journal.pone.0016509 (PMC3030602; doi:10.1371/journal.pone.0016509)
Supplement: Table S7 — MiRNAs/mRNAs regulatory pathways at LT using MicroCosm Targets. (DOC) [file pone.0016509.s008.doc]

| **Wikipathway** | **Pathway name** | **Total # of genes/pathway** | **miRNA/mRNA interactions*** | **miRna** | | ***p-value* (miRNA)** | **# of modul. mRNA in the pathway**** | **Proportions of mRna** | | ***p-value***  **(mRNA)** | **combined**  ***p-value*** |
| --- | --- | --- | --- | --- | --- | --- | --- | --- | --- | --- | --- |
|  |  |  |  | **Up** | **Down** |  |  | **Up** | **Down** |  |  |
| WP168 | Apoptosis Mechanisms | 79 | 283 | miR-191 miR-30d miR-145 miR-125b-5p miR-99b miR-146a miR-146b miR-720 miR-143 miR-151-3p miR-674 miR-34c* miR-320 miR-214 miR-672 miR-328 miR-206 miR-466h miR-346 miR-207 miR-15a* miR-297a miR-877* miR-455 miR-197 miR-466a-3p miR-468 miR-297a* miR-466f-5p miR-466b-3-3p miR-466d-3p miR-762 let-7d* miR-713 miR-574-5p miR-667 miR-574-3p miR-466g miR-466c-5p miR-467e* miR-709 miR-685 miR-466f-3p miR-744 miR-467a* miR-671-5p miR-691 miR-467b* miR-568 miR-669c miR-483 miR-188-5p miR-705 | miR-29c miR-15a miR-98 miR-26b miR-23a miR-23b miR-30b miR-25 miR-21 let-7e miR-27a miR-30c miR-92a miR-200b | 0.00000 | 3 | 0 | 1 | 0.13442 | 0.00000 |
| WP246 | TNF-alpha/NF-kB Signaling Pathway | 174 | 573 | miR-191 miR-30d miR-145 miR-125b-5p miR-99b miR-146a miR-146b miR-720 miR-143 miR-151-3p miR-674 miR-423-5p miR-34c* miR-320 miR-214 miR-672 miR-328 miR-206 miR-466h miR-346 miR-207 miR-15a* miR-297a miR-877* miR-455 miR-197 miR-466a-3p miR-468 miR-297a* miR-466f-5p miR-466b-3-3p miR-466d-3p miR-762 let-7d* miR-713 miR-574-5p miR-667 miR-574-3p miR-466g miR-466c-5p miR-467e* miR-709 miR-685 miR-466f-3p miR-744 miR-485* miR-467a* miR-671-5p miR-691 miR-467b* miR-568 miR-669c miR-483 miR-188-5p miR-705 | miR-29c miR-15a miR-98 miR-26b miR-23a miR-23b miR-30b miR-25 miR-21 let-7e miR-27a miR-30c miR-92a miR-200b | 0.00000 | 7 | 0.00 | 1.00 | 0.02274 | 0.00000 |
| WP93 | IL-4 Signaling Pathway | 58 | 187 | miR-191 miR-30d miR-145 miR-99b miR-146a miR-146b miR-720 miR-674 miR-423-5p miR-320 miR-214 miR-672 miR-328 miR-206 miR-466h miR-207 miR-15a* miR-297a miR-877* miR-455 miR-197 miR-466a-3p miR-297a* miR-466f-5p miR-466b-3-3p miR-466d-3p miR-762 let-7d* miR-713 miR-574-5p miR-574-3p miR-466g miR-466c-5p miR-467e* miR-709 miR-685 miR-466f-3p miR-744 miR-485* miR-467a* miR-671-5p miR-691 miR-467b* miR-568 miR-669c miR-483 miR-188-5p miR-705 | miR-29c miR-15a miR-98 miR-26b miR-23a miR-23b miR-30b miR-25 miR-21 let-7e miR-27a miR-30c miR-92a miR-200b | 0.00000 | 2 | 0.00 | 1.00 | 0.23921 | 0.00000 |
| WP480 | T Cell Receptor Signaling Pathway | 125 | 395 | miR-191 miR-30d miR-145 miR-125b-5p miR-99b miR-146a miR-146b miR-720 miR-143 miR-151-3p miR-674 miR-423-5p miR-34c* miR-320 miR-214 miR-672 miR-328 miR-206 miR-466h miR-346 miR-207 miR-15a* miR-297a miR-877* miR-455 miR-197 miR-466a-3p miR-468 miR-297a* miR-466f-5p miR-466b-3-3p miR-466d-3p miR-762 let-7d* miR-713 miR-574-5p miR-667 miR-574-3p miR-466g miR-466c-5p miR-467e* miR-709 miR-685 miR-466f-3p miR-744 miR-485* miR-467a* miR-671-5p miR-691 miR-467b* miR-568 miR-669c miR-483 miR-188-5p miR-705 | miR-29c miR-15a miR-98 miR-26b miR-23a miR-23b miR-30b miR-25 miR-21 let-7e miR-27a miR-30c miR-92a miR-200b | 0.02200 | 11 | 0.00 | 1.00 | 0.00001 | 0.00000 |
| WP190 | Cell Cycle | 84 | 280 | miR-191 miR-30d miR-145 miR-125b-5p miR-99b miR-146a miR-146b miR-720 miR-143 miR-151-3p miR-674 miR-423-5p miR-34c* miR-320 miR-214 miR-672 miR-328 miR-206 miR-466h miR-346 miR-15a* miR-297a miR-877* miR-455 miR-197 miR-466a-3p miR-468 miR-297a* miR-466f-5p miR-466b-3-3p miR-466d-3p miR-762 let-7d* miR-713 miR-574-5p miR-667 miR-574-3p miR-466g miR-466c-5p miR-467e* miR-709 miR-685 miR-466f-3p miR-744 miR-485* miR-467a* miR-671-5p miR-691 miR-467b* miR-568 miR-669c miR-483 miR-188-5p miR-705 | miR-29c miR-15a miR-98 miR-26b miR-23a miR-23b miR-30b miR-25 miR-21 let-7e miR-27a miR-30c miR-92a miR-200b | 0.05400 | 9 | 0.00 | 1.00 | 0.00001 | 0.00001 |
| WP252 | Androgen Receptor Signaling Pathway | 105 | 356 | miR-191 miR-30d miR-145 miR-125b-5p miR-99b miR-146a miR-146b miR-720 miR-143 miR-151-3p miR-674 miR-423-5p miR-34c* miR-320 miR-214 miR-672 miR-328 miR-206 miR-466h miR-346 miR-207 miR-15a* miR-297a miR-877* miR-455 miR-197 miR-466a-3p miR-297a* miR-466f-5p miR-466b-3-3p miR-466d-3p miR-762 let-7d* miR-713 miR-574-5p miR-667 miR-574-3p miR-466g miR-466c-5p miR-467e* miR-709 miR-685 miR-466f-3p miR-744 miR-485* miR-467a* miR-671-5p miR-691 miR-467b* miR-568 miR-669c miR-483 miR-188-5p miR-705 | miR-29c miR-15a miR-98 miR-26b miR-23a miR-23b miR-30b miR-25 miR-21 let-7e miR-27a miR-30c miR-92a miR-200b | 0.00400 | 7 | 0.00 | 1.00 | 0.00155 | 0.00004 |
| WP310 | mRNA processing | 441 | 1297 | miR-191 miR-30d miR-145 miR-125b-5p miR-99b miR-146a miR-146b miR-720 miR-143 miR-151-3p miR-674 miR-423-5p miR-34c* miR-320 miR-214 miR-672 miR-328 miR-206 miR-466h miR-346 miR-207 miR-15a* miR-297a miR-877* miR-455 miR-197 miR-466a-3p miR-468 miR-297a* miR-466f-5p miR-466b-3-3p miR-466d-3p miR-762 let-7d* miR-713 miR-574-5p miR-667 miR-574-3p miR-466g miR-466c-5p miR-467e* miR-709 miR-685 miR-466f-3p miR-744 miR-485* miR-467a* miR-671-5p miR-691 miR-467b* miR-568 miR-669c miR-483 miR-188-5p miR-705 | miR-29c miR-15a miR-98 miR-26b miR-23a miR-23b miR-30b miR-25 miR-21 let-7e miR-27a miR-30c miR-92a miR-200b | 0.04500 | 18 | 0.00 | 1.00 | 0.00031 | 0.00015 |
| WP150 | DNA Replication | 41 | 157 | miR-191 miR-30d miR-145 miR-125b-5p miR-99b miR-146a miR-146b miR-720 miR-143 miR-151-3p miR-674 miR-423-5p miR-34c* miR-320 miR-214 miR-328 miR-206 miR-466h miR-346 miR-207 miR-15a* miR-297a miR-877* miR-455 miR-197 miR-466a-3p miR-468 miR-297a* miR-466f-5p miR-466b-3-3p miR-466d-3p miR-762 let-7d* miR-713 miR-574-5p miR-667 miR-574-3p miR-466g miR-466c-5p miR-467e* miR-709 miR-685 miR-466f-3p miR-744 miR-485* miR-467a* miR-671-5p miR-467b* miR-568 miR-669c miR-483 miR-188-5p miR-705 | miR-29c miR-98 miR-23a miR-23b miR-30b miR-25 miR-21 let-7e miR-27a miR-30c miR-92a miR-200b | 0.11700 | 6 | 0.00 | 1.00 | 0.00005 | 0.00016 |
| WP493 | MAPK Signaling Pathway | 133 | 401 | miR-191 miR-30d miR-145 miR-125b-5p miR-99b miR-146a miR-146b miR-720 miR-143 miR-151-3p miR-674 miR-423-5p miR-34c* miR-320 miR-214 miR-672 miR-328 miR-206 miR-466h miR-346 miR-207 miR-15a* miR-297a miR-877* miR-455 miR-197 miR-466a-3p miR-468 miR-297a* miR-466f-5p miR-466b-3-3p miR-466d-3p miR-762 let-7d* miR-713 miR-667 miR-574-3p miR-466g miR-466c-5p miR-467e* miR-709 miR-685 miR-466f-3p miR-744 miR-485* miR-467a* miR-671-5p miR-691 miR-467b* miR-568 miR-669c miR-483 miR-188-5p miR-705 | miR-29c miR-15a miR-98 miR-26b miR-23a miR-23b miR-30b miR-25 miR-21 let-7e miR-27a miR-30c miR-92a miR-200b | 0.01000 | 5 | 0.00 | 1.00 | 0.06385 | 0.00324 |
| WP441 | Matrix Metalloproteinases | 25 | 71 | miR-191 miR-145 miR-125b-5p miR-99b miR-146a miR-146b miR-143 miR-151-3p miR-674 miR-423-5p miR-320 miR-214 miR-672 miR-328 miR-466h miR-346 miR-207 miR-15a* miR-297a miR-455 miR-197 miR-468 miR-466f-5p miR-466d-3p miR-762 let-7d* miR-574-5p miR-667 miR-574-3p miR-466c-5p miR-709 miR-685 miR-466f-3p miR-744 miR-671-5p miR-691 miR-568 miR-669c miR-483 | miR-29c miR-98 miR-26b miR-25 let-7e miR-27a miR-92a | 0.21300 | 3 | 0.67 | 0.33 | 0.00729 | 0.01101 |
| WP373 | IL-3 Signaling Pathway | 95 | 276 | miR-191 miR-30d miR-145 miR-125b-5p miR-99b miR-146a miR-146b miR-720 miR-143 miR-151-3p miR-674 miR-423-5p miR-320 miR-214 miR-672 miR-328 miR-206 miR-466h miR-346 miR-207 miR-15a* miR-297a miR-877* miR-455 miR-197 miR-466a-3p miR-468 miR-297a* miR-466f-5p miR-466b-3-3p miR-466d-3p miR-762 let-7d* miR-713 miR-574-5p miR-667 miR-574-3p miR-466g miR-466c-5p miR-467e* miR-709 miR-685 miR-466f-3p miR-744 miR-485* miR-467a* miR-671-5p miR-691 miR-467b* miR-568 miR-669c miR-483 miR-188-5p miR-705 | miR-29c miR-15a miR-98 miR-26b miR-23a miR-23b miR-30b miR-25 miR-21 let-7e miR-27a miR-30c miR-92a miR-200b | 0.01100 | 3 | 0.00 | 1.00 | 0.19683 | 0.01312 |
| WP571 | FAS Pathway and Stress induction of HSP regulation | 38 | 134 | miR-191 miR-30d miR-125b-5p miR-99b miR-146a miR-146b miR-720 miR-143 miR-674 miR-423-5p miR-320 miR-214 miR-672 miR-328 miR-206 miR-207 miR-15a* miR-297a miR-455 miR-466a-3p miR-468 miR-297a* miR-466f-5p miR-466b-3-3p miR-466d-3p miR-762 miR-574-5p miR-574-3p miR-466g miR-466c-5p miR-467e* miR-709 miR-685 miR-466f-3p miR-485* miR-467a* miR-691 miR-467b* miR-568 miR-669c miR-483 miR-188-5p miR-705 | miR-29c miR-15a miR-98 miR-23a miR-23b miR-30b miR-25 miR-21 let-7e miR-27a miR-30c miR-92a miR-200b | 0.00200 | 1 | 0.00 | 1.00 | 0.46019 | 0.01761 |
| WP297 | IL-7 Signaling Pathway | 42 | 121 | miR-145 miR-125b-5p miR-99b miR-146a miR-146b miR-720 miR-143 miR-151-3p miR-674 miR-34c* miR-320 miR-214 miR-672 miR-328 miR-206 miR-466h miR-346 miR-207 miR-15a* miR-297a miR-877* miR-455 miR-197 miR-466a-3p miR-466f-5p miR-466b-3-3p miR-466d-3p miR-762 miR-713 miR-574-5p miR-667 miR-574-3p miR-466g miR-467e* miR-709 miR-685 miR-466f-3p miR-744 miR-467a* miR-671-5p miR-691 miR-467b* miR-568 miR-188-5p miR-705 | miR-29c miR-15a miR-26b miR-23a miR-23b miR-30b miR-25 miR-21 let-7e miR-27a miR-30c miR-92a miR-200b | 0.03200 | 2 | 0.00 | 1.00 | 0.14624 | 0.01998 |
| WP450 | IL-2 Signaling Pathway | 73 | 187 | miR-191 miR-30d miR-145 miR-125b-5p miR-99b miR-146a miR-146b miR-720 miR-143 miR-674 miR-34c* miR-320 miR-214 miR-672 miR-328 miR-206 miR-466h miR-207 miR-15a* miR-297a miR-877* miR-455 miR-197 miR-466a-3p miR-468 miR-297a* miR-466f-5p miR-466b-3-3p miR-466d-3p miR-762 let-7d* miR-713 miR-667 miR-574-3p miR-466g miR-466c-5p miR-467e* miR-709 miR-685 miR-466f-3p miR-744 miR-467a* miR-671-5p miR-691 miR-467b* miR-568 miR-669c miR-483 miR-188-5p miR-705 | miR-29c miR-15a miR-98 miR-26b miR-23a miR-23b miR-30b miR-25 miR-21 let-7e miR-30c miR-92a miR-200b | 0.05100 | 3 | 0.00 | 1.00 | 0.11312 | 0.02211 |
| WP456 | GPCRs -- Class B Secretin-like | 13 | 48 | miR-191 miR-125b-5p miR-146a miR-151-3p miR-423-5p miR-320 miR-672 miR-328 miR-206 miR-466h miR-346 miR-207 miR-877* miR-455 miR-468 miR-466b-3-3p miR-466d-3p miR-762 let-7d* miR-713 miR-574-5p miR-574-3p miR-709 miR-685 miR-466f-3p miR-485* miR-671-5p miR-568 miR-669c miR-483 miR-188-5p miR-705 | miR-15a let-7e | 0.03600 | 1 | 1.00 | 0.00 | 0.19006 | 0.02919 |
| WP567 | Eukaryotic Transcription Initiation | 40 | 165 | miR-191 miR-30d miR-145 miR-125b-5p miR-99b miR-146a miR-146b miR-720 miR-143 miR-151-3p miR-674 miR-423-5p miR-34c* miR-320 miR-214 miR-672 miR-328 miR-466h miR-346 miR-15a* miR-455 miR-466a-3p miR-468 miR-297a* miR-466f-5p miR-466b-3-3p miR-466d-3p miR-762 let-7d* miR-713 miR-574-5p miR-667 miR-574-3p miR-466g miR-466c-5p miR-467e* miR-709 miR-685 miR-466f-3p miR-744 miR-467a* miR-691 miR-467b* miR-568 miR-669c miR-483 miR-188-5p | miR-29c miR-15a miR-98 miR-26b miR-23a miR-23b miR-30b miR-25 miR-21 let-7e miR-27a miR-30c miR-92a miR-200b | 0.09500 | 2 | 0.00 | 1.00 | 0.13520 | 0.04400 |
| WP274 | B Cell Receptor Signaling Pathway | 149 | 417 | miR-191 miR-30d miR-145 miR-125b-5p miR-99b miR-146a miR-146b miR-720 miR-143 miR-151-3p miR-674 miR-423-5p miR-34c* miR-320 miR-214 miR-672 miR-328 miR-206 miR-466h miR-346 miR-207 miR-15a* miR-297a miR-877* miR-455 miR-197 miR-466a-3p miR-468 miR-297a* miR-466f-5p miR-466b-3-3p miR-466d-3p miR-762 let-7d* miR-713 miR-574-5p miR-667 miR-574-3p miR-466g miR-466c-5p miR-467e* miR-709 miR-685 miR-466f-3p miR-744 miR-485* miR-467a* miR-671-5p miR-691 miR-467b* miR-568 miR-669c miR-483 miR-188-5p miR-705 | miR-29c miR-15a miR-98 miR-26b miR-23a miR-23b miR-30b miR-25 miR-21 let-7e miR-27a miR-30c miR-92a miR-200b | 0.14400 | 5 | 0.00 | 1.00 | 0.09298 | 0.04584 |
| WP116 | Hedgehog Signaling Pathway | 21 | 64 | miR-30d miR-145 miR-125b-5p miR-720 miR-143 miR-151-3p miR-674 miR-423-5p miR-34c* miR-214 miR-672 miR-466h miR-346 miR-297a miR-455 miR-197 miR-466a-3p miR-468 miR-297a* miR-466f-5p miR-466b-3-3p miR-466d-3p let-7d* miR-574-5p miR-667 miR-574-3p miR-466g miR-466c-5p miR-467e* miR-466f-3p miR-485* miR-467a* miR-467b* miR-568 miR-669c miR-483 miR-705 | miR-29c miR-15a miR-26b miR-23a miR-23b miR-25 miR-21 let-7e miR-30c miR-92a miR-200b | 0.03500 | 1 | 0.00 | 1.00 | 0.28864 | 0.04694 |
| WP519 | Proteasome Degradation | 78 | 176 | miR-191 miR-30d miR-145 miR-125b-5p miR-99b miR-146a miR-146b miR-720 miR-143 miR-151-3p miR-674 miR-423-5p miR-34c* miR-320 miR-214 miR-672 miR-206 miR-466h miR-346 miR-207 miR-15a* miR-297a miR-877* miR-455 miR-197 miR-466a-3p miR-468 miR-297a* miR-466f-5p miR-466b-3-3p miR-466d-3p miR-762 let-7d* miR-713 miR-574-5p miR-667 miR-574-3p miR-466g miR-466c-5p miR-709 miR-685 miR-466f-3p miR-485* miR-671-5p miR-691 miR-568 miR-669c miR-483 miR-188-5p miR-705 | miR-29c miR-15a miR-98 miR-26b miR-23a miR-23b miR-30b miR-25 miR-21 let-7e miR-27a miR-30c miR-92a miR-200b | 0.10700 | 3 | 0.00 | 1.00 | 0.13078 | 0.04721 |

Combined *p-value* < 0.05. * Number of interactions between modulated miRNA and genes present in the pathway. ** Number of modulated mRNA associated with genes of the pathway.
