# Supplementary material for: MicroRNAs Profiling in Murine Models of Acute and Chronic Asthma: A Relationship with mRNAs Targets
Source: PLoS One. 2011 Jan 28;6(1):e16509. doi: 10.1371/journal.pone.0016509 (PMC3030602; doi:10.1371/journal.pone.0016509)
Supplement: Table S8 — MiRNAs/mRNAs regulatory pathways in mice model of asthma at ST using TargetScan. (DOC) [file pone.0016509.s009.doc]

| **Wikipathway** | **Pathway name** | **Total # of genes/pathway** | **miRNA/mRNA interactions*** | **miRna** | | ***p-value* (miRNA)** | **# of modul. mRNA in the pathway**** | **Proportions of mRna** | | ***p-value* (mRNA)** | **combined**  ***p-value*** |
| --- | --- | --- | --- | --- | --- | --- | --- | --- | --- | --- | --- |
|  |  |  |  | **Up** | **Down** |  |  | **Up** | **Down** |  |  |
| WP190 | Cell cycle | 84 | 21 | miR-223 | miR-203 miR-1 miR-497 | 0.22600 | 8 | 1.00 | 0.00 | 0.00001 | 0.00021 |
| WP441 | Matrix Metalloproteinases | 25 | 2 |  | miR-203 miR-1 | 0.66800 | 6 | 0.67 | 0.33 | 0.00000 | 0.00085 |
| WP449 | Complement and Coagulation Cascades KEGG | 60 | 2 | miR-223 | miR-497 | 0.46500 | 7 | 0.86 | 0.14 | 0.00001 | 0.00105 |
| WP426 | Urea cycle and metabolism of amino groups | 20 | 3 | miR-122 | miR-1 miR-497 | 0.16500 | 3 | 1.00 | 0.00 | 0.00193 | 0.00315 |
| WP447 | Adipogenesis Mouse | 131 | 37 | miR-100 miR-223 miR-122 | miR-203 miR-1 miR-497 | 0.34900 | 8 | 0.13 | 0.88 | 0.00026 | 0.00319 |
| WP150 | DNA Replication | 41 | 3 | miR-223 | miR-203 miR-497 | 0.42900 | 5 | 1.00 | 0.00 | 0.00016 | 0.00376 |
| WP413 | G1 to S cell cycle control | 61 | 20 | miR-223 miR-122 | miR-203 miR-1 miR-497 | 0.10800 | 4 | 1.00 | 0.00 | 0.00734 | 0.00466 |
| WP458 | Inflammatory Response Pathway | 39 | 5 | miR-122 | miR-203 miR-1 miR-497 | 0.38300 | 4 | 0.50 | 0.50 | 0.00144 | 0.01021 |
| WP234 | Peptide GPCRs | 70 | 7 | miR-122 | miR-203 miR-1 | 0.16400 | 4 | 0.75 | 0.25 | 0.01182 | 0.01096 |
| WP85 | Focal Adhesion | 183 | 49 | miR-100 miR-223 miR-122 | miR-203 miR-1 miR-497 | 0.37200 | 8 | 0.88 | 0.13 | 0.00231 | 0.01274 |
| WP222 | Cytokines and Inflammatory Response (BioCarta) | 23 | 3 |  | miR-1 miR-497 | 0.34900 | 3 | 0.00 | 1.00 | 0.00291 | 0.01307 |
| WP373 | IL-3 Signaling Pathway | 95 | 34 | miR-451 miR-122 | miR-203 miR-1 miR-497 | 0.29200 | 5 | 0.40 | 0.60 | 0.00708 | 0.01692 |
| WP113 | TGF Beta Signaling Pathway | 50 | 19 | miR-223 | miR-203 miR-1 miR-497 | 0.37800 | 4 | 0.50 | 0.50 | 0.00361 | 0.01703 |
| WP252 | Androgen Receptor Signaling Pathway | 105 | 34 | miR-223 miR-122 | miR-203 miR-1 miR-497 | 0.27500 | 5 | 0.60 | 0.40 | 0.01066 | 0.02015 |
| WP460 | Blood Clotting Cascade | 18 | 1 |  | miR-203 | 0.62100 | 3 | 0.67 | 0.33 | 0.00141 | 0.02906 |
| WP512 | Id Signaling Pathway | 49 | 15 | miR-100 miR-223 | miR-203 miR-1 miR-497 | 0.33300 | 3 | 1.00 | 0.00 | 0.02385 | 0.04406 |

Combined *p-value* < 0.05. * Number of interactions between modulated miRNA and genes present in the pathway. ** Number of modulated mRNA associated with genes of the pathway.
