# Supplementary material for: MicroRNAs Profiling in Murine Models of Acute and Chronic Asthma: A Relationship with mRNAs Targets
Source: PLoS One. 2011 Jan 28;6(1):e16509. doi: 10.1371/journal.pone.0016509 (PMC3030602; doi:10.1371/journal.pone.0016509)
Supplement: Table S9 — MiRNAs/mRNAs regulatory pathways at IT using TargetScan. (DOC) [file pone.0016509.s010.doc]

| **Wikipathway** | **Pathway name** | **Total # of genes/pathway** | **miRNA/mRNA interactions*** | **miRna** | | ***p-value* (miRNA)** | **# of modul. mRNA in the pathway**** | **Proportions of mRna** | | ***p-value***  **(mRNA)** | **combined**  ***p-value*** |
| --- | --- | --- | --- | --- | --- | --- | --- | --- | --- | --- | --- |
|  |  |  |  | **Up** | **Down** |  |  | **Up** | **Down** |  |  |
| WP441 | Matrix Metalloproteinases | 25 | 8 |  | miR-29b miR-29c miR-218 miR-152 | 0.04200 | 5 | 1.00 | 0.00 | 0.00027 | 0.00012 |
| WP447 | Adipogenesis Mouse | 131 | 57 | miR-375 miR-150 miR-455 miR-155 | miR-450a-5p miR-223 miR-29b miR-29c miR-218 miR-152 | 0.15800 | 13 | 1.00 | 0.00 | 0.00002 | 0.00013 |
| WP252 | Androgen Receptor Signaling Pathway | 105 | 37 | miR-375 miR-150 miR-155 | miR-223 miR-29b miR-29c miR-218 miR-152 | 0.40400 | 13 | 1.00 | 0.00 | 0.00000 | 0.00023 |
| WP458 | Inflammatory Response Pathway | 39 | 11 |  | miR-29b miR-29c miR-152 | 0.06800 | 5 | 1.00 | 0.00 | 0.00221 | 0.00108 |
| WP385 | Myometrial Relaxation and Contraction Pathways | 159 | 45 | miR-375 miR-150 miR-155 | miR-450a-5p miR-223 miR-29b miR-29c miR-218 miR-152 | 0.46700 | 13 | 0.92 | 0.08 | 0.00012 | 0.00387 |
| WP151 | IL-5 Signaling Pathway | 65 | 15 | miR-375 miR-150 miR-155 | miR-29b miR-29c miR-218 | 0.64800 | 9 | 1.00 | 0.00 | 0.00002 | 0.00442 |
| WP572 | EGFR1 Signaling Pathway | 171 | 78 | miR-375 miR-150 miR-455 miR-155 | miR-450a-5p miR-223 miR-29b miR-29c miR-218 miR-152 | 0.30400 | 12 | 1.00 | 0.00 | 0.00083 | 0.00486 |
| WP258 | TGF-beta Receptor Signaling Pathway | 147 | 50 | miR-375 miR-150 miR-455 miR-155 | miR-223 miR-29b miR-29c miR-218 miR-152 | 0.60600 | 13 | 1.00 | 0.00 | 0.00005 | 0.00530 |
| WP523 | Regulation of Actin Cytoskeleton | 146 | 55 | miR-375 miR-150 miR-155 | miR-450a-5p miR-223 miR-29b miR-29c miR-218 miR-152 | 0.36500 | 11 | 1.00 | 0.00 | 0.00076 | 0.00645 |
| WP6 | Integrin-mediated cell adhesion | 97 | 34 | miR-375 miR-150 miR-455 miR-155 | miR-223 miR-29b miR-29c miR-218 miR-152 | 0.47600 | 9 | 1.00 | 0.00 | 0.00051 | 0.00900 |
| WP450 | IL-2 Signaling Pathway | 73 | 27 | miR-375 miR-150 miR-455 miR-155 | miR-450a-5p miR-223 miR-29b miR-29c miR-218 miR-152 | 0.54300 | 8 | 1.00 | 0.00 | 0.00034 | 0.00996 |
| WP373 | IL-3 Signaling Pathway | 95 | 32 | miR-375 miR-455 miR-155 | miR-450a-5p miR-29b miR-29c miR-218 miR-152 | 0.58600 | 9 | 1.00 | 0.00 | 0.00044 | 0.01391 |
| WP113 | TGF Beta Signaling Pathway | 50 | 20 | miR-155 | miR-223 miR-29b miR-29c miR-218 miR-152 | 0.57200 | 6 | 1.00 | 0.00 | 0.00115 | 0.02135 |
| WP412 | Oxidative Stress | 25 | 11 | miR-375 miR-155 | miR-223 miR-29b miR-29c miR-152 | 0.04700 | 2 | 1.00 | 0.00 | 0.11855 | 0.02168 |
| WP246 | TNF-alpha/NF-kB Signaling Pathway | 174 | 48 | miR-375 miR-150 miR-455 miR-155 | miR-223 miR-29b miR-29c miR-218 miR-152 | 0.31700 | 10 | 0.90 | 0.10 | 0.00898 | 0.02221 |
| WP539 | Wnt Signaling Pathway NetPath | 107 | 49 | miR-375 miR-150 miR-455 miR-155 | miR-223 miR-29b miR-29c miR-218 miR-152 | 0.28200 | 7 | 1.00 | 0.00 | 0.01406 | 0.02498 |
| WP108 | Mm Selenium metabolism/Selenoproteins | 47 | 21 | miR-375 miR-150 miR-155 | miR-223 miR-29b miR-29c miR-218 miR-152 | 0.01700 | 2 | 0.50 | 0.50 | 0.30835 | 0.03194 |
| WP544 | Circadian Exercise | 49 | 18 | miR-375 miR-155 | miR-223 miR-29b miR-29c miR-218 miR-152 | 0.29600 | 4 | 1.00 | 0.00 | 0.02893 | 0.04270 |
| WP93 | IL-4 signaling pathway | 58 | 20 | miR-375 miR-150 miR-455 miR-155 | miR-223 miR-29b miR-29c miR-218 miR-152 | 0.43000 | 5 | 1.00 | 0.00 | 0.01218 | 0.04302 |
| WP168 | Apoptosis Mechanisms | 79 | 37 | miR-375 miR-150 miR-455 miR-155 | miR-223 miR-29b miR-29c miR-218 miR-152 | 0.12100 | 4 | 1.00 | 0.00 | 0.11928 | 0.04839 |
| WP493 | MAPK signaling pathway | 133 | 36 | miR-375 miR-150 miR-455 miR-155 | miR-450a-5p miR-223 miR-29b miR-29c miR-218 miR-152 | 0.74500 | 10 | 1.00 | 0.00 | 0.00132 | 0.04847 |

Combined *p-value* < 0.05. * Number of interactions between modulated miRNA and genes present in the pathway. ** Number of modulated mRNA associated with genes of the pathway.
