# Supplementary material for: MicroRNAs Profiling in Murine Models of Acute and Chronic Asthma: A Relationship with mRNAs Targets
Source: PLoS One. 2011 Jan 28;6(1):e16509. doi: 10.1371/journal.pone.0016509 (PMC3030602; doi:10.1371/journal.pone.0016509)
Supplement: Table S10 — MiRNAs/mRNAs regulatory pathways at LT using TargetScan. (DOC) [file pone.0016509.s011.doc]

| **Wikipathway** | **Pathway name** | **Total # of genes/pathway** | **miRNA/mRNA interactions*** | **miRna** | | ***p-value* (miRNA)** | **# of modul. mRNA in the pathway**** | **Proportions of mRna** | | ***p-value***  **(mRNA)** | **combined**  ***p-value*** |
| --- | --- | --- | --- | --- | --- | --- | --- | --- | --- | --- | --- |
|  |  |  |  | **Up** | **Down** |  |  | **Up** | **Down** |  |  |
| WP571 | FAS pathway and Stress induction of HSP regulation | 38 | 49 | miR-30d miR-145 miR-143 miR-206 miR-346 miR-455 | miR-98 miR-26b miR-23a miR-23b miR-30b miR-25 miR-21 miR-27a miR-30c miR-92a miR-200b | 0.00000 | 1 | 0.00 | 1.00 | 0.46019 | 0.00000 |
| WP441 | Matrix Metalloproteinases | 25 | 19 | miR-30d miR-214 miR-206 | miR-29c miR-98 miR-26b miR-23a miR-23b miR-30b miR-21 miR-27a miR-30c miR-200b | 0.00300 | 3 | 0.67 | 0.33 | 0.00729 | 0.00012 |
| WP252 | Androgen Receptor Signaling Pathway | 105 | 134 | miR-30d miR-145 miR-143 miR-320 miR-214 miR-328 miR-206 miR-346 miR-197 | miR-29c miR-98 miR-26b miR-23a miR-23b miR-30b miR-25 miR-27a miR-30c miR-92a miR-200b | 0.03000 | 7 | 0.00 | 1.00 | 0.00155 | 0.00031 |
| WP493 | MAPK signaling pathway | 133 | 217 | miR-191 miR-30d miR-145 miR-99b miR-143 miR-320 miR-214 miR-328 miR-206 miR-346 miR-455 miR-197 | miR-29c miR-98 miR-26b miR-23a miR-23b miR-30b miR-25 miR-21 miR-27a miR-30c miR-92a miR-200b | 0.00200 | 5 | 0.00 | 1.00 | 0.06385 | 0.00093 |
| WP246 | TNF-alpha/NF-kB Signaling Pathway | 174 | 171 | miR-191 miR-30d miR-145 miR-99b miR-143 miR-320 miR-214 miR-206 miR-346 miR-455 miR-197 | miR-29c miR-98 miR-26b miR-23a miR-23b miR-30b miR-25 miR-21 miR-27a miR-30c miR-92a miR-200b | 0.00900 | 7 | 0.00 | 1.00 | 0.02274 | 0.00101 |
| WP544 | Circadian Exersice | 49 | 67 | miR-30d miR-145 miR-143 miR-320 miR-214 miR-328 miR-206 | miR-29c miR-98 miR-26b miR-23a miR-23b miR-30b miR-25 miR-21 miR-27a miR-30c miR-92a miR-200b | 0.00600 | 3 | 0.00 | 1.00 | 0.04410 | 0.00143 |
| WP168 | Apoptosis Mechanisms | 79 | 117 | miR-30d miR-145 miR-99b miR-143 miR-320 miR-214 miR-328 miR-206 miR-346 miR-455 | miR-29c miR-98 miR-26b miR-23a miR-23b miR-30b miR-25 miR-21 miR-27a miR-30c miR-92a miR-200b | 0.00300 | 3 | 0.00 | 1.00 | 0.13442 | 0.00322 |
| WP450 | IL-2 Signaling Pathway | 73 | 127 | miR-30d miR-145 miR-99b miR-143 miR-320 miR-214 miR-328 miR-206 miR-346 miR-455 miR-197 | miR-29c miR-98 miR-26b miR-23a miR-23b miR-30b miR-25 miR-21 miR-27a miR-30c miR-92a miR-200b | 0.00500 | 3 | 0.00 | 1.00 | 0.11312 | 0.00371 |
| WP274 | B Cell Receptor Signaling Pathway | 149 | 197 | miR-30d miR-145 miR-143 miR-320 miR-214 miR-328 miR-206 miR-346 miR-197 | miR-29c miR-98 miR-26b miR-23a miR-23b miR-30b miR-25 miR-21 miR-27a miR-30c miR-92a miR-200b | 0.00800 | 5 | 0.00 | 1.00 | 0.09298 | 0.00416 |
| WP519 | Proteasome Degradation | 78 | 52 | miR-30d miR-145 miR-143 miR-214 miR-328 miR-206 miR-455 miR-197 | miR-29c miR-26b miR-23a miR-23b miR-30b miR-25 miR-27a miR-30c miR-92a miR-200b | 0.00700 | 3 | 0.00 | 1.00 | 0.13078 | 0.00568 |
| WP567 | Eukaryotic Transcription Initiation | 40 | 19 | miR-30d miR-206 | miR-98 miR-26b miR-30b miR-27a miR-30c miR-200b | 0.00700 | 2 | 0.00 | 1.00 | 0.13520 | 0.00592 |
| WP297 | IL-7 Signaling Pathway | 42 | 82 | miR-30d miR-145 miR-143 miR-320 miR-214 miR-206 miR-197 | miR-29c miR-98 miR-26b miR-23a miR-23b miR-30b miR-25 miR-27a miR-30c miR-92a miR-200b | 0.00700 | 2 | 0.00 | 1.00 | 0.14624 | 0.00653 |
| WP93 | IL-4 signaling pathway | 58 | 88 | miR-30d miR-145 miR-143 miR-320 miR-214 miR-206 miR-455 miR-197 | miR-29c miR-98 miR-26b miR-23a miR-23b miR-30b miR-25 miR-21 miR-27a miR-30c miR-92a miR-200b | 0.00400 | 2 | 0.00 | 1.00 | 0.23921 | 0.00874 |
| WP426 | Urea cycle and metabolism of amino groups | 20 | 12 | miR-30d miR-214 miR-206 | miR-29c miR-98 miR-23a miR-23b miR-30b miR-30c | 0.00500 | 1 | 0.00 | 1.00 | 0.27700 | 0.01255 |
| WP373 | IL-3 Signaling Pathway | 95 | 148 | miR-30d miR-145 miR-143 miR-320 miR-214 miR-328 miR-206 miR-346 miR-455 miR-197 | miR-29c miR-98 miR-26b miR-23a miR-23b miR-30b miR-25 miR-21 miR-27a miR-30c miR-92a miR-200b | 0.01400 | 3 | 0.00 | 1.00 | 0.19683 | 0.01551 |
| WP488 | Alpha6-Beta4 Integrin Signaling Pathway | 65 | 81 | miR-30d miR-145 miR-99b miR-143 miR-320 miR-214 miR-206 miR-346 miR-197 | miR-29c miR-98 miR-26b miR-23a miR-23b miR-30b miR-25 miR-27a miR-30c miR-92a miR-200b | 0.00700 | 2 | 0.50 | 0.50 | 0.28099 | 0.01587 |
| WP385 | Myometrial Relaxation and Contraction Pathways | 159 | 195 | miR-191 miR-30d miR-145 miR-99b miR-143 miR-320 miR-214 miR-328 miR-206 miR-346 miR-197 | miR-29c miR-98 miR-26b miR-23a miR-23b miR-30b miR-25 miR-21 miR-27a miR-30c miR-92a miR-200b | 0.00200 | 3 | 0.00 | 1.00 | 0.47231 | 0.01857 |
| WP413 | G1 to S cell cycle control | 61 | 49 | miR-30d miR-145 miR-143 miR-320 miR-214 miR-206 | miR-29c miR-98 miR-26b miR-23a miR-23b miR-30b miR-25 miR-21 miR-27a miR-30c miR-92a miR-200b | 0.24100 | 4 | 0.00 | 1.00 | 0.01671 | 0.02268 |
| WP572 | EGFR1 Signaling Pathway | 171 | 291 | miR-191 miR-30d miR-145 miR-143 miR-320 miR-214 miR-328 miR-206 miR-346 miR-455 miR-197 | miR-29c miR-98 miR-26b miR-23a miR-23b miR-30b miR-25 miR-21 miR-27a miR-30c miR-92a miR-200b | 0.00200 | 3 | 0.00 | 1.00 | 0.52066 | 0.02283 |
| WP113 | TGF Beta Signaling Pathway | 50 | 87 | miR-30d miR-145 miR-143 miR-320 miR-214 miR-206 miR-346 miR-197 | miR-29c miR-98 miR-26b miR-23a miR-23b miR-30b miR-25 miR-21 miR-27a miR-30c miR-92a miR-200b | 0.02900 | 2 | 0.00 | 1.00 | 0.19196 | 0.02522 |
| WP458 | Inflammatory Response Pathway | 39 | 23 | miR-30d miR-145 miR-328 miR-206 | miR-29c miR-98 miR-26b miR-30b miR-25 miR-27a miR-30c miR-92a miR-200b | 0.05200 | 2 | 0.00 | 1.00 | 0.12975 | 0.02577 |
| WP456 | GPCRs -- Class B Secretin-like | 13 | 9 | miR-30d miR-143 | miR-29c miR-23a miR-23b miR-30b miR-30c miR-200b | 0.03600 | 1 | 1.00 | 0.00 | 0.19006 | 0.02919 |
| WP350 | p38 MAPK Signaling Pathway (BioCarta) | 28 | 52 | miR-30d miR-145 miR-143 miR-320 miR-214 miR-206 miR-346 | miR-29c miR-98 miR-26b miR-23a miR-23b miR-30b miR-25 miR-21 miR-27a miR-30c miR-92a miR-200b | 0.01000 | 1 | 0.00 | 1.00 | 0.36503 | 0.02945 |
| WP387 | IL-6 signaling pathway | 96 | 121 | miR-30d miR-145 miR-143 miR-320 miR-214 miR-206 miR-346 miR-455 miR-197 | miR-29c miR-98 miR-26b miR-23a miR-23b miR-30b miR-25 miR-21 miR-27a miR-30c miR-92a miR-200b | 0.00600 | 2 | 0.00 | 1.00 | 0.45853 | 0.03216 |
| WP10 | IL-9 Signaling Pathway | 23 | 31 | miR-30d miR-145 miR-143 miR-320 miR-214 miR-206 miR-455 miR-197 | miR-29c miR-23a miR-23b miR-30b miR-25 miR-27a miR-30c miR-92a miR-200b | 0.02600 | 1 | 0.00 | 1.00 | 0.31136 | 0.04254 |
| WP258 | TGF-beta Receptor Signaling Pathway | 147 | 201 | miR-30d miR-145 miR-143 miR-320 miR-214 miR-206 miR-346 miR-455 miR-197 | miR-29c miR-98 miR-26b miR-23a miR-23b miR-30b miR-25 miR-21 miR-27a miR-30c miR-92a miR-200b | 0.05200 | 4 | 0.00 | 1.00 | 0.21215 | 0.04321 |
| WP539 | Wnt Signaling Pathway NetPath | 107 | 168 | miR-191 miR-30d miR-145 miR-99b miR-143 miR-320 miR-214 miR-328 miR-206 miR-346 miR-455 miR-197 | miR-29c miR-98 miR-26b miR-23a miR-23b miR-30b miR-25 miR-27a miR-30c miR-92a miR-200b | 0.00800 | 2 | 0.00 | 1.00 | 0.51546 | 0.04687 |
| WP265 | Delta-Notch Signaling Pathway | 79 | 83 | miR-30d miR-145 miR-143 miR-320 miR-214 miR-328 miR-206 miR-346 miR-197 | miR-29c miR-98 miR-26b miR-23a miR-23b miR-30b miR-25 miR-21 miR-27a miR-30c miR-92a miR-200b | 0.10900 | 3 | 0.00 | 1.00 | 0.13442 | 0.04917 |

Combined *p-value* < 0.05. * Number of interactions between modulated miRNA and genes present in the pathway. ** Number of modulated mRNA associated with genes of the pathway.
